# Iron-induced demethoxylation of lignin as an important source for methanol and its oxidation products in the environment

**DOI:** 10.1038/s41467-026-76679-x

**Published:** 2026-08-13

**Authors:** Jonas Hädeler, Gunasekaran Velmurugan, Rebekka Lauer, Isabel Hanstein, Julia Wenhuda, Rejith Radhamani, Peter Comba, Frank Keppler

**Affiliations:** 1 Institute of Earth Sciences, Heidelberg University, Heidelberg, Germany; 2 Anorganisch-Chemisches Institut, Heidelberg University, Heidelberg, Germany; 3 Department of Chemistry, National Institute of Technology—Tiruchirappalli, Tiruchirappalli, Tamil Nadu India; 4 Interdisciplinary Center for Scientific Computing, Universität Heidelberg, Heidelberg, Germany; 5 Heidelberg Center for the Environment (HCE), Heidelberg University, Heidelberg, Germany

**Keywords:** Geochemistry, Geochemistry

## Abstract

Lignin, the largest reservoir of aromatic carbon in plants and soils, undergoes well-characterized biological demethylation by fungi and bacteria, and is industrially processed under harsh conditions to generate bio-based aromatics. However, the abiotic fate of lignin-derived methoxy groups under environmentally relevant conditions remains unresolved. Here, we report an iron-mediated demethoxylation mechanism involving direct aromatic C–O bond cleavage under ambient conditions. Using Fe^II^/Fe^III^ species and H_2_O_2_, we demonstrate the efficient release of entire methoxy groups from lignin monomers and methoxyphenols, forming methanol and subsequently formaldehyde. Isotopic labeling unequivocally shows that the complete –OCH_3_ group is liberated intact. Density functional theory calculations support an exergonic demethoxylation pathway with low activation energy (43 kJ/mol) and favorable thermodynamics. Soil laboratory incubation experiments with lignin and diverse natural soils rich in iron and methoxylated organic matter confirm that this abiotic pathway operates under environmentally relevant wet–dry conditions. These findings establish iron-induced demethoxylation as a abiotic process, linking lignin turnover to microbial C_1_ metabolism and soil carbon cycling, with implications for atmospheric trace gas fluxes.

## Abstract (web summary)

Iron-mediated demethoxylation releases methanol from lignin under environmentally relevant conditions, providing new insights into abiotic lignin degradation, microbial one-carbon metabolism, and soil carbon cycling.

## Introduction

**Fig. 1:**
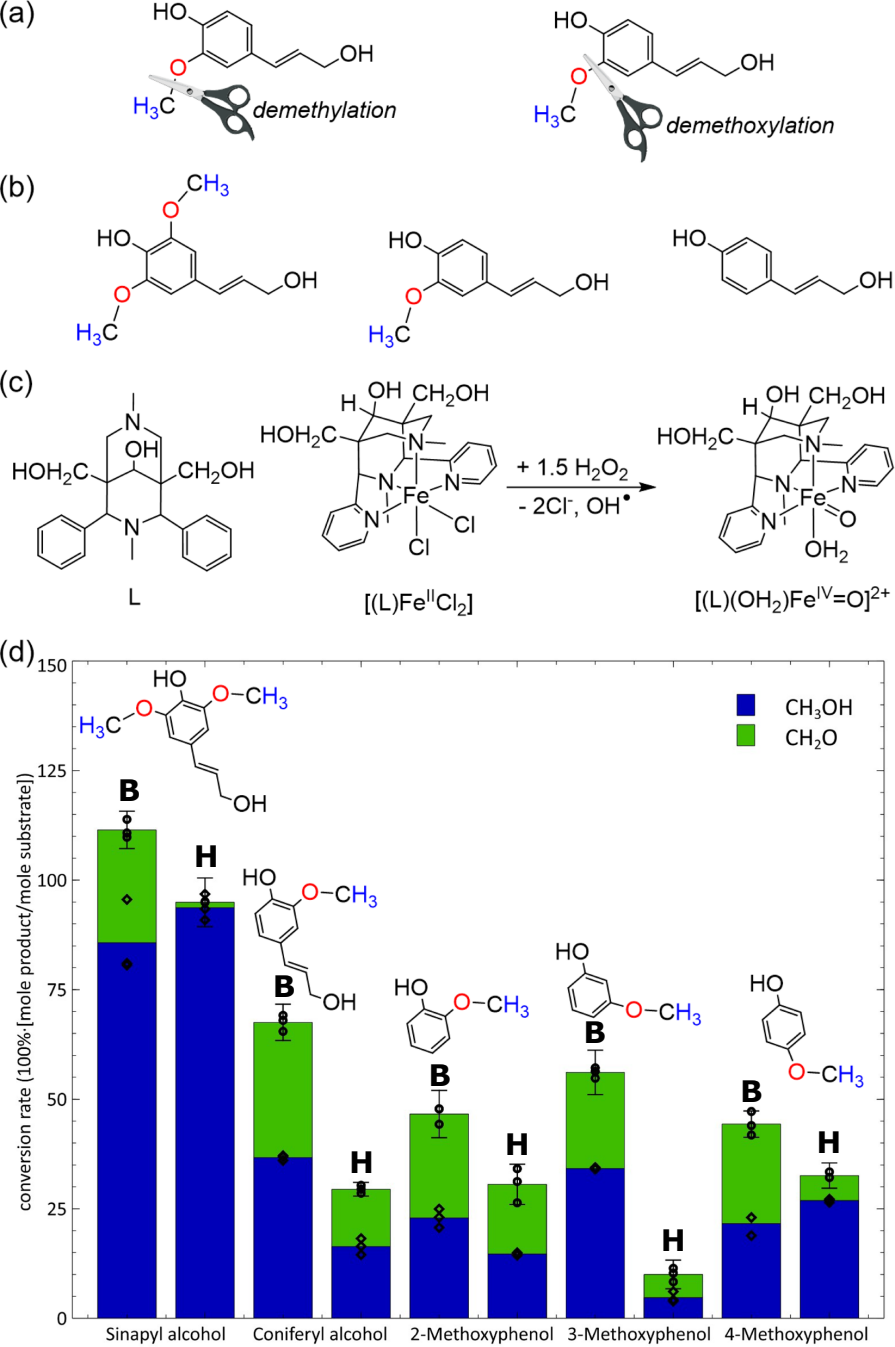
Conversion rates of monolignols and methoxyphenols to CH_3_OH and CH_2_O using Fe^II^/Fe^III^ species and H_2_O_2_. **a** Visualization at coniferyl alcohol of the two possible processes “demethylation” and “demethoxylation”; for each, there are various possible pathways, and these are discussed in Fig. 2 and the corresponding text. **b** Structures of the three monolignols (from left to right) sinapyl-, coniferyl-, and p-coumaryl alcohol. **c** Bispidine ligand structure, structure of the active oxidant [(L)(OH_2_)Fe^IV^ = O]^2+^ and its formation from the iron(II) precursor under Fenton-type conditions. **d** Formation of CH_3_OH and CH_2_O from monolignols and methoxyphenols in experiments mediated by 10 µmol [(L)Fe^II^Cl_2_], (bar **B**) or Fe_2_O_3_, (bar **H**; Fig. S1 shows the same data in another scale). Reactions were performed in 10 mL H_2_O, including 25 µmol substrate, 200 µmol H_2_O_2_, and 0.05 µmol HOtf acid. All provided data were evaluated at 22 °C, ambient pressure (1013 mbar), and under N_2_/O_2_ atmosphere after 48 h reaction time. Conversion rates were related to one methoxy group (note: sinapyl alcohol contains two methoxy groups). Error bars represent the standard deviation (SD) of total CH_3_OH and CH_2_O formation, based on the mean of three independent experiments (*n* = 3), each measured in triplicate. Diamonds (CH₃OH) and circles (CH₂O) indicate the mean value of each independent experiment.

Lignin is the largest reservoir of aromatic carbon in plants and constitutes approximately 30% of Earth’s terrestrial non-fossil organic carbon^1,2^. It is a complex polymer derived from three primary phenolic monomers—sinapyl, coniferyl, and p-coumaryl alcohol (Fig. 1b) ─, which differ in the number of ortho-methoxy substituents (–OCH_3_). As a consequence, lignin contains substantial amounts of methoxy groups, with contents of up to 20% in the polymer, corresponding to approximately 4–6% of total carbon in woody biomass^3^. Upon plant decay, lignin and its transformation products contribute significantly to soil organic carbon (SOC), of which approximately 2400 Gt are stored globally to a depth of 2 m^4^. A considerable fraction of this pool consists of lignin-derived aromatic structures retaining methoxy functionalities.

Traditionally, lignin decomposition has been attributed to biological processes mediated by fungi and bacteria, particularly via ligninolytic peroxidases and oxidative enzymes^4,5^. However, growing evidence indicates that abiotic redox processes, especially those involving iron minerals, also play an important role in regulating soil organic matter dynamics. Iron-mediated reactions can generate reactive oxygen species (ROS), including hydroxyl radicals formed through Fenton-type chemistry, or they can directly attack organic substrates, oxidizing phenolic compounds such as lignin independently of enzymatic catalysis, leading to a substantial enhancement of CO_2_ production in redox-fluctuating tropical soils^6,7^. Iron oxidation and reduction-cycles have further been shown to stimulate organic matter decomposition in humid forest soils and to counteract mineral protection mechanisms that otherwise stabilize organic carbon^8^. Comparative studies indicate that iron, together with manganese, represents a major driver of oxidative soil organic matter degradation—particularly of phenolic substrates—often acting alongside or independent of enzymatic pathways^8^. A recent study emphasizes the tight coupling between iron cycling and organic matter degradation across fluctuating redox interfaces in soils and sediments^9^. Collectively, these findings suggest that iron-mediated oxidative processes may contribute substantially to soil carbon turnover. Yet, the molecular-scale mechanisms linking iron redox chemistry to the transformation of specific lignin-derived functional groups remain insufficiently resolved.

Methoxy groups in lignin represent a chemically distinct and globally abundant pool of low-valent carbon. These groups are known precursors for various one-carbon (C_1_) compounds, including methane (CH_4_), methanol (CH_3_OH), formaldehyde (CH_2_O), and chloromethane (CH_3_Cl)^10^. While biotic pathways for C_1_ compound formation are well established, the abiotic production of oxygenated C_1_ compounds from aromatic methoxy groups under environmentally relevant conditions has not been studied thoroughly. In particular, it has not been demonstrated so far that the entire methoxy group can be cleaved from lignin-derived aromatics under ambient soil conditions.

In previous work, we have reported iron-mediated abiotic formation of C_1_ compounds from sulfur- and nitrogen-bound methyl groups using well-defined Fe^II/III^ model systems and natural iron oxides^10,11^. In those systems, high-valent iron-oxido species promote demethylation via oxygen atom transfer (OAT), leading to methyl radical formation. With acetic acid derivatives, an alternative mechanism was established, also induced by high-valent iron-oxido species, i.e., hydrogen atom abstraction (HAA), and also generating methyl radicals^12^. These form subsequently CH_4_, CH_3_OH, or CH_2_O depending on reaction conditions. Importantly, these pathways involve cleavage of heteroatom or carbon–methyl bonds (e.g., H_3_C–C, H_3_C–S or H_3_C–N) and proceed through methyl radical intermediates. However, these radical-based demethylation mechanisms do not account for the excessively high ratios of CH_3_OH and CH_2_O relative to CH_4_ observed in soil incubations^13^. This discrepancy suggests the involvement of alternative precursors such as oxygen-bound methyl groups (e.g., methoxy groups in lignin) and of mechanistically distinct pathways that do not rely on methyl radical intermediates.

To date, demethoxylation of aromatic methoxy groups, defined as cleavage of the C_aromatic_–O bond and releasing the entire –OCH_3_ (methoxy) group, has primarily been reported under harsh conditions ( > 285 °C, >0.5 MPa, hydrogen atmosphere), often employing heterogeneous catalysts and simple model substrates such as 2-methoxyphenol^14,15^ (see Fig. 1a for the distinction between demethylation and demethoxylation; note that both processes can occur via homo- or heterolytic bond cleavage)^13,16–18^. Whether demethoxylation can occur under ambient, environmentally relevant conditions and contribute to soil C_1_ cycling has remained unresolved.

Here, we report an abiotic demethoxylation pathway for lignin-derived methoxy groups that operates under ambient conditions (22 °C, 1013 mbar, air), is mediated by iron species and H_2_O_2_, and is fundamentally distinct from previously described radical-based demethylation mechanisms^10,12,13,19^. Using both the well-defined nonheme iron model complex ([(L)Fe^II^Cl_2_], Fig. 1c) and the naturally abundant iron oxide hematite (Fe_2_O_3_), we demonstrate that lignin monomers and methoxyphenols release CH_3_OH as the primary product, followed by oxidation to CH_2_O. Isotope labeling experiments unequivocally show that the entire methoxy group is cleaved, and quantum-chemical calculations afford the detailed mechanistic pathway.

Finally, we extend these mechanistic findings to natural systems by demonstrating that sterilized field soils rich in iron minerals and lignin-derived organic matter produce measurable amounts of CH_3_OH and CH_2_O under environmentally relevant wet–dry cycles. Together, these results identify iron-induced demethoxylation as an overlooked abiotic transformation linking lignin turnover, soil redox dynamics, and the production of oxygenated C_1_ compounds in terrestrial ecosystems.

## Results

The monolignols sinapyl- and coniferyl alcohol, along with 2-, 3-, and 4-methoxyphenol, were incubated with H_2_O_2_ and the bispidine model complex [(L)Fe^II^Cl_2_] or Fe_2_O_3_, typically in a molar ratio of 25:200:10 in ultra-pure water (see Fig. 1c for structural formulae). We use the bispidine model system in experiments and computational work because its reactivity and mechanistic pathways are well-understood^20–22^. Reactions were conducted for 48 h at ambient temperature and atmospheric pressure (22 °C, 1013 mbar, 20.8% O_2_; for details see methods section and SI). The solution pH was approx. 2.3 and kept constant by the addition of trifluormethanesulfonic acid (triflic acid, HOtf), i.e., at conditions that were optimized in previous studies to support this class of transformations and to align with the analytical techniques employed^11,13^. In these settings, the high-valent bispidine-iron species and Fe_2_O_3_ may act catalytically. However, given the concentrations used in our experiments and the abundance of iron in natural soils, catalytic turnover is not required for substantial product formation, i.e., the iron oxidant is not a limiting factor. Importantly, the experimental conditions are environmentally relevant but obviously different from those required for industrial and enzymatic catalysis.

Under the applied conditions, all methoxylated aromatic compounds investigated here produced substantial amounts of CH_3_OH and CH_2_O (Fig. 1c, Fig. S1), with total conversion rates (100%·[mole product/mole substrate]) ranging from 10 to 110% (Fig. 1c) and mass-based yields of 25.0–167 mg/g_substrate_ (Fig. S1). The product ratio in experiments with [(L)Fe^II^Cl_2_] (B) and Fe_2_O_3_ (H) is similar, indicating a comparable mechanism, but the total yield is generally higher for the bispidine systems. This may be due to the known higher reactivity of the bispidine complexes with respect to other model systems in general and specifically to the iron aqua complexes^22^. Note that we anticipate all reactions to reach completion within 48 h (see^13^ for kinetic data). An important observation, shown in Fig. 1c, is that the monolignol synapyl alcohol with two methoxy groups produces approximately twice as much CH_3_OH and CH_2_O as coniferyl alcohol.

Control experiments using 2-methoxyphenol in the absence of H_2_O_2_ and iron species yielded only trace amounts of CH_3_OH and CH_2_O (Fig. S2). Likewise, phenolic substrates lacking methoxy substituents produced negligible amounts of CH_3_OH and only low levels of CH_2_O, even in the presence of H_2_O_2_ and iron (Fig. S3). Importantly, no CH_4_ and C_2_H_6_ were detected when HOtf was replaced with ascorbic acid (Fig. S4), indicating that CH_3_OH and CH_2_O formation does not proceed via homolytic cleavage of the O–CH_3_ bond forming methyl radicals.

**Fig. 2:**
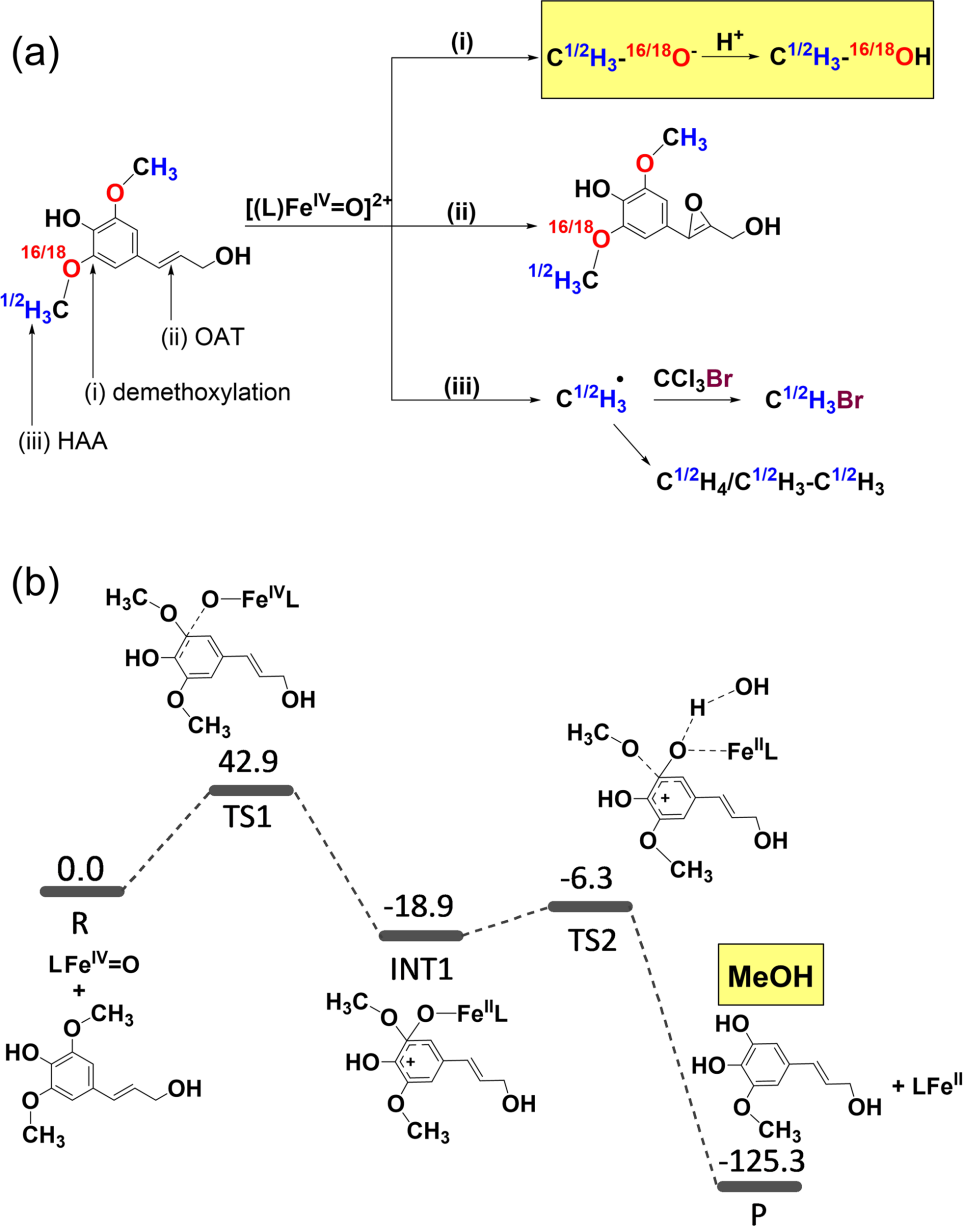
Isotopic constraints and computed reaction pathway for the iron-oxido–mediated demethoxylation of monolignols. **a** Isotopic constraints on possible pathways for the reaction with monolignols and computed potential energy surface of [(L)(OH_2_)Fe^IV^═O]^2+^ with synapyl alcohol. **b** Computed potential energy surface (UB3LYP-D3) for the reaction of [(L)(OH_2_)Fe^IV^═O]^2+^ with sinapyl alcohol, illustrating key intermediates and transition states along the proposed demethoxylation pathway.

To test the hypothesis that the complete OCH_3_ group of methoxylated aromatic compounds serves as the direct precursor of the observed oxygenated C_1_ products CH_3_OH and CH_2_O, we conducted a series of isotopic labeling experiments. These include the use of ^2^H- and ^18^O-labeled methoxy groups in 2-methoxyphenol, as well as reactions performed with H_2_^18^O_2_ and ^18^O_2_ as alternative oxygen sources (see Fig. 2a, Figs. S5–S9). The experiments with isotopically labeled 2-methoxyphenol demonstrate that the three hydrogen atoms of the methyl group and the oxygen atom of the methoxy group are incorporated into the produced CH_3_OH (Figs. S5, S6), unambiguously identifying the OCH_3_ group as its source. Similarly, CH_2_O retained the ^2^H labels (Fig. S9), and is therefore most likely a result of overoxidation of CH_3_OH^13^, confirming its origin from the methoxy group. In contrast, when H_2_^18^O_2_ or ^18^O_2_ was used in the oxidation reactions, no incorporation of ^18^O into CH_3_OH was observed (Figs. S7, S8), indicating that the oxygen atoms in the C_1_ products do not originate from atmospheric O_2_ or the oxidant H_2_O_2_. This is fundamentally distinct from previously described work^10,11^ where the demethylation of sulfur- and nitrogen-bound methyl groups via methyl radicals led to incorporation of ^18^O from both H_2_^18^O_2_ and ^18^O_2_ into CH_3_OH and CH_2_O. Additionally, alkyl trapping experiments with CCl_3_Br revealed no formation of bromomethane (Fig. S10), confirming the absence of free methyl radicals. This strongly supports a direct release mechanism of the intact OCH_3_ group from the aromatic precursor, rather than a methyl radical-based oxidative pathway. Therefore, a range of possible mechanisms were evaluated by an extensive DFT analysis.

The three major positions for attack of the Fe^IV^ = O oxidant to monolignols are shown in Fig. 2a, and these have all been studied by DFT using [(L)(OH_2_)Fe^IV^═O]^2+^ as catalyst (see “Methods” section for the computational approaches used and the SI for further details). As expected^21–23^, DFT predicts the quintet spin state to be the ground state of [(L)(OH_2_)Fe^IV^═O]^2+^, with a triplet state lying higher in energy by 14.2 kJ/mol. DFT generally has problems to accurately compute spin state energies, and a margin of around 15 kJ/mol is close to the error limit; from experiment and bench-mark quantum-chemical calculations, it emerges that the triplet/quintet gap in the system discussed here is very small^21,24^. From earlier computational work on similar systems and details in the SI^10,12,13,19,25,26^ it appears that (i) demethoxylation, (ii) epoxidation, and (iii) HAA at the methoxy or the methylene groups (Fig. 2a) all have relatively low activation barriers (energetics for all pathways are discussed in the SI, Figs. S20–S29), but demethoxylation is most efficient: the barrier for demethoxylation with approx. 43 kJ/mol (Fig. 2b) is lower than that for HAA at the methoxy group (55 kJ/mol, Figure S21) and also slightly lower than that for OAT to the double bond (approx. 46 kJ/mol, Fig. S22)^25,26^. It therefore emerges that the main ferryl-induced degradation pathway of monolignols is demethoxylation, induced by the electrophilic attack of the Fe^IV^ = O center at the aromatic carbon bearing the OMe substituent, and eventually leading to a substitution of the OMe^-^ by an OH^-^ group, originating from the ferryl oxidant (pathway i), and others, possibly leading to CH_3_ radicals that subsequently produce MeOH and other follow-up products^10,11,13^, may be minor additional contributors to the observed products. This precisely agrees with the experimental observations. Importantly, the energetically preferred mechanism, visualized with the PES in Fig. 2b, also suggests that there are similar pathways for the formation of MeOH and follow-up products (e.g., CH_2_O) in the absence of iron salts, and that the efficiency increases with increasing driving force (H_2_O_2_ < Fe_2_O_3_/H_2_O_2_ < [(L)Fe^II^Cl_2_]/H_2_O_2_; see SI for details).

To assess the environmental relevance of the proposed demethoxylation observed from monolignols and methoxyphenols, we performed experiments using the more complex polymer lignin (methoxy content: 9.3%) as a substrate, in combination with added Fe_2_O_3_ and H_2_O_2_ under the same conditions as the model compound studies (Fig. S11). This yielded 268 ± 39.0 µg of CH_3_OH and CH_2_O per gram of lignin, corresponding to a conversion of 5.4% of the total lignin methoxy content.

In the next step, we investigated a diverse set of field soil samples representing different soil types, spanning a wide range of iron content (0.15–4.14 w%), total organic carbon (TOC, 0–16.2 w%), methoxy group content (0.004–0.423 w%), and pH values (3.2–7.9; Table S1). A subset of 11 homogenized and sterilized soil samples was incubated in ultrapure water under ambient atmospheric (oxic) conditions, without the addition of external Fe_2_O_3_ or H_2_O_2_, in order to trigger iron-mediated oxidative processes.

**Fig. 3:**
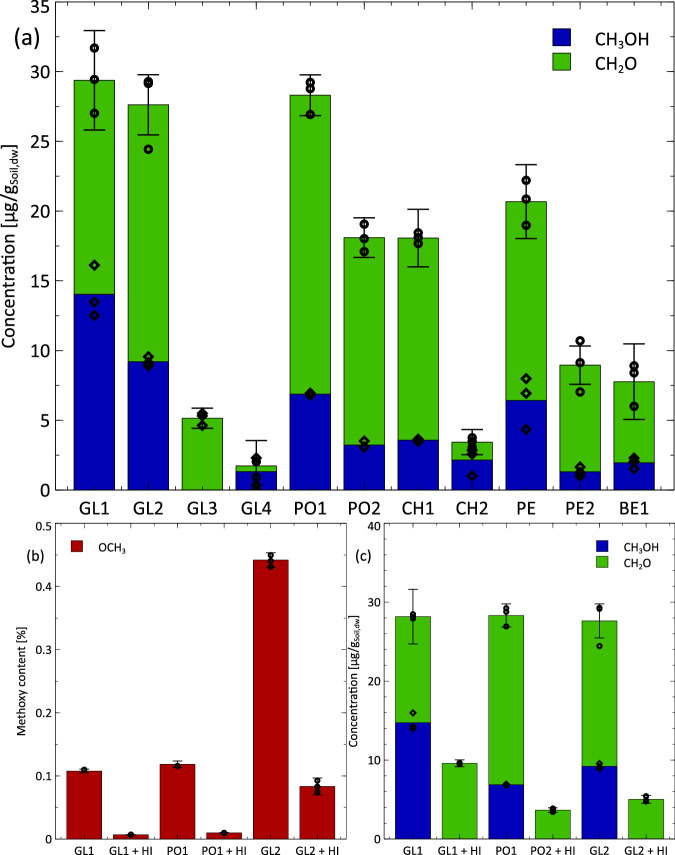
Abiotic formation of CH_3_OH and CH_2_O from soils and the relationship with the content of soil methoxy groups. **a** Abiotic formation of CH_3_OH and CH_2_O from homogenized and sterilized soil samples incubated under sterile, aqueous conditions with pH ranging from 3.2 to 7.9. Soils (5 g each) were suspended in ultrapure water (10 mL) and incubated for 48 h at ambient temperature and pressure (22 °C, 1013 mbar, under N_2_/O_2_ atmosphere). Soil types include Gley soils (GL1 to GL4), Podzol–brown earth (PO1 and PO2), Chernozem–para-brown earth (CH1 and CH2), Pelosol–brown earth (PE1 and PE2), and brown earth (BE1). **b** Methoxy group content in soils (30 g) GL1 (0–20 cm), PO1 (0–10 cm), and GL2 (0–10 cm) before and after treatment with hydroiodic acid (HI, 25 ml; 57 % at 105 °C for 2 h). **c** Formation of CH_3_OH and CH_2_O in the same soils (5 g in 10 ml ultrapure water at 22 °C, 1013 mbar, and under N_2_/O_2_ atmosphere) before and after chemical removal of methoxy groups. Error bars represent the SD from *n* = 3 independent measurements for methoxy content (**b**), and total CH_3_OH and CH_2_O formation, based on the mean of three independent experiments (*n* = 3), each measured in triplicate. Diamonds (CH₃OH) and circles (CH₂O) indicate the mean value of each independent experiment (**a** and **c**).

The concentrations of dissolved iron species in the aqueous phase were comparable to those used in the model compound experiments (see SI and^13^). Although H_2_O_2_ concentrations could not be directly quantified under these conditions, previous studies indicate that wetting of dry soils can generate H_2_O_2_ in the range of 500–1000 µmol/kg^27^, i.e., substantially lower amounts than in the model systems. Despite these lower oxidant levels, clear abiotic formation of CH_3_OH and CH_2_O was observed across all soil samples (Fig. 3a), with concentrations ranging from 0.0 to 14.0 ± 1.5 µg g⁻¹ soil dry weight (dw) for CH_3_OH and from 0.40 ± 0.53 to 15.3 ± 1.9 µg g⁻¹ (dw) for CH₂O.

The amounts of CH_3_OH and CH_2_O produced showed positive correlations with both methoxy group content (*R*² = 0.59; Fig. S12) and TOC (*R*² = 0.64; Fig. S12), while TOC itself strongly correlated with the methoxy group content (*R*² = 0.88; Fig. S12). These relationships suggest that lignin-derived methoxy groups constitute a major precursor pool for abiotic C_1_ compound formation in soils. On average, 0.13–1.36% of the soil methoxy pool was converted within a single 48 h incubation period.

To directly test the role of methoxy groups, we selectively removed them from chosen soil samples using the Zeisel demethoxylation method^28^ (Fig. 3b). This treatment completely suppressed CH_3_OH formation and reduced CH_2_O production by up to 83% (Fig. 3c), providing strong evidence that methoxy groups are the primary precursors of the observed oxygenated C_1_ compounds. Complementary isotopic labeling experiments using ^18^O and ^2^H labeled 2-methoxyphenol further demonstrated that HI-treated soils retained the capacity to convert externally supplied methoxy substrates into CH_3_OH (Fig. S13), confirming that the same mechanism operates in soil systems as in the model compounds.

**Fig. 4:**
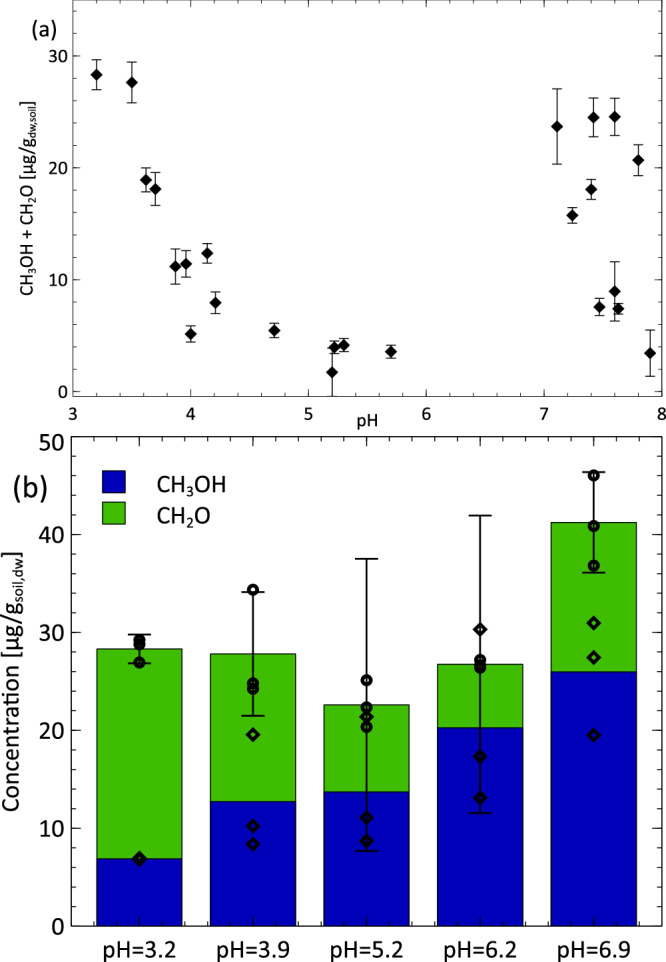
Relationship between CH_3_OH and CH_2_O formation and pH in all soil experiments and abiotic formation of CH_3_OH and CH_2_O in soil PO1 experiments at different pH-values. **a** Relationship of the pH (*n* = 1) with the sum of CH_3_OH and CH_2_O concentrations (*R*² = 0.00045 and *p* value = 0.92(one-sided)) in investigated soil samples (Table S1). **b** Formation of CH_3_OH and CH_2_O from soil sample PO1 incubated under sterile aqueous conditions with pH adjustments with NaOH (3.2–7.9). Soils (5 g each) were suspended in ultrapure water (10 mL) and incubated for 48 h at ambient temperature and pressure (22 °C, 1013 mbar, 20.8% O_2_). Error bars represent the SD of total CH_3_OH and CH_2_O formation, based on the mean of three independent experiments (*n* = 3), each measured in triplicate. Diamonds (CH_3_OH) and circles (CH_2_O) indicate the mean value of each independent experiment.

The investigated soil samples spanned a pH range of 3.2–7.9 (Table S1), representative of many terrestrial environments. Across this range, no significant correlation was observed between pH and the total yield of CH_3_OH and CH_2_O (*R*^2^ = 0.00045; Fig. 4a), indicating that overall abiotic C_1_ formation is largely independent of soil pH within this range. In addition, controlled pH adjustment experiments using soil sample PO1 revealed systematic changes in product distribution (Fig. 4b): CH_3_OH concentrations increased with increasing pH, whereas CH_2_O concentrations generally decreased, except for a slight increase at pH 6.9. The total yields of CH_3_OH and CH_2_O remained relatively constant across treatments, within experimental uncertainty, suggesting that pH primarily influences the partitioning between CH_3_OH and CH_2_O rather than the overall extent of methoxy group conversion.

When fresh, untreated, and thus biologically active soil samples were suspended in water, only negligible amounts of CH_3_OH and CH_2_O were detected, compared to the concentrations in dried and sterilized soils. This difference reflects the widespread presence of methylotrophic microorganisms in soils, which rapidly utilize CH_3_OH and CH_2_O as carbon and energy sources^29–32^. To further investigate CH_3_OH consumption under biologically active conditions, we performed experiments, in which CH_3_OH (200 µg/g_soil,dw_) was added to untreated soil samples. CH_3_OH concentrations declined steadily over time and were completely depleted within 15–21 days (Fig. S16), demonstrating efficient microbial turnover of CH_3_OH under near-natural conditions. To directly confirm microbial consumption of CH_3_OH, we inoculated sterilized soil samples—previously shown to produce CH_3_OH and CH_2_O abiotically—with *Methylorubrum extorquens*, a well-characterized methylotrophic bacterium capable of metabolizing oxygenated C_1_ compounds. Following incubation, CH_3_OH was no longer detectable, while bacterial growth was observed (Fig. S17), confirming that biologically active systems efficiently consume abiotically produced methanol.

**Fig. 5:**
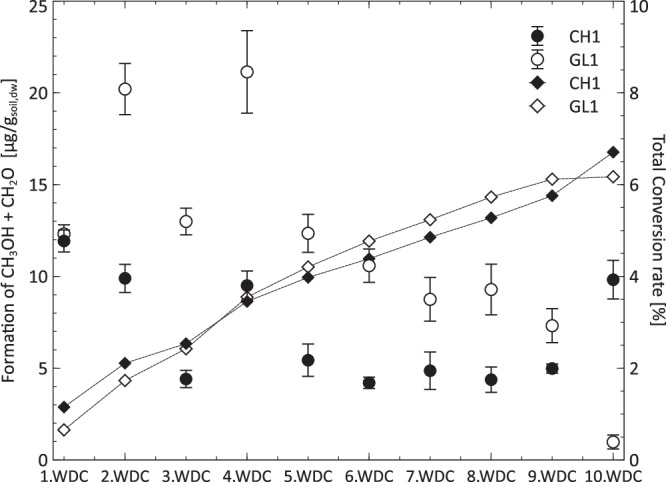
Formation of CH_3_OH and CH_2_O during repeated wetting–drying cycles of soils incubated under oxic conditions. CH_3_OH and CH_2_O production during ten consecutive wetting–drying cycles in soils CH1 and GL1, along with cumulative conversion of soil methoxy content. For each cycle, 5 g of dried soil was suspended in 10 mL of ultrapure water and incubated for 48 h at 22 °C, 1013 mbar, and under N_2_/O_2_ atmosphere. After incubation, the soils were dried at 105 °C for at least 24 h prior to rewetting. Error bars represent the SD of total CH_3_OH and CH_2_O conversion, based on the mean of three independent experiments (*n* = 3), each measured in triplicate.

To investigate the long-term potential of soils to produce CH_3_OH and CH_2_O, two contrasting soil types—CH1 (Gleysol) and GL1 (Chernozem/Para-brown earth)—were selected from the full dataset (Table S1). These soils represent typical temperate environments and differ in physicochemical properties, including TOC, iron, and methoxy content, thereby allowing assessment of the persistence and robustness of abiotic C_1_ formation under repeated wetting–drying cycles (WDC). The soils were subjected to consecutive WDCs under oxic conditions (Fig. 5), each consisting of a 48 h incubation period followed by drying and rewetting. During the first five cycles, CH_3_OH and CH_2_O production remained relatively constant, ranging between 5 and 20 µg g⁻¹ soil dry weight (dw). In subsequent cycles, a consistent decline in CH_3_OH and CH_2_O production was observed. The cumulative amounts produced over ten WDCs reached 64.0 µg/g_soil,dw_ and 121 µg/g_soil,dw_ for CH_3_OH and CH_2_O, respectively. When related to the initial methoxy content of the two soils (0.10 and 0.17 w %), this corresponds to total conversions of 6.2% and 6.7% (Fig. 5). These results demonstrate that soils can sustain the abiotic formation of oxygenated C_1_ compounds across multiple wetting–drying cycles. The decline in later cycles likely reflects not only the progressive depletion of available methoxy groups but also limitations in oxidant availability in the soils (Figs. S14, S15).

Together, these results demonstrate that iron-mediated demethoxylation of aromatic methoxy groups operates from well-defined monolignols to complex lignin substrates and natural soils. In the following section, we discuss the mechanistic implications of these findings and their potential role in soil carbon cycling.

## Discussion

The experiments with well-defined lignin monomers and methoxyphenols establish an abiotic pathway for the transformation of aromatic methoxy groups into oxygenated C_1_ compounds under environmentally relevant conditions. To our knowledge, this reaction has not previously been described in preparative chemistry and may therefore be of potential industrial interest. Under the applied conditions, all methoxylated aromatic substrates investigated here produced substantial amounts of CH_3_OH and CH_2_O, in some cases approaching near complete conversion of the aromatic methoxy substituents, demonstrating the high efficiency of iron-mediated demethoxylation and supporting the hypothesis that oxygenated C_1_ formation is directly linked to the presence of aromatic methoxy groups. The highest product yields were observed for substrates containing multiple methoxy substituents, consistent with the postulate that the formation of oxygenated C₁ compounds is directly linked to the presence of aromatic methoxy groups. The experiments with well-defined lignin monomers and methoxyphenols establish an abiotic pathway for the transformation of aromatic methoxy groups into oxygenated C_1_ compounds under environmentally relevant conditions.

Enzymatic demethylation reactions generally involve heme- or nonheme iron enzymes such as cytochrome P450 and α-ketoglutarate-dependent hydroxylases^33,34^, and model systems such as the bispidines discussed here have been studied extensively to evaluate reaction mechanisms and develop catalysts of industrial interest^22,33–35^. Reactions of high-valent iron-oxido species have also been discovered to be of relevance for environmental transformations, with two types of demethylation processes involving C-H activation (HAA, as in acetic acid^12,19^) or oxygen atom transfer (OAT) to methyl-substituted heteroatoms^10,11,13^, both producing CH_3_ radicals. Our results discussed here indicate that there is an additional, fundamentally different mechanism. The marginal amounts of CH_4_ and C_2_H_6_ formation in our experiments, which are expected products of methyl radical recombination or reduction^13^, demonstrate that radical-mediated demethylation does not dominate under the investigated conditions. Instead, isotopic labeling experiments unequivocally show that the entire methoxy group is released intact as CH_3_OH, providing direct experimental evidence for a demethoxylation process rather than demethylation.

Computational analyses further support this interpretation and suggest that the transformation proceeds via substitution of the entire OCH_3_ group by a hydroxide, where the oxygen atom originates from the Fe^IV^ = O species. The cleavage of the oxygen-carbon bond is, as expected, heterolytic^15^, and the driving force is the reformation of the aromatic system. Formally, this is a 2-electron redox process, releasing Fe^2+^, which in the catalytic cycle is reoxidized to Fe^IV^ = O, and the methoxylate captures a proton from the solvent. The CH_3_OH product may subsequently be oxidized to CH_2_O under more strongly oxidizing conditions. The calculated activation barriers are low, and the reaction pathway is exergonic, consistent with the experimentally observed reactivity under ambient temperature and pressure. Together, the experimental and theoretical results therefore indicate that iron-mediated oxidative processes can selectively transform methoxy groups bound to aromatic structures without generating radical intermediates.

Importantly, the reactivity observed in these controlled model systems directly targets the methoxy functional group, a ubiquitous structural motif in lignin and lignin-derived soil organic matter^2^. Because the mechanism operates at the level of this functional group rather than requiring a specific macromolecular structure, it provides a chemically plausible pathway for the oxidative transformation of a broad spectrum of lignin-derived materials present in soils. This functional-group–based perspective provides the mechanistic foundation for extending the observations from simple model compounds to more complex lignin substrates and ultimately to natural soil systems.

While the experiments with monolignols and methoxyphenols provide mechanistic insight into iron-induced demethoxylation at the molecular level, natural environments contain more complex lignin-derived materials. In soils, lignin rarely occurs as intact polymeric lignin but rather as partially degraded fragments and lignin–carbohydrate systems that are incorporated into soil organic matter. Importantly, these materials retain a substantial fraction of aromatic methoxy groups, which represent the reactive functional moiety targeted in the mechanism described here.

To evaluate whether the reaction extends beyond simple model compounds, we performed experiments using polymeric lignin as a substrate under the same iron-mediated oxidative conditions applied in the model studies. These incubations produced around 250 µg of CH_3_OH and CH_2_O per gram of lignin, corresponding to a conversion of approximately 5.4% of the total methoxy content within 48 h. Although this conversion is lower than that observed for isolated monolignols (25.0–167 mg/g_substrate_, 10–110% methoxy conversion), the formation of substantial quantities of oxygenated C_1_ compounds from a structurally complex lignin polymer demonstrates that iron-induced demethoxylation is not restricted to small model substrates but can also operate in more realistic lignin-derived materials. The lower conversion likely reflects the heterogeneous three-dimensional structure of lignin, in which steric constraints limit accessibility of methoxy groups and competing oxidation reactions involving other functional groups may occur. Because the mechanism targets the methoxy substituent itself rather than a specific macromolecular structure, it is chemically plausible that similar reactions occur in the diverse pool of lignin-derived organic matter present in soils. This functional-group–based perspective, therefore, provides a mechanistic bridge between the well-defined model systems investigated here and the complex mixtures of lignin fragments and other methoxylated aromatic compounds that characterize natural soil organic matter.

To assess whether the demethoxylation mechanism operates under environmentally relevant conditions, we extended our experiments to a range of natural soils containing iron minerals and lignin-derived organic matter. Following sterilization (drying at 105°C) and subsequent rewetting of the soils under oxic conditions without the addition of external H_2_O_2_ or iron species, clear formation of CH_3_OH and CH_2_O was observed across all investigated samples, spanning a pH range of 3.2–7.9. As a control, sodium azide (NaN_3_), a potent inhibitor of enzymatic activity and microbial metabolism, was added to three representative soil samples. No significant changes in CH_3_OH and CH_2_O production were observed (Fig. S18). This provides additional evidence that the formation of CH_3_OH and CH_2_O is driven by abiotic processes rather than residual enzymatic activity or intracellular metabolites persisting after sterilization^36^. The production of oxygenated C_1_ compounds showed significant correlations with both TOC and methoxy group content, indicating that lignin-derived methoxy groups represent an important precursor pool in soils. Importantly, when methoxy groups were chemically removed using hydroiodic acid treatment (Zeisel demethoxylation)^28^, the formation of CH_3_OH was completely suppressed, and CH_2_O production was strongly reduced. These observations provide direct evidence that methoxy groups in soil organic matter constitute the primary source of the observed oxygenated C_1_ compounds.

The experiments further demonstrate that iron-induced demethoxylation proceeds under oxic abiotic conditions typical of many soils, and may also operate during transient anoxic phases, provided that iron redox cycling and ROS formation occur upon reoxygenation. In soils, the redox cycling of iron minerals and the formation of ROS, including H_2_O_2_ and hydroxyl radicals, are well documented and can be enhanced during environmental perturbations such as wetting–drying cycles^6,27^. These processes create favorable conditions for iron-mediated oxidative reactions capable of transforming lignin-derived functional groups. However, within the experimental framework applied here, it was not possible to directly quantify iron redox transformations, high-valent iron-oxido species, or ROS.

The original model studies on the formation of iron(IV)-oxido species from iron(II) using H_2_O_2_ in aqueous solution involved bispidine ligands and indicated that the reaction proceeds from pH 2 to 6, with formation and decay rates depending on pH^25,36^, and this was confirmed in our model studies under environmentally relevant conditions, leading to an optimized set of parameters used in all our model studies and therefore also used here^10,11,13^. Consistent with this, our soil experiments demonstrate CH_3_OH and CH_2_O formation across a broad pH range (3.2–7.9), supporting the environmental relevance of the proposed mechanism beyond the conditions used in the model systems. Across this range, no significant correlation was observed between pH and the total yield of CH_3_OH and CH_2_O, indicating that overall abiotic C₁ formation is largely independent of soil pH within this interval.

However, controlled pH-adjustment soil experiments revealed systematic shifts in product distribution: CH_3_OH concentrations increased with increasing pH, whereas CH_2_O concentrations generally decreased, except for a slight increase at pH 6.9. These patterns likely reflect pH-dependent iron redox chemistry. At higher pH, Fe³⁺ reduction is favored, while the regeneration of highly reactive iron–oxido species depends on the availability of oxidants such as H_2_O_2_ or other ROS. At the same time, decreasing Fe³⁺ solubility may limit oxidant formation, thereby suppressing further oxidation of CH_3_OH to CH_2_O and promoting CH₃OH accumulation^37^.

Taken together, these observations demonstrate that abiotic iron-driven oxidative processes provide a mechanistically distinct pathway for the transformation of methoxylated organic matter in soils, linking lignin-derived functional groups to the formation of oxygenated C₁ compounds under environmentally relevant conditions. At the same time, experiments with fresh soils and microbial cultures demonstrate that the oxygenated C₁ compounds produced through this abiotic mechanism are rapidly consumed by soil microorganisms^29–32^.

**Fig. 6:**
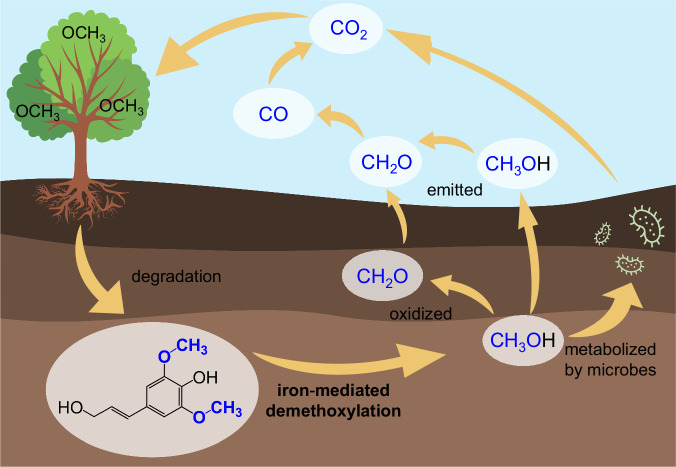
Proposed role of iron-induced demethoxylation in the global C_1_ cycle. Conceptual model: Lignin-derived methoxy groups (–OCH_3_) are cleaved from aromatic structures via an iron(IV)-oxido-induced mechanism in the presence of iron oxides and ROS, resulting in the formation of CH_3_OH and CH_2_O. These oxygenated compounds undergo further oxidation, both abiotically and microbially, ultimately producing CO and CO_2_, which are released into the atmosphere. While the majority of CH_3_OH and CH_2_O is metabolized within soils, a fraction may be released to the atmosphere, where it contributes to local and global C_1_ compound cycling and influences atmospheric chemistry. Tree graphic adapted from a free PNG by Vecteezy (https://www.vecteezy.com/png/19613629-tree-png-graphic-clipart-design); all other graphical elements were created by the authors.

Methylotrophic bacteria efficiently metabolize CH_3_OH and CH_2_O as carbon and energy sources, converting them further into formic acid, CO, and ultimately CO_2_. Consequently, although substantial amounts of CH_3_OH and CH_2_O may be produced abiotically in soils, only a fraction is likely to accumulate or be emitted to the atmosphere. Instead, these compounds primarily act as intermediate substrates, linking abiotic oxidative transformations of lignin-derived organic matter to the microbial C_1_ metabolism in terrestrial ecosystems. The large global reservoir of lignin-derived methoxy groups suggests that iron-induced demethoxylation may represent a previously underappreciated abiotic pathway in terrestrial carbon cycling, as schematically illustrated in Fig. 6. Soils globally store approximately 2400 Gt of organic carbon^38^, of which an estimated 3–4 % consists of methoxy groups derived from lignin and related aromatic compounds^39–41^. This corresponds to a global methoxy pool of roughly 190–250 Gt potentially available for iron-mediated demethoxylation. In our soil incubation experiments conducted under oxic conditions, 0.13–1.36% of soil methoxy groups were converted to CH_3_OH and CH_2_O during a single 48 h wetting–drying cycle, and 6–7 % after ten consecutive cycles, simulating repeated rainfall or flooding events. A conservative first-order extrapolation—assuming only one wetting–drying cycle per year and oxic soil conditions—suggests a potential abiotic production of approximately 0.25–3.4 Gt yr⁻¹ of methoxy-derived CH_3_OH and CH_2_O. This range exceeds current estimates of global atmospheric CH_3_OH emissions (0.075–0.49 Gt yr⁻¹), which are primarily attributed to plant growth and decay^42–45^, indicating that abiotic soil processes may represent a substantial but previously overlooked source of oxygenated C₁ compounds. The contribution of this pathway is therefore expected to be largely confined to oxic or intermittently oxygenated soils, whereas strictly anoxic environments are dominated by microbial C₁ turnover processes.

These estimates should be interpreted as order-of-magnitude approximations intended to place the observed reaction rates into the context of global carbon fluxes. The extrapolation necessarily simplifies the complexity of natural soil systems and does not account for the strong spatial variability in soil composition, iron mineralogy, redox dynamics, and hydrological conditions. Moreover, scaling laboratory incubation experiments to global environments inevitably introduces substantial uncertainties, particularly with respect to the frequency and intensity of wetting–drying events and the availability of ROS. Importantly, the abiotic demethoxylation pathway described here operates under oxic conditions, as demonstrated by the experimental systems used in this study. Consequently, its contribution will depend on the extent to which soils experience oxic or periodically oxygenated conditions, such as those occurring in well-aerated soils, surface horizons, or during wetting–drying cycles. The calculated values should therefore be regarded as indicative of the potential magnitude of this abiotic pathway rather than precise global flux estimates, and they likely represent an upper limit for environments where iron-driven oxidative processes are active.

At the same time, the majority of CH_3_OH and CH_2_O formed through this pathway is unlikely to accumulate in natural soils. Experiments with untreated soils and microbial cultures demonstrate that methylotrophic microorganisms rapidly consume these compounds and convert them further to formate, CO, and ultimately to CO_2_. Consequently, much of the abiotically produced CH_3_OH likely functions as a transient intermediate linking abiotic oxidative transformations of lignin-derived organic matter with microbial C₁ metabolism, rather than being directly emitted to the atmosphere. Temperature-dependent experiments (6–50 °C) further indicate the expected relationship between temperature and abiotic C_1_ compound formation (Fig. S19), suggesting that climate warming may enhance the contribution of iron- and ROS-mediated oxidation of lignin-derived organic matter in soil.

Iron-induced demethoxylation complements previously proposed abiotic pathways for C₁ (and C_2_) compound formation, including oxygen-atom transfer^11,13^ and hydrogen-atom abstraction reactions^12,19^ involving heteroatom-bound methyl groups. In contrast to these mechanisms, the pathway described here enables the direct release of CH_3_OH from aromatic methoxy groups without the formation of methyl radicals, thereby revealing a mechanistically distinct abiotic oxic route for generating oxygenated C_1_ compounds under ambient environmental conditions.

Taken together, these observations highlight the potential importance of iron-mediated oxidative chemistry in the transformation of lignin-derived organic matter under environmentally relevant conditions. By linking lignin demethoxylation, reactive oxygen species formation, microbial C_1_ metabolism, and atmospheric trace-gas fluxes, the pathway identified here highlights how abiotic and biotic processes jointly regulate the cycling of reactive C_1_ compounds in soils and their exchange with the atmosphere.

## Methods

### Chemicals

All of the following chemicals are commercially available and used as purchased: formaldehyde (Roth, Germany, 30%), methanol (Honeywell, Germany, 99,8%), L-ascorbic acid (Sigma Aldrich, USA, 99%), trifluoromethanesulfonic acid (Sigma Aldrich, USA, 99%), hydrogenperoxide (Sigma Aldrich, Germany, 30%), pentafluorophenylhydrazine (Sigma Aldrich, Germany, 98%), sinapyl alcohol (Cayman Chemicals, USA, 95%), coniferyl alcohol (Sigma Aldrich, USA, 98 %), 2-methoxyphenol (Sigma Aldrich, USA, 98%), 3-methoxyphenol (Sigma Aldrich, USA, 96%), 4-methoxyphenol (Sigma Aldrich, France, 99%), 2-methoxy-d_3_-phenol (CDN Isotopes, Canada, 98 %, 99,8% d_3_), oxygen-^18^O_2_ (Sigma Aldrich, USA, 99 %, 97% ^18^O) and Fe_2_O_3_ (Sigma Aldrich, USA, ≥96 %).

The isotopically labeled H_2_^18^O_2_ was obtained from the Costas group at the University of Girona with an isotopic composition of 26 ± 1 % H_2_O_2_ content, 77 ± 1% ^18^O-^18^O, 12 ± 1 % ^18^O-^16^O, 7 ± 1% ^16^O-^16^O. The ^18^O-labeled 2-methoxyphenol was produced by Campro Scientific with a purity of ≥95.0% and an isotopic purity of ≥90.0%. The bispidine ligand and complex were prepared as described in the literature^46,47^.

### Experimental setup

The experiments were conducted in 50 mL crimp vials, each containing 10 μmol of an iron species (either hematite or the bispidine complex). For the standard experiment, 0.05 μmol of triflic acid or ascorbic acid (pH = 2.3) and 25 μmol of substrate were dissolved in 7.5 mL of ultrapure water and then added to the iron species. The pH remained approximately constant throughout the 48 h reactions. The vials were sealed with a mortar cap with a butyl/PTFE septum. To initiate the reaction, 200 μmol of H_2_O_2_, dissolved in 2.5 mL of ultrapure water, was injected through the septum. All experiments were prepared and stored under normal laboratory conditions (22 °C,1013 mbar, and under N_2_/O_2_ atmosphere) for 48 h, in complete darkness. Soil experiments followed the same conditions as the standard experiment, but instead of the chemical substrates, 5 g of sterilized and milled soil was used, mixed in 10 mL of ultrapure water. Prior to starting the experiment, the entire setup was sterilized by heating at 105°C for 1 h. For the wetting–drying cycles, the soils were incubated as described above, except that they were dried for 48 h prior to each wet incubation. Degradation experiments were performed in 500 mL sterile glass bottles with 50 g fresh soil and 100 mL ultrapure water, and stored at 22 °C in darkness. A normal CH_3_OH sampling process was applied. For further details, statistics, and calculations, we refer to^13^.

### GC-(MS)-(FID)-(BID) measurements

A brief description of the measurements is made, for more details, we refer to^13^.

CH_3_OH was measured with a GC-FID (Shimadzu GC-2010 Plus, FID-2010 Plus) equipped with a ZB-WAXplus (Zebron) column (30 m, ⌀ = 0.25 mm, *d*f, 0.25 μm) and an AOC 20i autosampler. CH_2_O was first derivatized with pentafluorophenylhydrazine and then measured with a GCT Premier—TOF Mass spectrometer (Waters Corporation), which was equipped with a DB-5 column (60 m; ⌀ = 0.32; df, 1 μm). To terminate the reaction, 10 µL of catalase was added to the CH_3_OH and CH_2_O samples.

The labeled CH_3_OH and CH_3_Br were measured with a GC-2010 Plus (Shimadzu, Japan) coupled to an MS-QP2020 (Shimadzu, Japan). The instrument was equipped with a ZB-624 column (Phenomenex, USA) that measures 60 m × 0.32 mm × 1.8 μm. A split ratio of 5.0, an injection temperature of 200 °C, and a helium column flow of 1.51 mL min^–1^ were chosen.

The temperature program for CH_3_OH starts at 40 °C (2 min), followed by constant heating at a rate of 50 °C min^–1^ to 150 °C (3 min). Before the injection, the samples (0.5 mL) were equilibrated at 85 °C for 30 min in a 1.5 mL glass vial. For the measurement, 200 μL of the headspace was manually injected using a 1 mL glass syringe (VICI, USA).

The temperature program for CH_3_Br starts at 30 °C (8 min), followed by constant heating at a rate of 30 °C min^–1^ to 180 °C (1 min). The analysis is done in full scan (46–200 AMU) and simultaneously in SIM-mode. For the unlabeled samples, 94 AMU and 96 AMU were used for the detection of CH_3_Br.

Quantification of the OCH_3_ content was performed with a GC-FID (HP 6890 GC; Hewlett Packard, USA) with an HP 6890 autosampler (Hewlett Packard, USA). The GC was equipped with a DB-5 column (25.0 m x 320 μm × 0.52 μm), and 50 μL of the gas sample was injected (200 °C, split 10) for the measurement. The FID ran with hydrogen gas (40 mL min^–1^), synthetic air (400 mL min^–1^), and nitrogen as a makeup gas (45 mL min^-1^). The column temperature was held constant at 150 °C for 6 min. The calibration of the GC instrument was performed by using an internal beech wood standard (HUBG4)^48^ between 6 μg and 200 μg methoxy group content. A linear regression was fitted to the obtained values (*R*^2^ = 1.00), and a daily factor was measured with each measurement.

### NDIR detektion

TOC measurements were performed with a TOC-V_CPH_ coupled to an SSM-5000A (Shimadzu, Japan). The dried soil sample (80 mg) is heated in an oven at 900 °C with a constant supply of oxygen and with the aid of vanadium(V) oxide as a catalyst. All carbon in the sample is oxidized to CO_2_ and transported via O_2_ to a non-dispersive infrared detector. The amount of CO_2_ detected corresponds to the total carbon content in the sample. The TOC is not measured directly, but is calculated by subtracting the inorganic carbon content from the total carbon. To obtain the TIC of the sample, it is acidified in a separate oven of the solid’s module with acid, and the CO_2_ produced by the reaction of acid and carbonate is detected and then subtracted.

### Computational setup

Calculations were performed using the Gaussian 16 suite of programs^49^. Geometry optimizations were carried out using the B3LYP-D3 functional with the LACVP basis set, comprising the LanL2DZ-Los Alamos effective core potential for Fe and a 6-31 g(d) basis set for the other atoms^50–54^. Single-point calculations were performed using the def2-TZVP basis set^55–57^ on the optimized geometries. The solvation energies were computed using the polarizable continuum solvent model (PCM) with water as solvent. Frequency calculations were performed on the optimized structures of each spin state to verify them as minima on the PES and to obtain free energy corrections. Unless otherwise mentioned, all reported energies are B3LYP-D3 solvation energies, incorporating free energy corrections at 298.15 K. The inclusion of solvent in the optimization has been shown to lead to very similar structures and identical trends for the energetics^13,58–60^. Therefore, here, we have only performed single-point calculations for solvation, using the PCM model.

### Statistical information

Unless indicated otherwise, all experiments were performed with *n*  =  3 replicates, and the error bars were calculated with Excel version 2108. To test for significant differences in pH values and CH_3_OH and CH_2_O formation, single-factor analysis (one-tailed Pearson correlation) was used with the degree of freedom of n-2.

## Supplementary information

Supplementary Information

Peer Review file

## Data Availability

The experimental data supporting the findings of this study are available from heiDATA, the institutional research data repository of Heidelberg University (10.11588/DATA/PCFAAH).
