# Supplementary material for: Iron-induced demethoxylation of lignin as an important source for methanol and its oxidation products in the environment
Source: Nat Commun. 2026 Aug 13;17:8334. doi: 10.1038/s41467-026-76679-x (PMC13473683; doi:10.1038/s41467-026-76679-x)
Supplement: Supplementary file 1 [file 41467_2026_76679_MOESM1_ESM.pdf]

Supplementary Information

Iron-induced demethoxylation of lignin as an important source for methanol and its oxidation products in the environment

Jonas Hädeler[1*#], Gunasekaran Velmurugan[2,3*#], Rebekka Lauer[1], Isabel Hanstein[1], Julia Wenhuda[1], Rejith Radhamani[2], Peter Comba[2,4*] and Frank Keppler[1,5*]

[1] Institute of Earth Sciences, Heidelberg University, INF 234-236, D-69120 Heidelberg, Germany

[2] Anorganisch-Chemisches Institut, Heidelberg University, INF 270, D-69120 Heidelberg, Germany

[3]Department of Chemistry, National Institute of Technology – Tiruchirappalli, 620015, Tamil Nadu, India

[4] Universität Heidelberg, Interdisciplinary Center for Scientific Computing, INF 205, D-69120 Heidelberg, Germany

[5] Heidelberg Center for the Environment (HCE), Heidelberg University, D-69120 Heidelberg, Germany

[#] These authors contributed equally

*Correspondence:

Jonas Hädeler: jonas.haedeler@geow.uni-heidelberg.de

Gunasekaran Velmurugan: velmurugan@nitt.edu

Peter Comba: peter.comba@aci.uni-heidelberg.de

Frank Keppler: frank.keppler@geow.uni-heidelberg.de

Table of contents

Title	Page No.
Fig. S1. Abiotic formation of CH_3OH and CH_2O from monolignols and methoxyphenols using $Fe^{II/III}$ species and H_2O_2	4
Fig. S2: Conversion rates of 2-methoxyphenol to CH_3OH and CH_2O with different setups of the added iron species, acid, and H_2O_2	5
Fig. S3: Conversion rates of phenol to CH_3OH and CH_2O with different iron species, triflic acid, and H_2O_2	6
Fig. S4: Conversion rates of sinapyl alcohol to CH_3OH and CH_2O with $[(L)Fe^{II}Cl_2]$ or Fe_2O_3, triflic acid or ascorbic acid, and H_2O_2	7
Fig. S5: Mass track of CH_3OH in standard experiments with d_3-2-methoxyphenol	8
Fig. S6: Measurements of CH_3OH in standard experiments with $^{18}OCH_3$-2-methoxyphenol	9
Fig. S7: Measurements of CH_3OH in standard experiments with $H_2{}^{18}O_2$ instead of H_2O_2 in the headspace and in H_2O.	10
Fig. S8: Measurements of CH_3OH in standard experiments with $^{18}O_2$ instead of atmospheric O_2 in the headspace and in H_2O.	11
Fig. S9: Mass track of CH_2O in standard experiments with d_3-2-methoxyphenol	12
Fig. S10: Chromatogram of CH_3Br in alkyl radical trapping experiments with CCl_3Br as a scavenger	13
Fig. S11: Conversion rates of lignin to CH_3OH and CH_2O with Fe_2O_3, triflic acid and H_2O_2	14
Table S1: Soil samples from this study and[1]. TOC and methoxy content in w%. pH value was directly measured in the experiment after 48 h of incubation. Total iron content measured in [w%].	15
Fig. S12: Correlations between soil TOC and methoxy content, as well as between TOC or methoxy content with CH_3OH + CH_2O production in sterile soils.	16
Fig. S13: Chromatogram of labeled CH_3OH in soil experiments with added ^2H and ^{18}O labelled 2-methoxyphenol	17-18
Fig. S14: CH_2O measurements in wet-dry-cycles with soil samples CH and GL1	18
Fig. S15: CH_3OH measurements in wet-dry-cycles with soil samples CH and GL1	19
Fig. S16: Degradation of CH_3OH in fresh soils (GL1, CH, and GL2) due to microorganisms.	20
Fig. S17: Degradation of abiotic-produced CH_3OH in GL1 due to added methylotrophic bacteria	21
Figure S18. Formation of CH_3OH and CH_2O from soils BE1, GL1 and CH1 with and without addition of NaN_3	22

Fig. S19: Measurement of CH_3OH and CH_2O in soil GL2 as a function of temperature.	23
Fig. S20: Reproduction of Figure 1	24
Fig. S21: Computed reaction profile of the HAA mechanism of sinapyl alcohol.	25
Fig. S22: Computed reaction profile of the OAT mechanism of sinapyl alcohol.	25
Fig. S23: Optimized geometries (a-c) and spin density plots (d-e) for $[(L)(OH_2)Fe^{IV}{=}O]^{2+}$.	26
Fig. S24: Optimized geometries (a-c) and spin density plots (d-e) of HAA mechanism for $^5TS1_{HS}$, $^5INT1_{HS}$ and $^5TS2_{HS}$.	26
Fig. S25: Optimized geometries (a-c) and spin density plots (d-e) of HAA mechanism for $^3P_{IS}$, and $^5P_{HS}$.	27
Fig. S26: Optimized geometries and spin density plots of demethoxylation mechanism for $^3TS1_{HS}$ (a, c), $^3INT1_{HS}$ (b, d), $^3TS2_{HS}$ (e, g) and $^3TS2'_{HS}$ (f, h).	28
Fig. S27: Computed potential energy surface of $[(L)(OH_2)Fe^{IV}{=}O]^{2+}$ forming a proton-coupled electron transfer reactant complex.	29
Fig. S28: Optimized geometries (a-b) and spin density plots (c-d) of OSET mechanism for ^{3}R-HOTf and ^{5}R-HOTf.	30
Fig. S29: Optimized geometries (a-d) and spin density plots (e-h) of OSET mechanism for ^{3}RC, ^{5}RC, ^{3}RC' and ^{5}RC'.	30
References	31

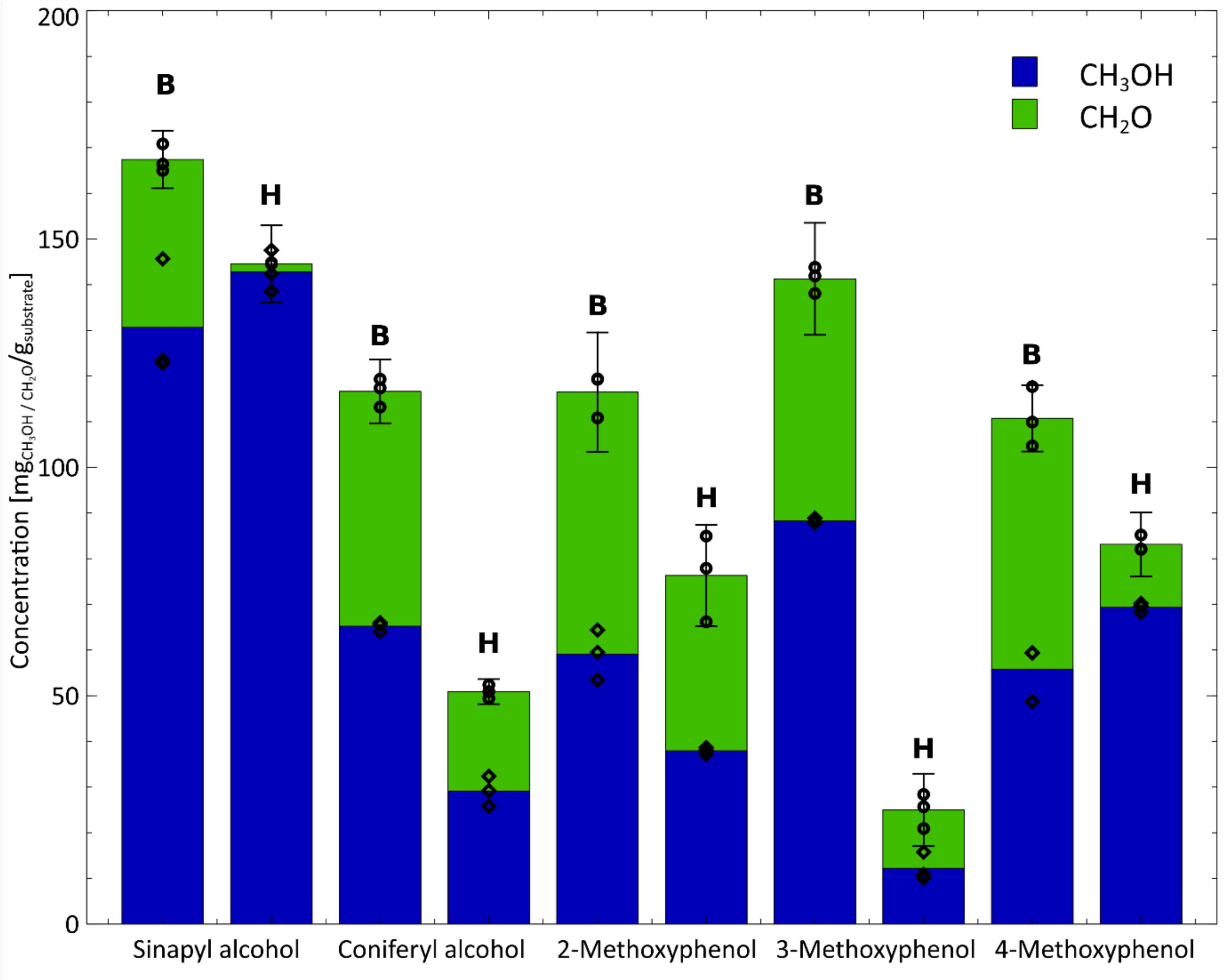

Figure S1. Abiotic formation of CH_3OH and CH_2O from monolignols and methoxyphenols using $Fe^{II/III}$ species and H_2O_2

Formation of CH_3OH and CH_2O from monolignols and methoxyphenols in experiments mediated by 10 µmol [(L)$Fe^{II}Cl_2$], (bar **B**) or Fe_2O_3, (bar **H**). Reactions were performed in 10 mL H_2O, including 25 µmol substrate, 200 µmol H_2O_2, and 0.05 µmol HOtf acid. All provided data were evaluated at 22 °C, ambient pressure (1013 mbar), and under N_2/O_2 atmosphere after 48 h reaction time. Error bars represent the standard deviation (SD) of total CH_3OH and CH_2O formation, based on the mean of three independent experiments (n=3), each measured in triplicate. Diamonds (CH_3OH) and circles (CH_2O) indicate the mean value of each independent experiment.

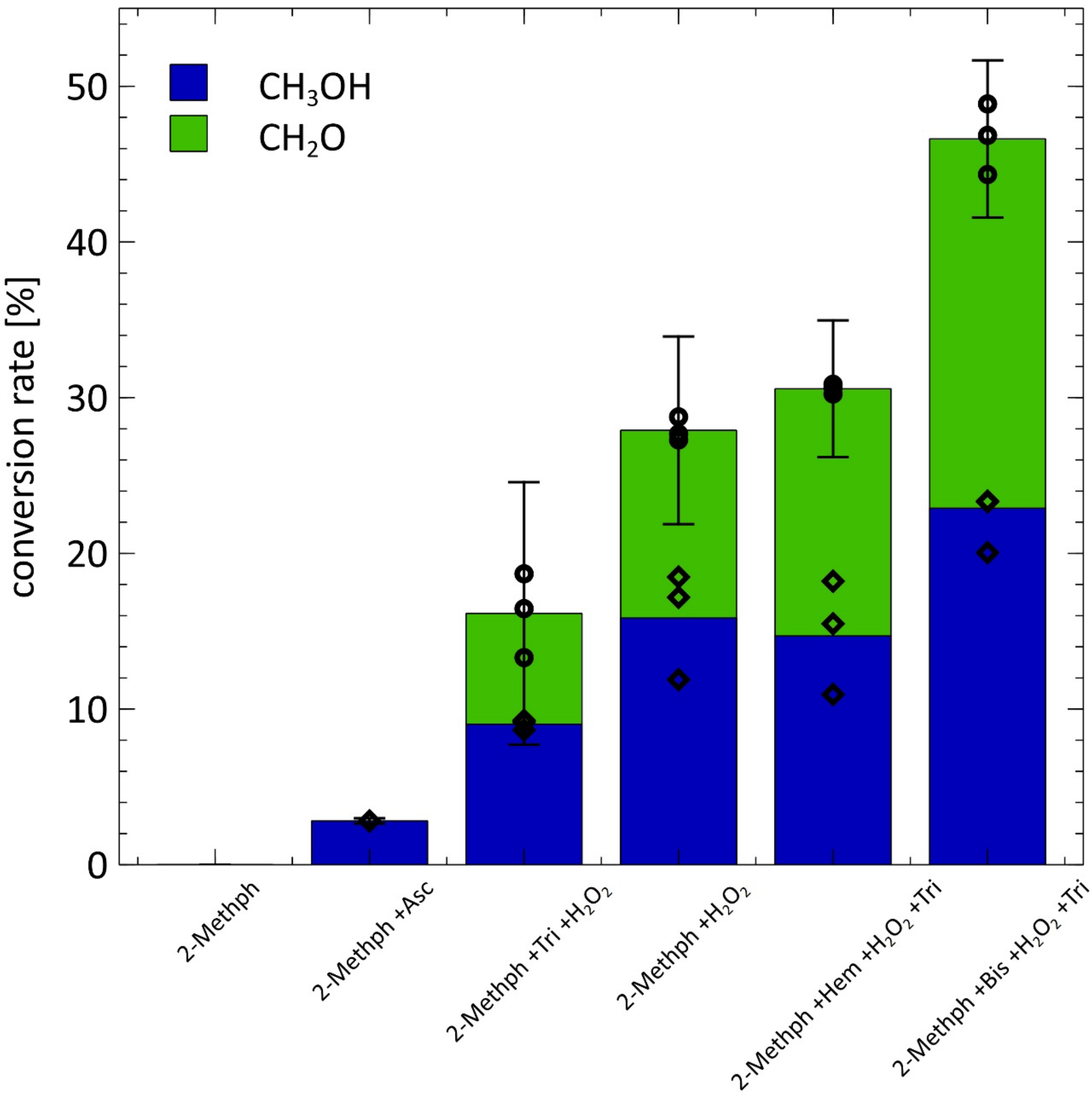

Figure S2. Conversion rates of 2-methoxyphenol to CH_3OH and CH_2O with different setups of the added iron species, acid, and H_2O_2

Formation of CH_3OH and CH_2O from 2-methoxyphenol in experiments by removing parts of the reactants (10 µmol $[(L)Fe^{II}Cl_2]$, or Fe_2O_3, 200 µmol H_2O_2, 100 µmol ascorbic acid, and 0.05 µmol triflic acid). Reactions were performed in 10 mL H_2O. All provided data were evaluated at 22 °C, ambient pressure (1013 mbar), and under N_2/O_2 atmosphere after 48 h reaction time. Conversion rates were related to one methoxy group. Error bars represent the standard deviation (SD) of total CH_3OH and CH_2O conversion, based on the mean of three independent experiments (n=3), each measured in triplicate. Diamonds (CH_3OH) and circles (CH_2O) indicate the mean value of each independent experiment.

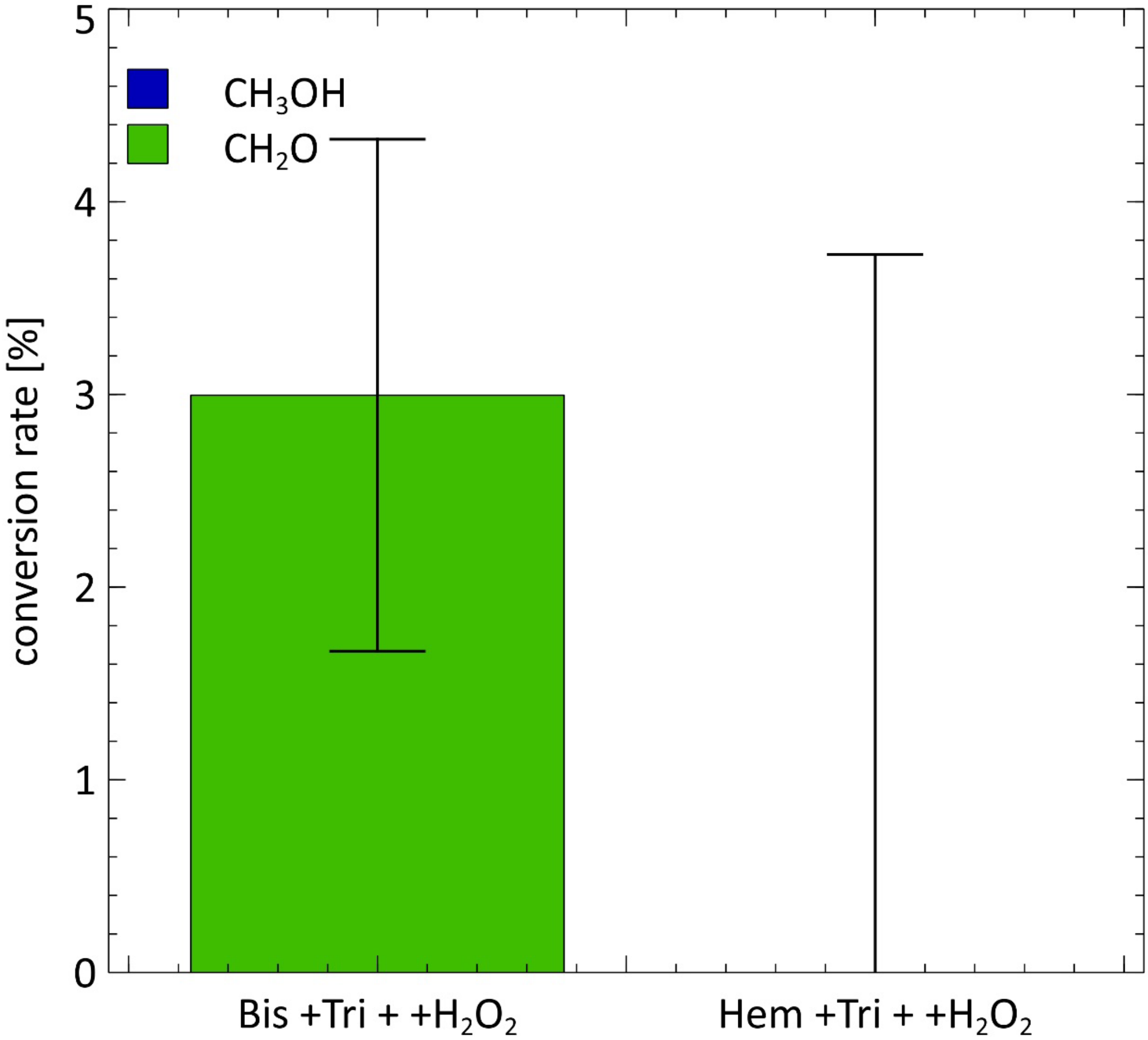

Figure S3. Conversion rates of phenol to CH_3OH and CH_2O with different iron species, triflic acid, and H_2O_2

Formation of CH_3OH and CH_2O from phenol in experiments mediated 10 µmol [(L)$Fe^{II}Cl_2$], or Fe_2O_3 with 200 µmol H_2O_2, and 0.05 µmol triflic acid. Reactions were performed in 10 mL H_2O. All provided data were evaluated at 22 °C, ambient pressure (1013 mbar), and under N_2/O_2 atmosphere after 48 h reaction time. Conversion rates were related to one hypothetical methoxy group. Error bars represent the SD of total CH_3OH and CH_2O conversion, based on the mean of three independent experiments (n=3), each measured in triplicate. Diamonds (CH_3OH) and circles (CH_2O) indicate the mean value of each independent experiment.

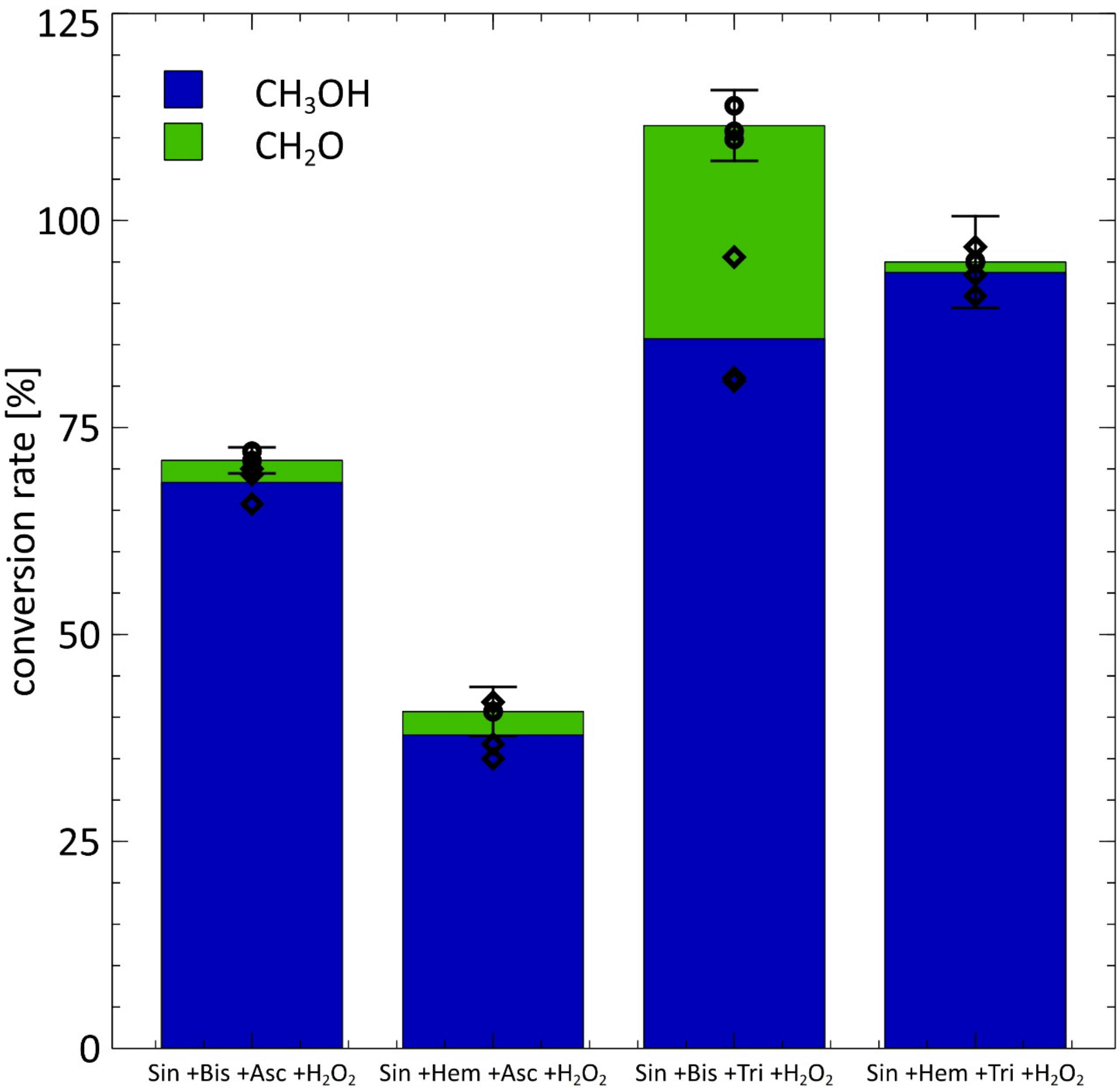

Figure S4. Conversion rates of sinapyl alcohol to CH_3OH and CH_2O with [(L)$Fe^{II}Cl_2$] or Fe_2O_3, triflic acid or ascorbic acid, and H_2O_2

Formation of CH_3OH and CH_2O from sinapyl alcohol in experiments mediated by 10 µmol [(L)$Fe^{II}Cl_2$], or Fe_2O_3 with 200 µmol H_2O_2, and 0.05 µmol triflic acid or 100 µmol ascorbic acid. Reactions were performed in 10 mL H_2O. All provided data were evaluated at 22 °C, ambient pressure (1013 mbar), and under N_2/O_2 atmosphere after 48 h reaction time. Conversion rates were related to one methoxy group. Error bars represent the SD of total CH_3OH and CH_2O conversion, based on the mean of three independent experiments (n=3), each measured in triplicate. Diamonds (CH_3OH) and circles (CH_2O) indicate the mean value of each independent experiment.

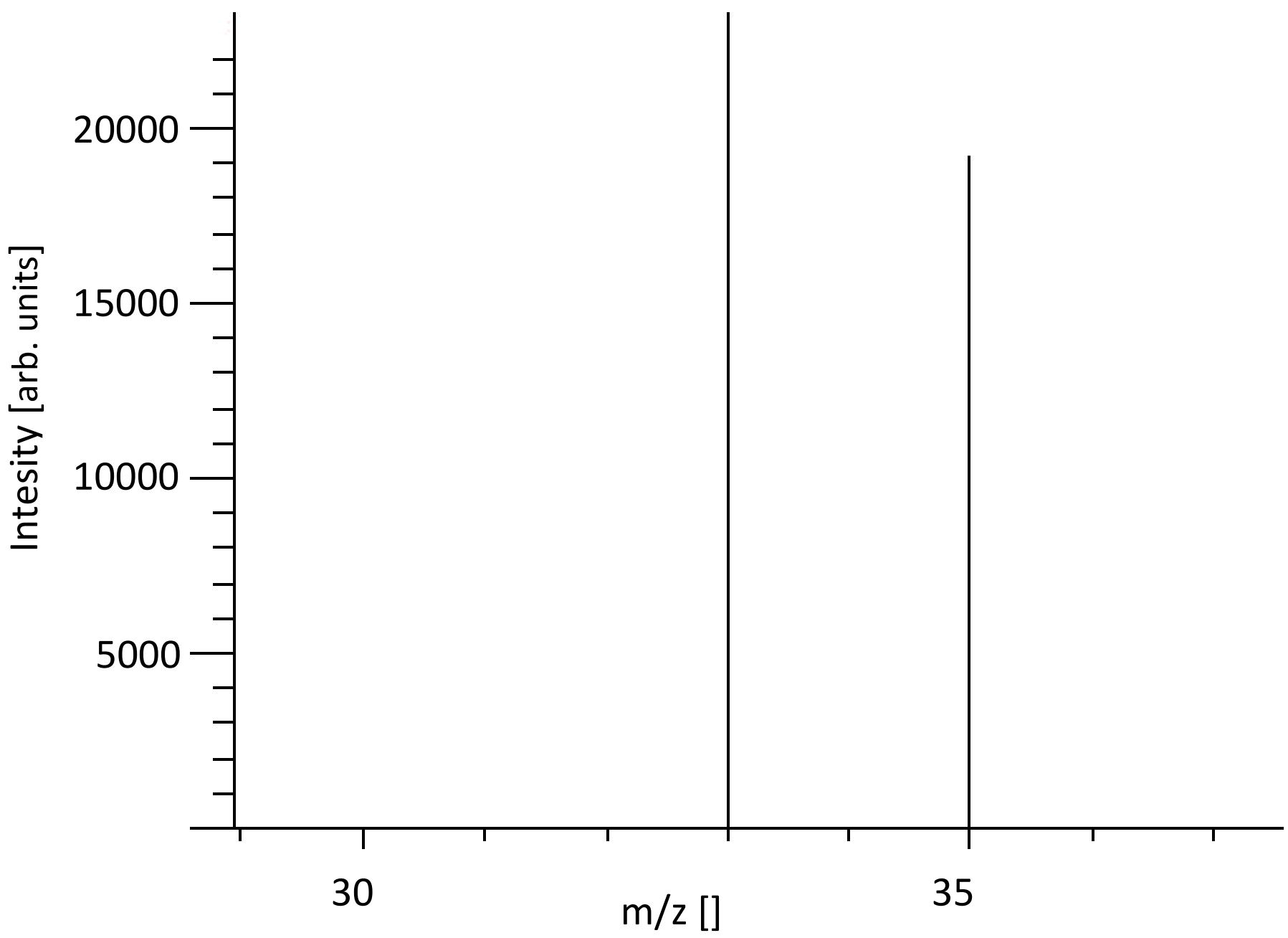

Figure S5. Mass track of CH_3OH in standard experiments with d_3-2-methoxyphenol

The mass tracking of labeled CH_3OH from experiments involving d_3-2-methoxyphenol was measured in the headspace. This comprised using 25 µmol of d_3-2-methoxyphenol, along with 200 µmol of H_2O_2, 10 µmol of $[(L)Fe^{II}Cl_2]$, and 0.05 µmol of triflic acid (at pH=2.3), all dissolved in 10 mL of H_2O. Characteristic masses of 33 and 35 were observed for the labeled CH_3OH. The detection of mass 35 indicates that all three deuterium (^2H) atoms from the d_3-2-methoxyphenol are present in the labeled CH_3OH.

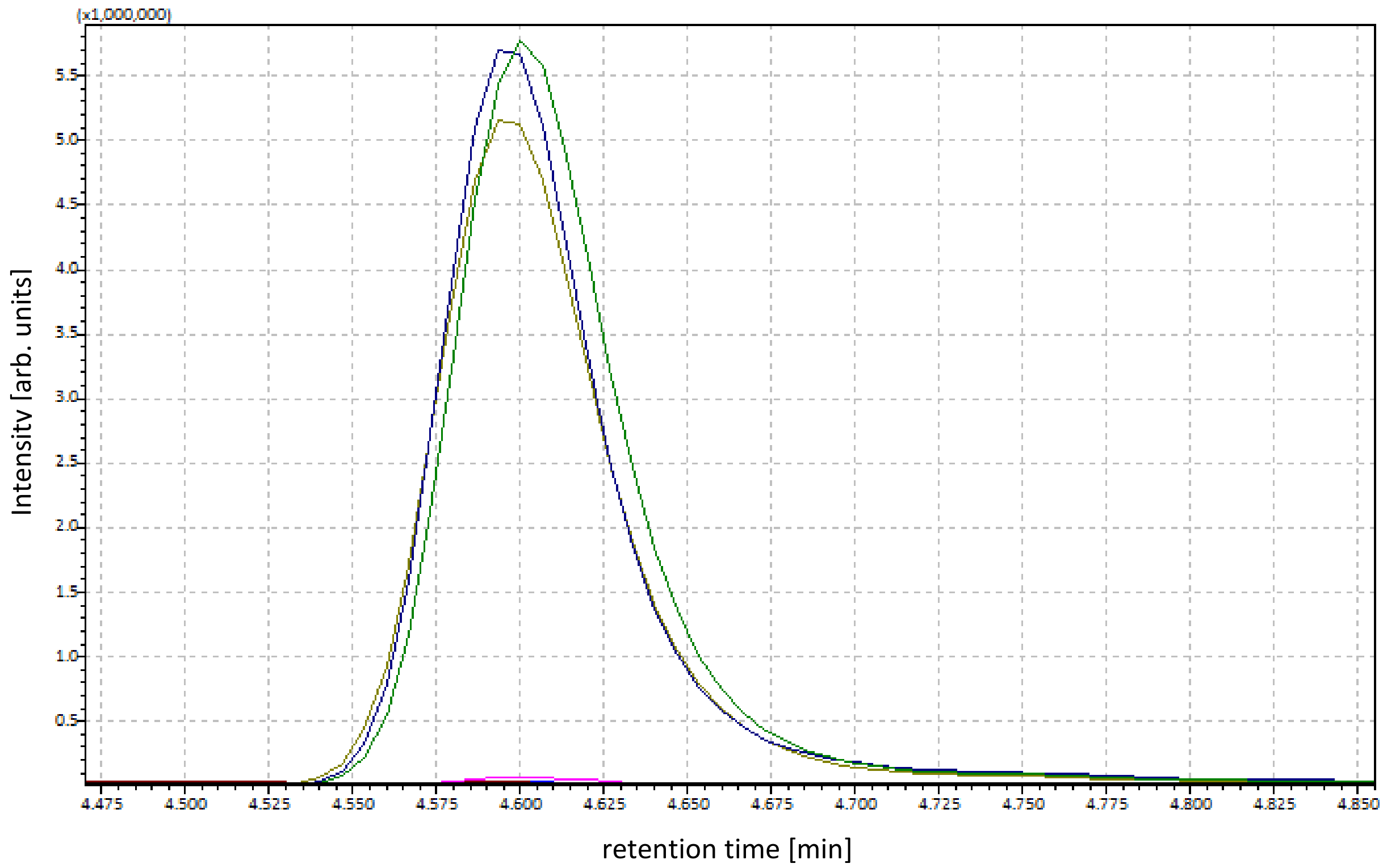

Figure S6. Measurements of CH_3OH in standard experiments with $^{18}OCH_3$-2-methoxyphenol

Experiment with 2 µmol $^{18}OCH_3$ 2-methoxyphenol, 20 µmol H_2O_2, 1 µmol $[(L)Fe^{II}Cl_2]$, and 0.005 µmol triflic acid (pH=2.3) dissolved in 1 mL H_2O. Pink, blue, and brown chromatograms are the measured masses of 29, 30, and 31 of the produced CH_3OH (small to no peaks) in experiments with $^{18}OCH_3$ 2-methoxyphenol. The black line is the blank without substrate (no peak), and green, dark blue, and olive are the masses 33 and 34 of CH_3OH (large peaks) measured in the same experiments.

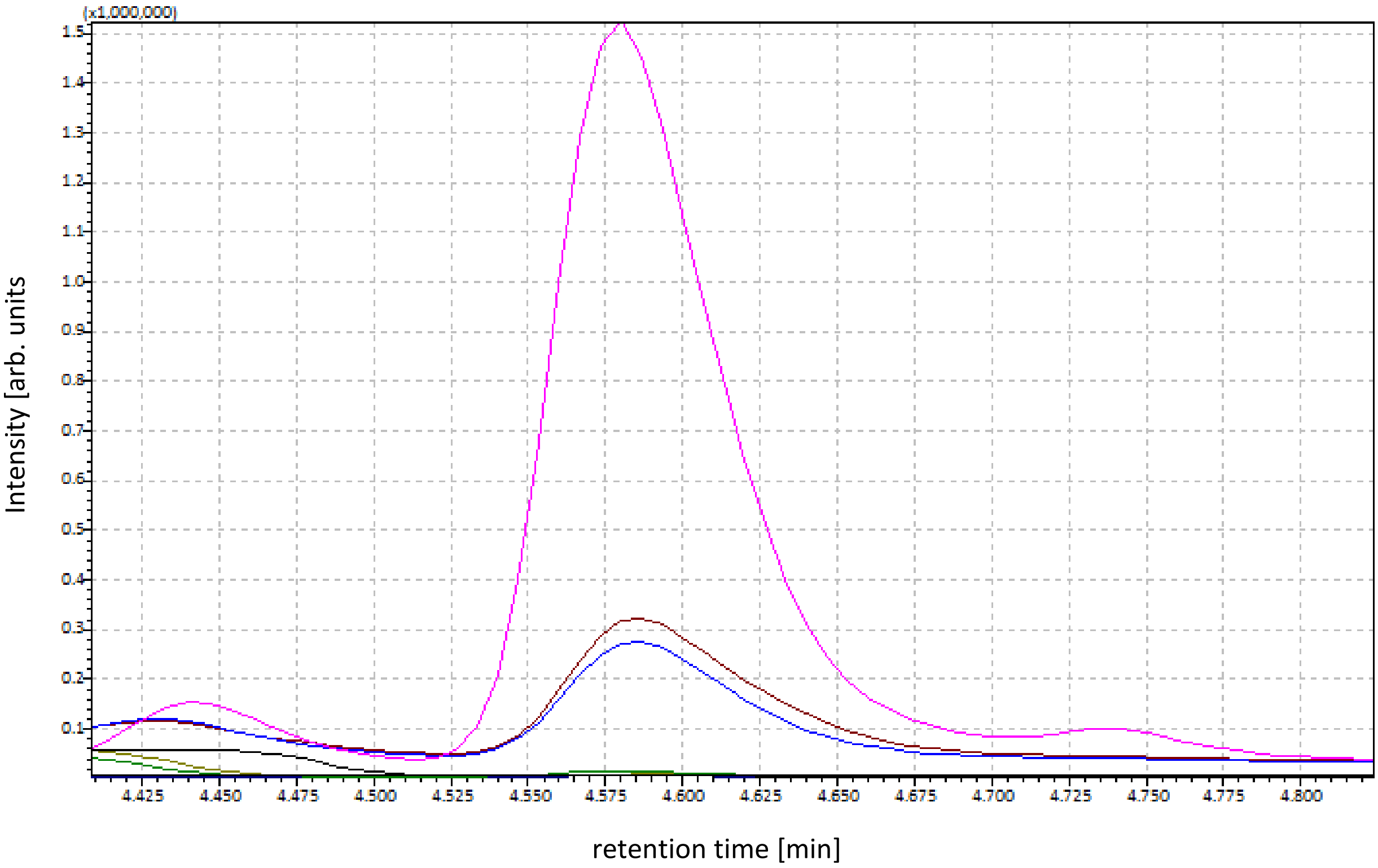

Figure S7. Measurements of CH_3OH in standard experiments with $H_2{}^{18}O_2$ instead of H_2O_2 in the headspace and in H_2O.

Experiment with 25 µmol substrate (sinapyl alcohol, coniferyl alcohol, or 2-methoxyphenol), 200 µmol $H_2{}^{18}O_2$, 10 µmol $[(L)Fe^{II}Cl_2]$, and 0.05 µmol triflic acid (pH=2.3) dissolved in 10 mL H_2O. Pink, blue, and brown chromatograms are the measured masses of 29,30, and 31 of the produced CH_3OH (large peaks) in experiments with sinapyl alcohol, coniferyl alcohol, and guaiacol. The black line is the blank without substrate (no peak), and green, dark blue, and olive are the masses 33 and 34 of CH_3OH measured in the same experiments (small to no peaks).

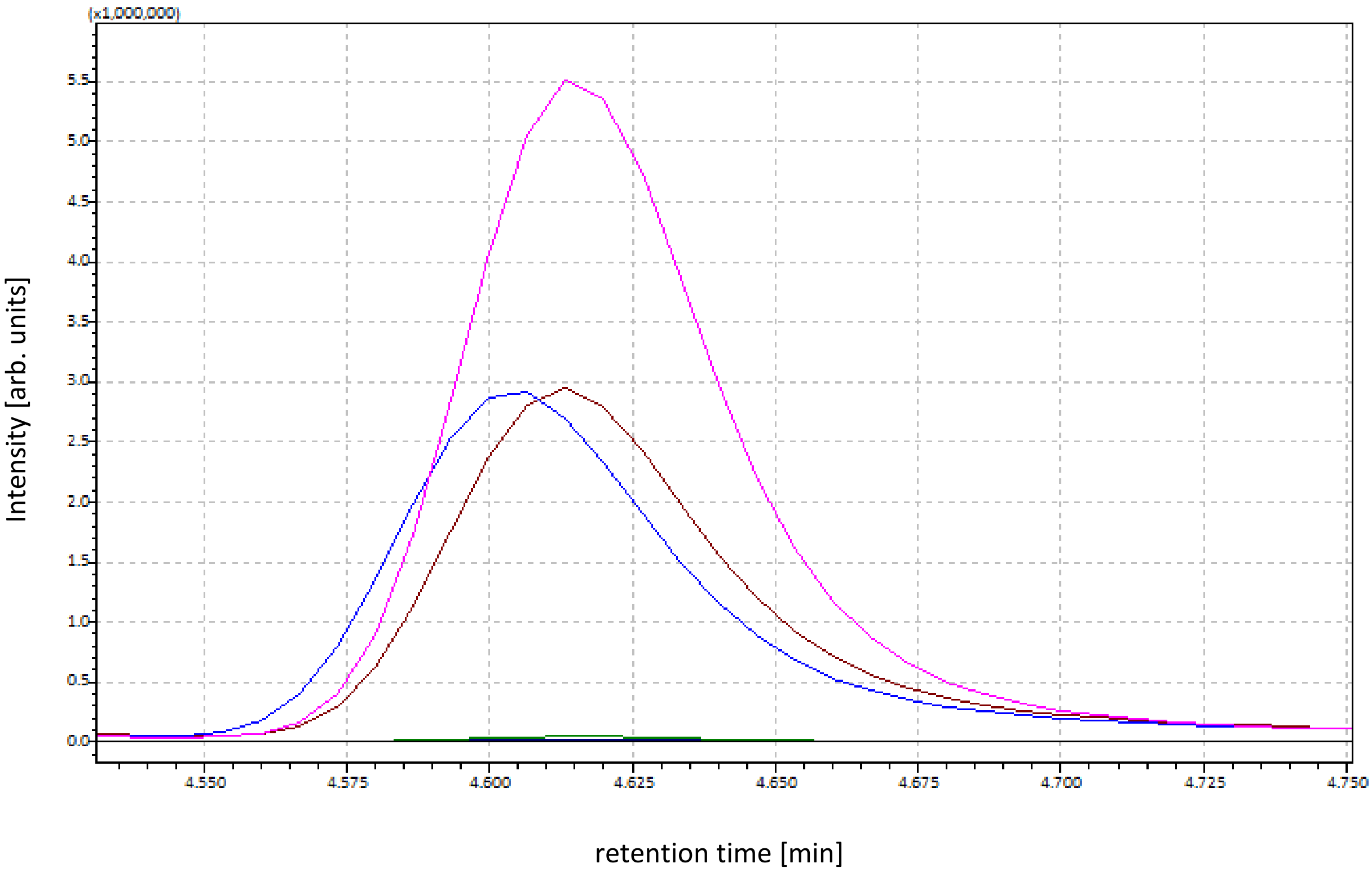

Figure S8. Measurements of CH_3OH in standard experiments with $^{18}O_2$ instead of atmospheric O_2 in the headspace and in H_2O.

Experiment with 25 µmol substrate (sinapyl alcohol, coniferyl alcohol, or 2-methoxyphenol), 200 µmol H_2O_2, 10 µmol $[(L)Fe^{II}Cl_2]$, and 0.05 µmol triflic acid (pH=2.3) dissolved in 10 mL H_2O. Pink, blue, and brown chromatograms are the measured masses of 29,30, and 31 of the produced CH_3OH (large peaks) in experiments with sinapyl alcohol, coniferyl alcohol, and guaiacol. The black line is the blank without substrate (no peak), and green, dark blue, and olive are the masses 33 and 34 of CH_3OH (small to no peaks) measured in the same experiments.

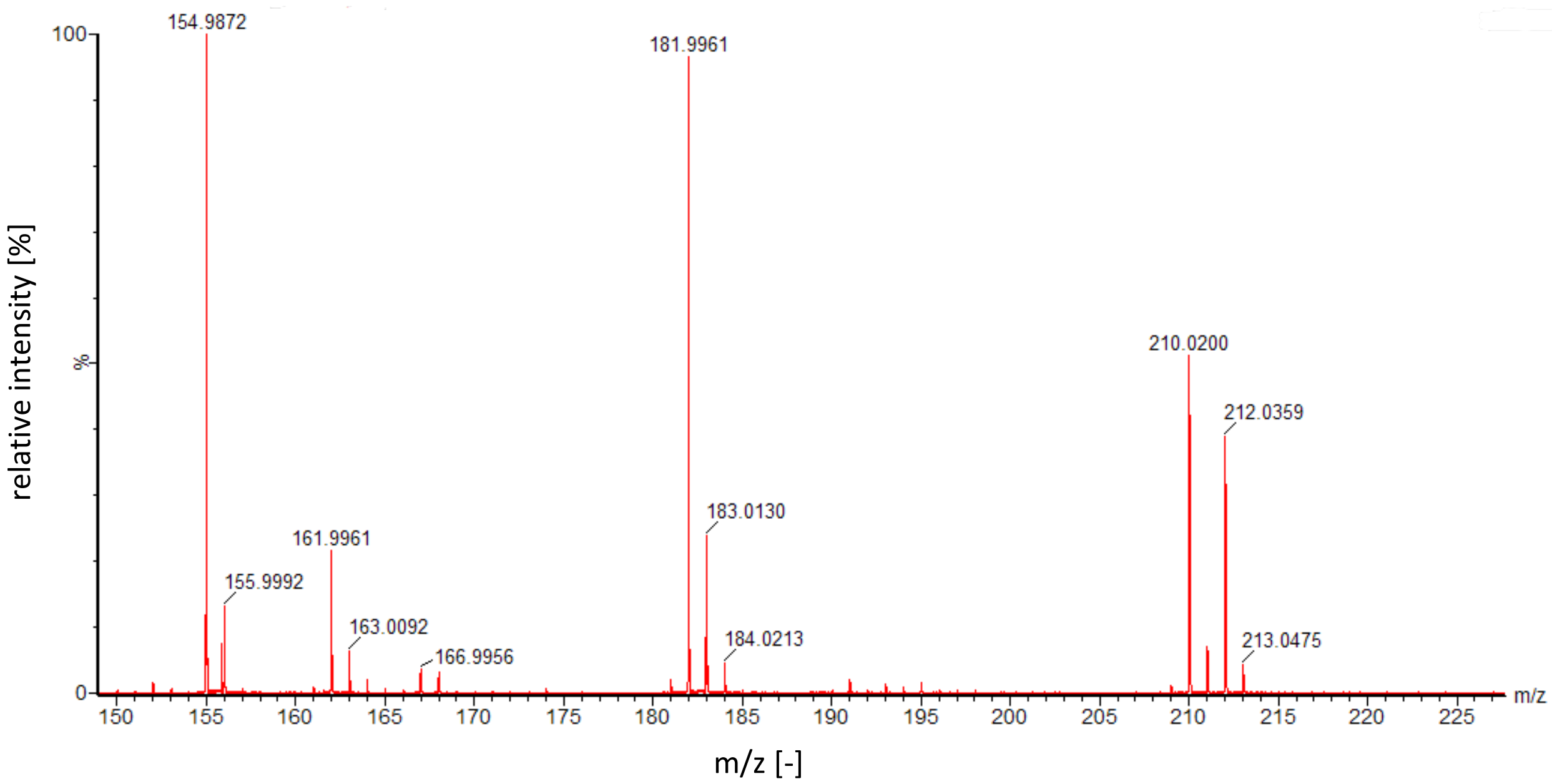

Figure S9. Mass track of CH_2O in standard experiments with d_3-2-methoxyphenol

The mass tracking of labeled CH_2O from experiments with d3-2-methoxyphenol was conducted using a standard protocol. This involved utilizing 25 µmol of d_3-2-methoxyphenol, along with 200 µmol of H_2O_2, 10 µmol of $[(L)Fe^{II}Cl_2]$, and 0.05 µmol of triflic acid (at pH=2.3), all dissolved in 10 mL of H_2O. Characteristic masses of 155, 182, and 210 were observed for the derivatized CH_2O. Additionally, the mass of 212 was identified for the CD_2O, which originated from d3-2-methoxyphenol.

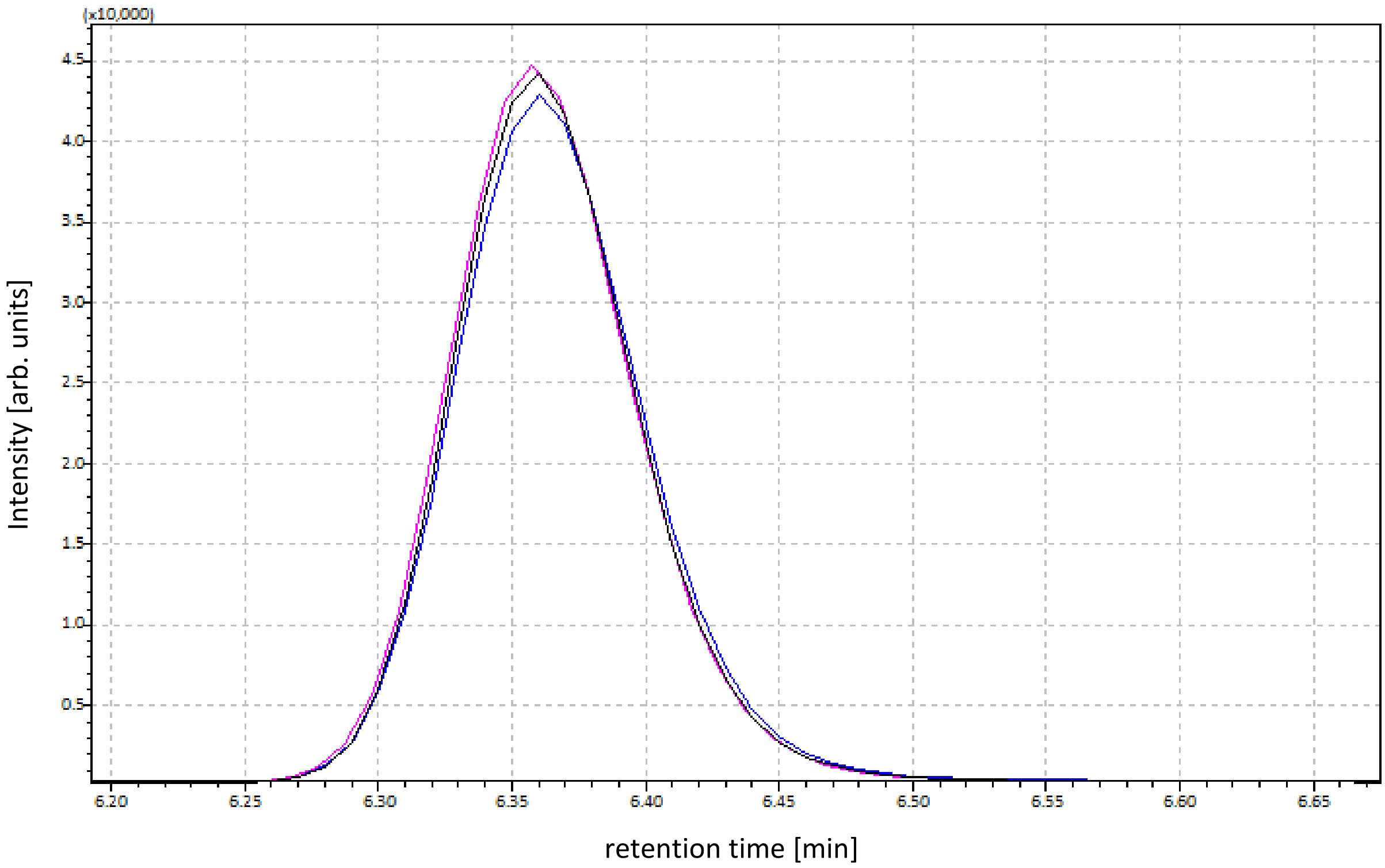

Figure S10. Chromatogram of CH_3Br in alkyl radical trapping experiments with CCl_3Br as a scavenger

Measurement of mass 94 (stable isotope 79 of bromine) and mass 96 (stable isotope 81 of bromine) in experiments with sinapyl alcohol (pink) or 2-methoxyphenol (blue) (25 µmol substrate, 200 µmol of H_2O_2, 10 µmol of $[(L)Fe^{II}Cl_2]$, and 0.05 µmol of triflic acid (at pH=2.3), all dissolved in 10 mL of H_2O). The black line is the blank without a substrate. No differences were measured between blank experiments and experiments with sinapyl alcohol and 2-methoxyphenol.

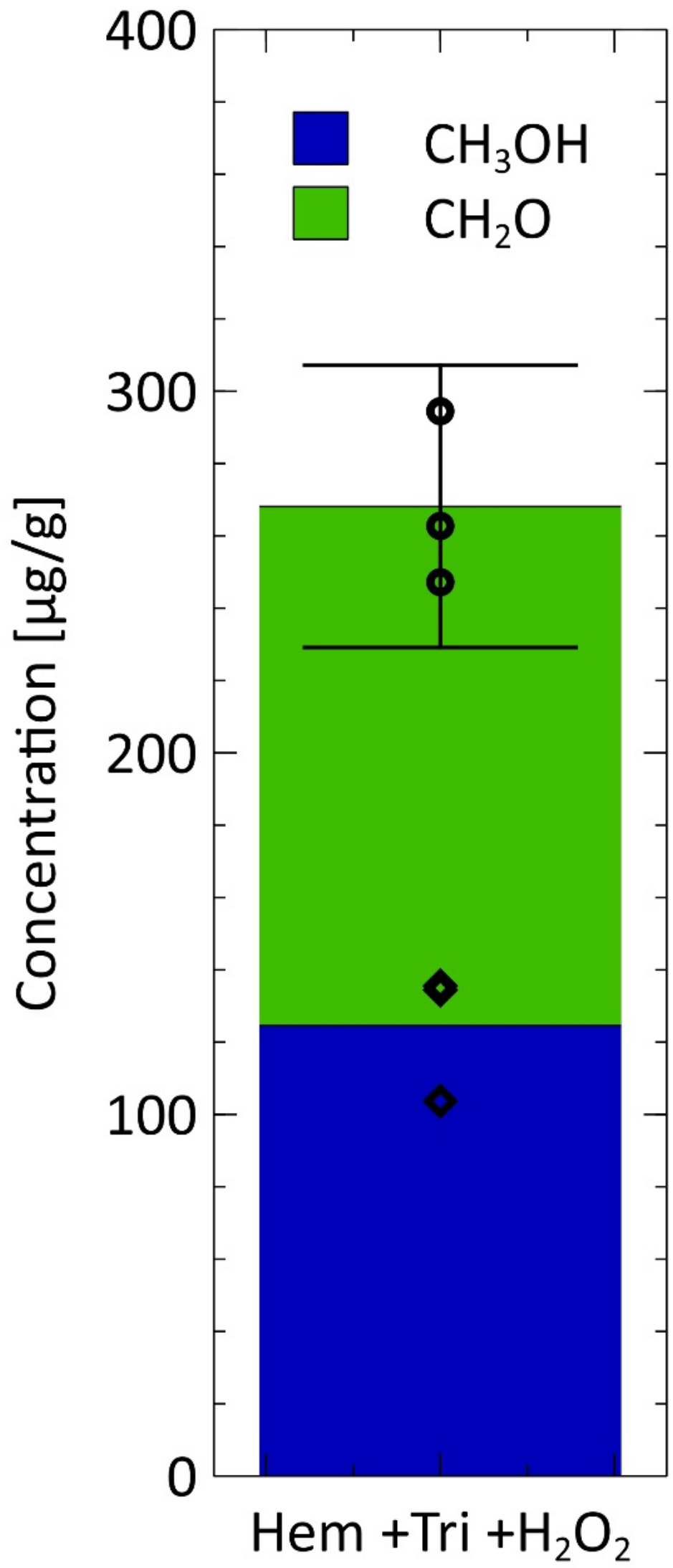

Figure S11. Conversion of lignin to CH_3OH and CH_2O with Fe_2O_3, triflic acid and H_2O_2

Formation of CH_3OH and CH_2O from Lignin (50 mg) in experiments mediated by 10 µmol Fe_2O_3 with 200 µmol H_2O_2, and 0.05 µmol triflic acid. Reactions were performed in 10 mL H_2O. All provided data were evaluated at 22 °C, ambient pressure (1013 mbar), and under N_2/O_2 atmosphere after 48 h reaction time. Conversion rates were related to one methoxy group. Error bars represent the SD of total CH_3OH and CH_2O conversion, based on the mean of three independent experiments (n=3), each measured in triplicate. Diamonds (CH_3OH) and circles (CH_2O) indicate the mean value of each independent experiment.

Table S2. Overview of the soil samples used in this study, including samples from a previous study [1]. The samples were collected near Heidelberg, in the Rhine Basin and the Odenwald region of Germany. Total organic carbon (TOC, slightly negative TOC values are analytical artifacts caused by measurement uncertainties in the calculation of TOC as TC − TIC.) and methoxy group contents are given in weight percent (w%). The pH values were measured directly after 48 h of incubation. Total iron content is reported in weight percent (w%). Data marked with * are taken from a previous study[1].

Soil sample Depth Acronym	Soil type	Lat/Lon	TOC [w%]	Methoxy content [w%]	pH	Total iron content [w%]	CH_3OH/ CH_2O [$\mu g/g_{soil,dw}$]
Hockenheim 10-20 cm (GL1)	Gleysol	49°19'12.8"N/ 8°31'26.6"E	7.83	0.170	7.42	2.64	14.0 / 15.3
Walldorf 40-90 cm (GL4)	Gleysol	49°17'31.3"N/ 8°37'48.7"E	0.07	0.004	5.20	0.56	1.11 / 0.40
Walldorf 10-40 cm (GL3)	Gleysol	49°17'31.3"N/ 8°37'48.7"E	0.79	0.023	4.00	0.44	0 / 5.15
Walldorf forest 0-10 cm (GL2)	Gleysol	49°17'31.3"N/ 8°37'48.7"E	16.2	0.423	3.50	0.40	9.20 / 18.4
Peterstal 10-40 cm (PO2)	Podzolic brown earth	49°27'13.4"N/ 8°45'43.3"E	2.15	0.030	3.70	0.94	3.58 / 14.5
Peterstal 0-10 cm (PO1)	Podzolic brown earth	49°27'13.4"N/ 8°45'43.3"E	8.07	0.139	3.20	0.75	6.88 / 21.4
Unterhof 20-100 cm (CH2)	Chernozem-Para brown earth	49°18'14.3"N/ 8°47'17.9"E	0.59	0.014	7.90	2.80	2.15 / 1.28
Unterhof 0-10 cm (CH1)	Chernozem-Para brown earth	49°18'14.3"N/ 8°47'17.9"E	3.63	0.103	7.40	1.75	3.58 / 14.4
ST. Leon-Rot 10-40 cm (PE2)	Pelosol brown earth	49°13'58.0"N/ 8°40'10.3"E	5.64	0.052	7.60	2.47	1.31 / 7.65
ST. Leon-Rot 0-10 cm (PE1)	Pelosol brown earth	49°13'58.0"N/ 8°40'10.3"E	2.80	0.085	7.80	2.28	6.42 / 14.3
Wilhelmsfeld 0-10 cm (BE1)	Brown earth	49°27'34.8"N/ 8°45'17.4"E	4.87	0095	3.70	0.65	1.96 / 5.82
Birkenheide* 10-25 cm	Pararendzina	49°28'45.1"N/ 8°16'13.1"E	0.14	0.012	4.71	0.16	1.26 / 4.19
Birkenheide* 25-35 cm	Pararendzina	49°28'45.1"N/ 8°16'13.1"E	-0.13	0.005	5.70	0.15	0.97 / 2.60
Malchenberg* 5-15 cm	Pelosol brown earth	49°15'35.6"N/ 8°40'25.7"E	2.12	0.065	7.24	0.40	6.96 / 8.79
Walldorf* 20-35 cm	Podzol brown earth	49°18'05.1"N/ 8°36'31.0"E	-0.58	0.009	5.22	0.37	0.76 / 3.20
Walldorf* 5-15 cm	Podzol brown earth	49°18'05.1"N/ 8°36'31.0"E	1.23	0.036	4.21	0.36	1.39 / 6.54
Walldorf Topsoil*	Podzol brown earth	49°18'05.1"N/ 8°36'31.0"E	3.55	0.129	4.14	0.97	3.91 / 8.45
Schriesheim* 1 0-5 cm	Brown earth	49°28'37.2"N/ 8°42'33.7"E	3.75	0.076	3.62	1.74	5.14 / 12.8
Schriesheim* 2 Topsoil	Chernozem-Para brown earth	49°28'52.2"N/ 8°39'49.7"E	1.06	0.033	7.47	1.85	2.62 / 4.94
Schriesheim* 2 45-55 cm	Chernozem-Para brown earth	49°28'52.2"N/ 8°39'49.7"E	0.93	0.023	7.63	0.27	2.73 / 4.68
Maxdorf* Topsoil	Chernozem	49°28'29.1"N/ 8°16'51.0"E	4.35	0.123	3.87	0.26	2.99 / 8.19
Maxdorf* 5-15 cm	Chernozem	49°28'29.1"N/ 8°16'51.0"E	4.08	0.143	3.96	0.24	3.18 / 8.23
Maxdorf* 15-30 cm	Chernozem	49°28'29.1"N/ 8°16'51.0"E	-0.61	0.011	5.30	1.58	0.98 / 3.18
Altlußheim* ~20 cm	Low moor soil	49°18'38.4"N/ 8°30'28.2"E	11.2	0.188	7.11	4.13	14.7 / 8.95
Hockenheim* ~25 cm	Low moor soil - Podzol brown earth	49°19'12.8"N/ 8°31'26.6"E	6.19	0.197	7.60	3.37	18.0 / 6.52

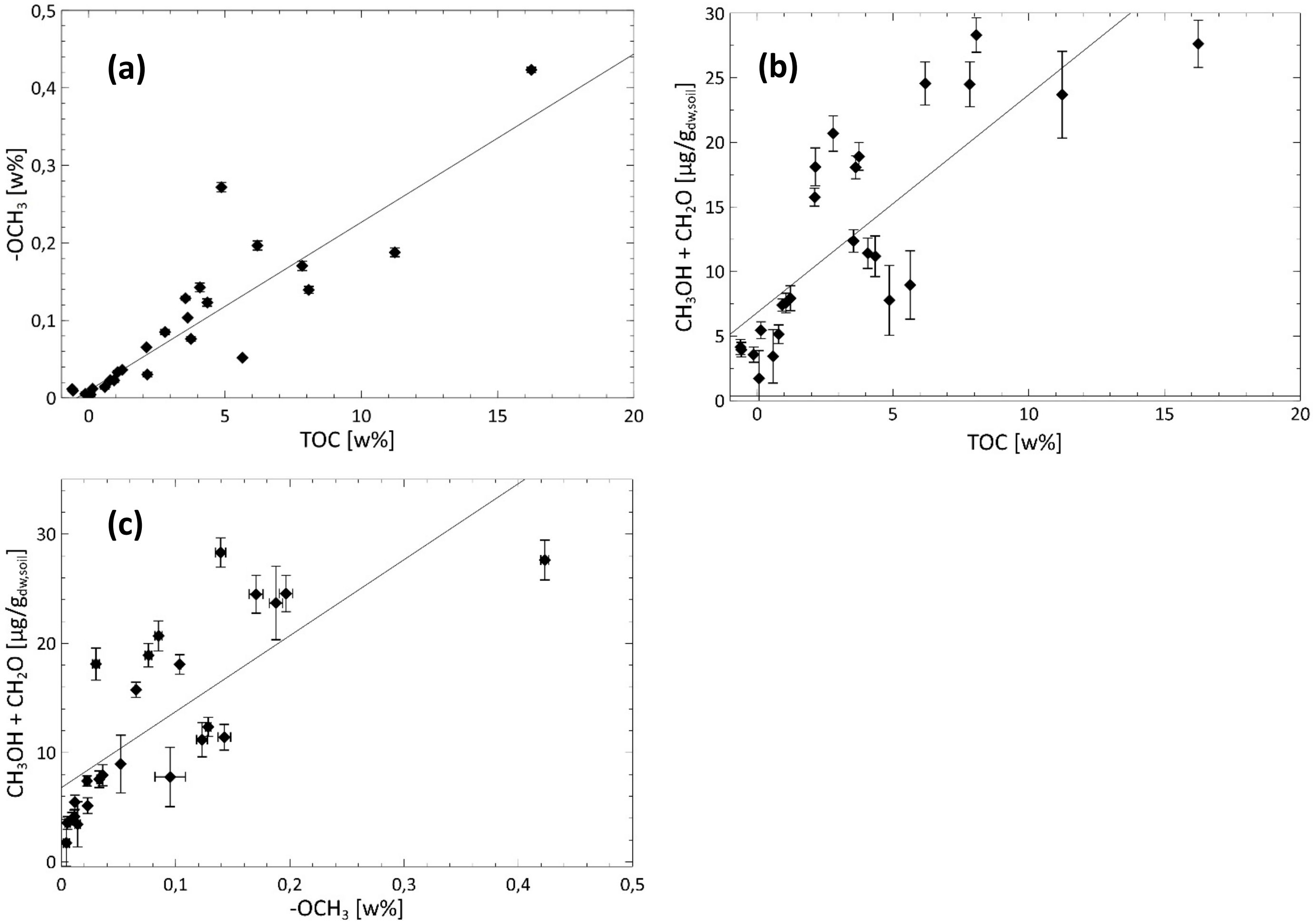

Figure S12. Relationship between soil TOC, methoxy content, and the formation of CH_3OH + CH_2O in sterile soils.

(a) Coefficient of determination (R^2 = 0.88) for the relationship between TOC (n=1) and methoxy group (–OCH_3) content (one soil sample, measured in triplicate, n=3) in the investigated soil samples, including additional samples from a previous study[1].

(b) Coefficient of determination (R^2 = 0.64) for the relationship between TOC (n=1) and the combined concentrations of CH_3OH and CH_2O (Error bars represent the SD of total CH_3OH and CH_2O conversion, based on the mean of three independent experiments (n=3), each measured in triplicate.) in the same soil samples.

(c) Coefficient of determination (R^2 = 0.59) for the relationship between methoxy group content (n=3) and the combined concentrations of CH_3OH and CH_2O (Error bars represent the SD of total CH_3OH and CH_2O conversion, based on the mean of three independent experiments (n=3), each measured in triplicate.) in the same samples.

All experiments were conducted over a 48 h incubation period, during which 5 g of sterile soil was suspended in 10 mL of ultrapure H_2O.

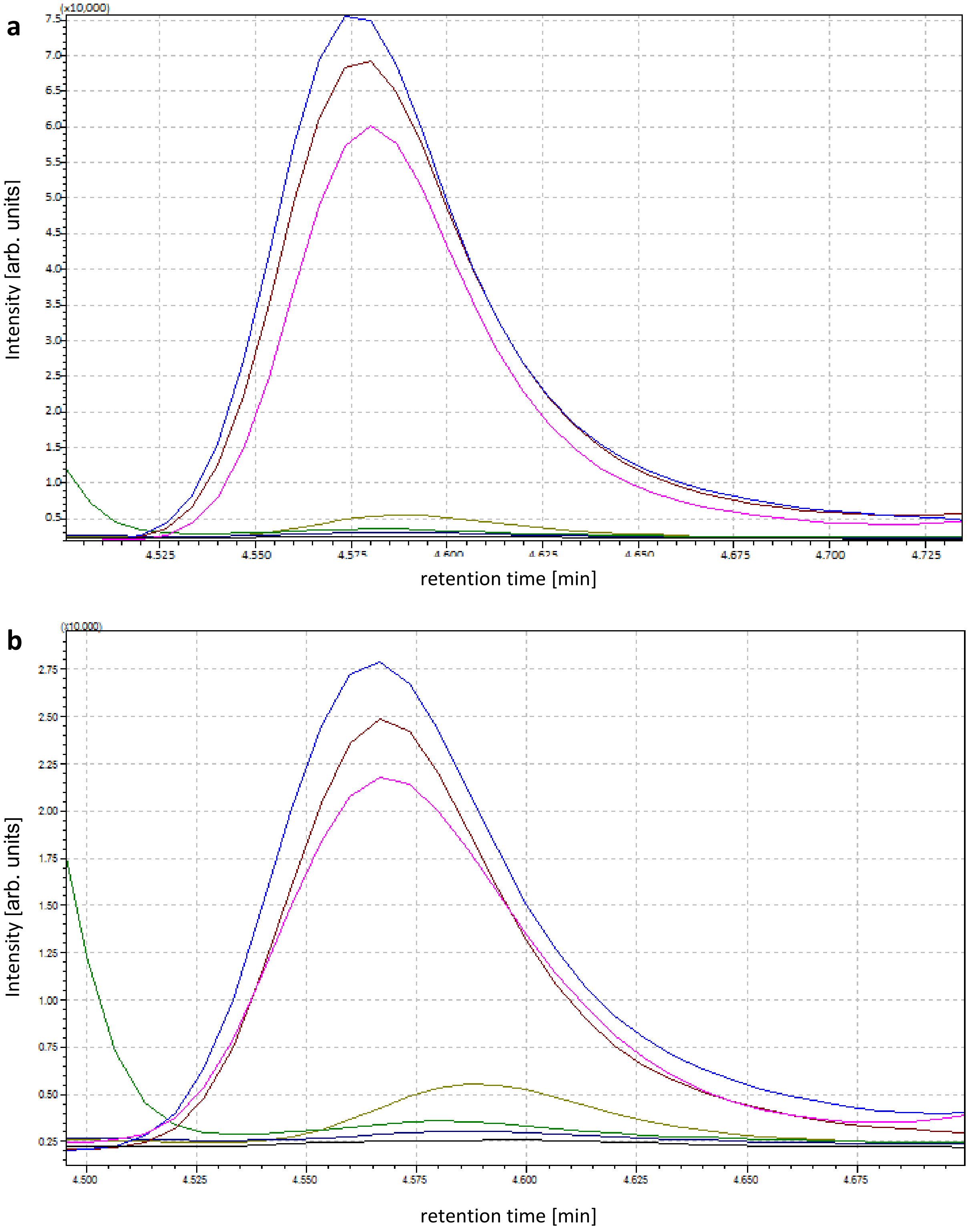

Figure S13. Chromatogram of labeled CH_3OH in soil experiments with added ^2H and ^{18}O labeled 2-methoxyphenol

Chromatogram of mass 33,34 and 35 of C^2H_3OH **(a)** or mass 33 and 34 of $CH_3{}^{18}OH$ **(b)** in experiments where the methoxy groups were removed from the soil samples GL1, PO1, and GL2 using the Zeisel method [2] with 1 g soil and 2 ml H_2O with added 5 µmol **(a)** d3-2-methoxyphenol and **(b)** 5 µmol ^{18}O-2-methoxyphenol. Blue, brown, and pink are the

experiments with labelled 2-methoxyphenol (large peaks), light green, dark green, and purple are the non-spiked soils (small to no peaks), and black (no peak) is pure H_2O. The production of labeled CH_3OH is clearly visible compared to the unlabeled soils and pure H_2O.

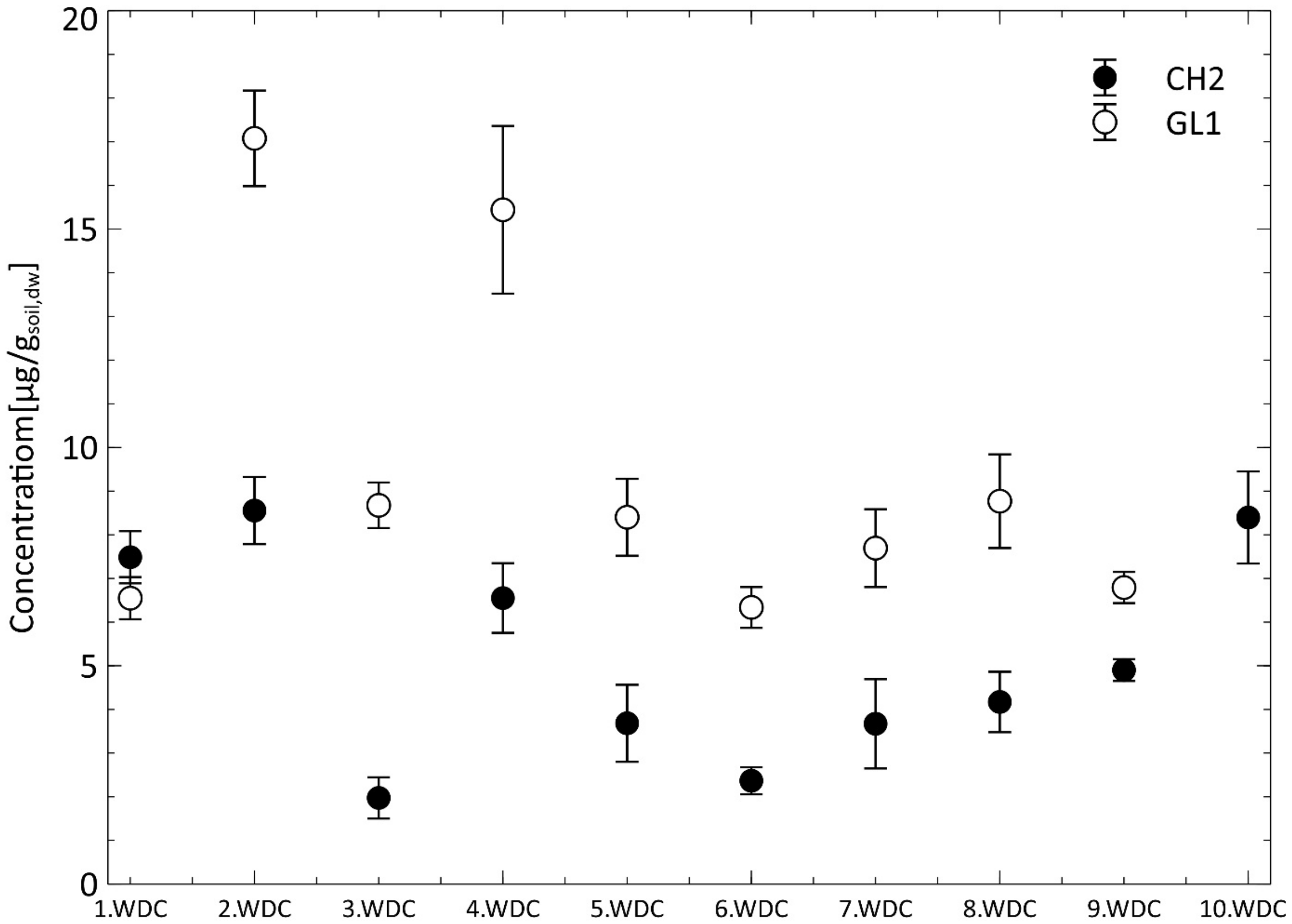

Figure S14. CH_2O measurements in wet-dry-cycles with soil samples CH2 and GL1

Concentrations of CH_2O were measured across ten wet-dry cycles of soil samples from CH2 and GL1. The reaction time was 48 h, followed by drying the soils at 105 °C for at least one day before rewetting them 10 times. All provided data were evaluated at 22 °C, ambient pressure (1013 mbar), and under N_2/O_2 atmosphere after 48 h reaction time. The concentrations changed from 7.49 ± 0.39 µg/$g_{soil,\ dw}$ and 6.55 ± 0.75 µg/$g_{soil,\ dw}$, to 4.90 ± 0.20 µg/g $_{soil,\ dw}$ and 8.40 ± 1.05 µg/g $_{soil,\ dw}$, respectively (at 10 WDC). CH_2O data from GL1 could not be measured. This results in a total sum of 46.0 and 90.0 µg/$g_{soil,dw}$ of CH_2O. When converted to the total methoxy content in the soil, this represents a conversion of 4.44 and 4.79 % of the methoxy groups to CH_2O. Error bars represent the SD of total CH_2O conversion, based on the mean of three independent experiments (n=3), each measured in triplicate.

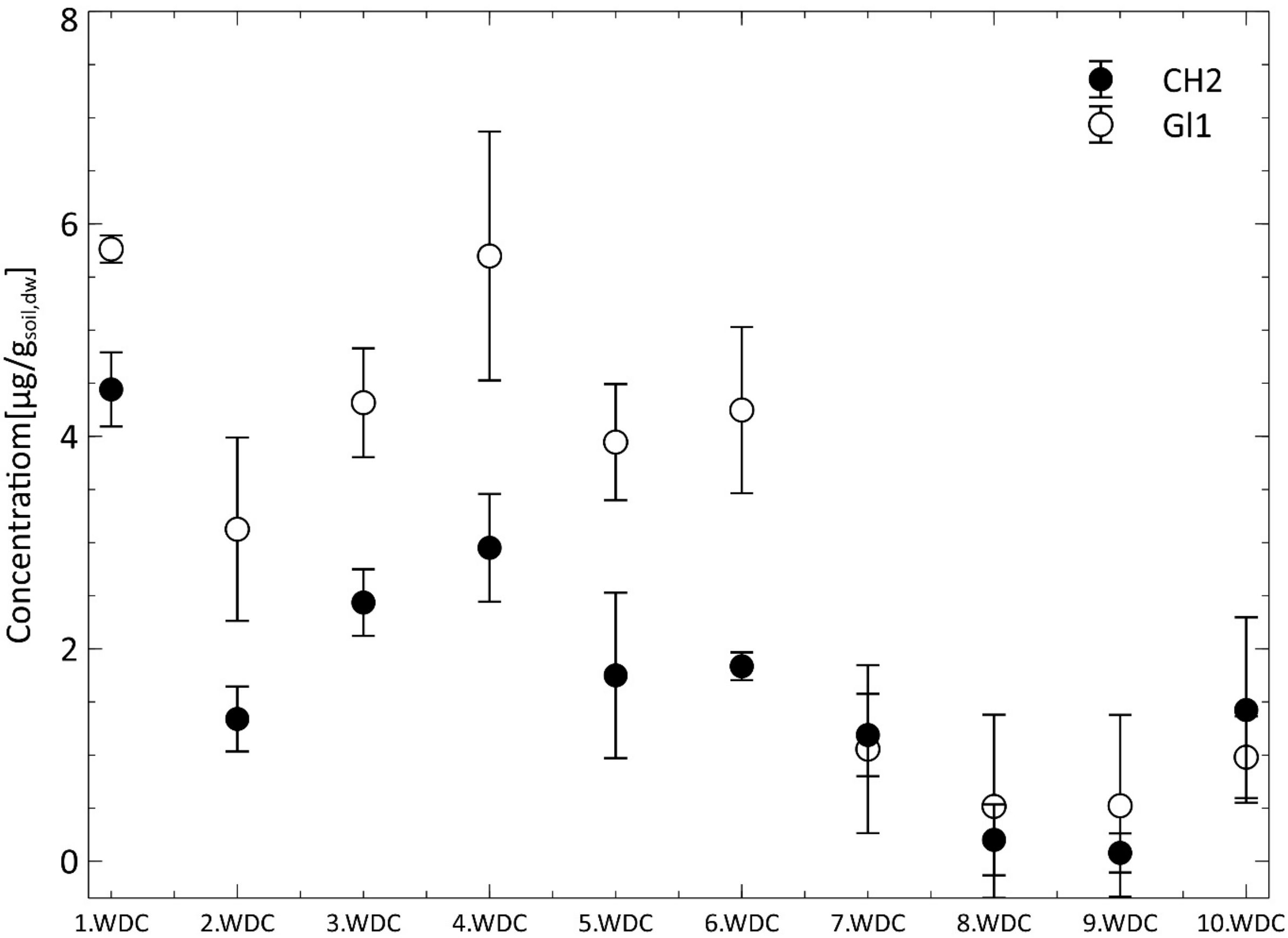

Figure S15. CH_3OH measurements in wet-dry-cycles with soil samples CH2 and GL1

Concentrations of CH_3OH were measured across ten wet-dry cycles of soil samples from CH2 and GL1. The reaction time was 48 h, followed by drying the soils at 105 °C for at least one day before rewetting and drying them 10 times. All provided data were evaluated at 22 °C, ambient pressure (1013 mbar), and under N_2/O_2 atmosphere after 48 h reaction time. The starting concentrations are 4.44 ± 0.21 µg/$g_{soil,dw}$ and 5.76 ± 0.35 µg/$g_{soil,dw}$, and the final concentrations are 1.42 ± 0.63 µg/$g_{soil,dw}$ and 0.98 ± 0.25 µg/$g_{soil,dw}$, respectively. This results in a total sum of 18.1 and 31.6 µg/$g_{soil,dw}$ of CH_3OH. When converted to the total methoxy content in the soil, this represents a conversion of 1.74 % and 1.68 % of the methoxy groups to CH_3OH. Error bars represent the SD of total CH_3OH conversion, based on the mean of three independent experiments (n=3), each measured in triplicate.

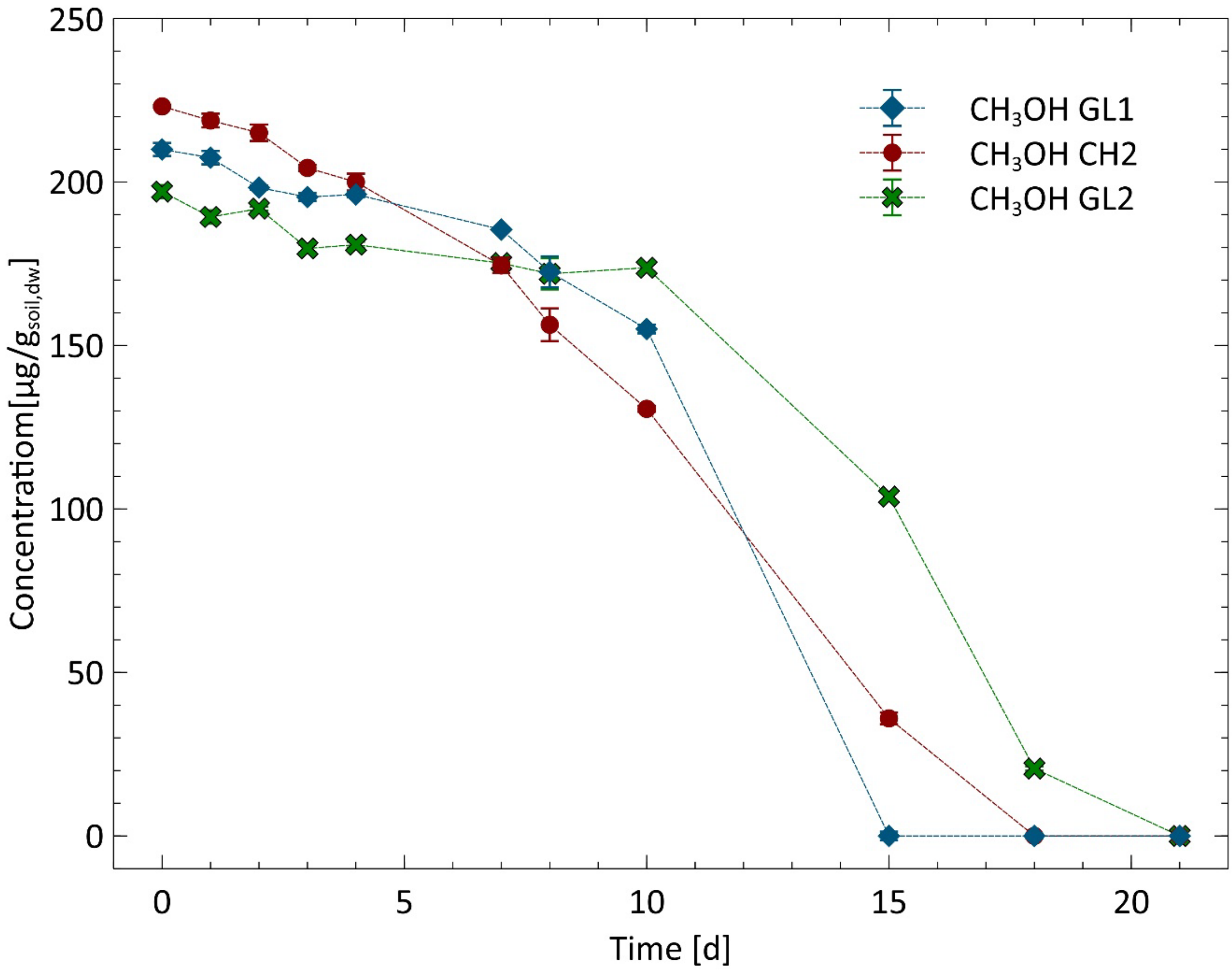

Figure S16. Degradation of CH_3OH in fresh soils (GL1, CH2, and GL2) due to methylotrophic microorganisms.

Concentrations of CH_3OH were measured over 21 days in one experiment for each soil (50 g) suspended in 100 mL ultra-pure H_2O and stored at 22 °C under ambient pressure (1013 mbar) and N_2/O_2 atmosphere. The error bars represent the SD of the total conversion of CH_3OH in one experiment with triple measurements (n=3).

The experiments contain 50 g of natural soil, which was transferred into a 500 mL Schott bottle (Duran, Germany), and 100 mL of an aqueous 100 ppmw CH_3OH solution was added. The bottle was sealed airtight and stored at 22 °C under ambient pressure (1013 mbar) and N_2/O_2 atmosphere. Over 21 days, eleven samples were taken from the experiments. Thereby, 2 mL of the liquid phase was collected and filtered using a 0.2 µm sterile syringe filter (WhatmanTM, GE Healthcare Life Sciences, USA). The liquid was stored in a 1.5 mL crimp glass vial (IVA Analysetechnik GmbH &Co.KG, Germany) and analyzed for its CH_3OH concentration.

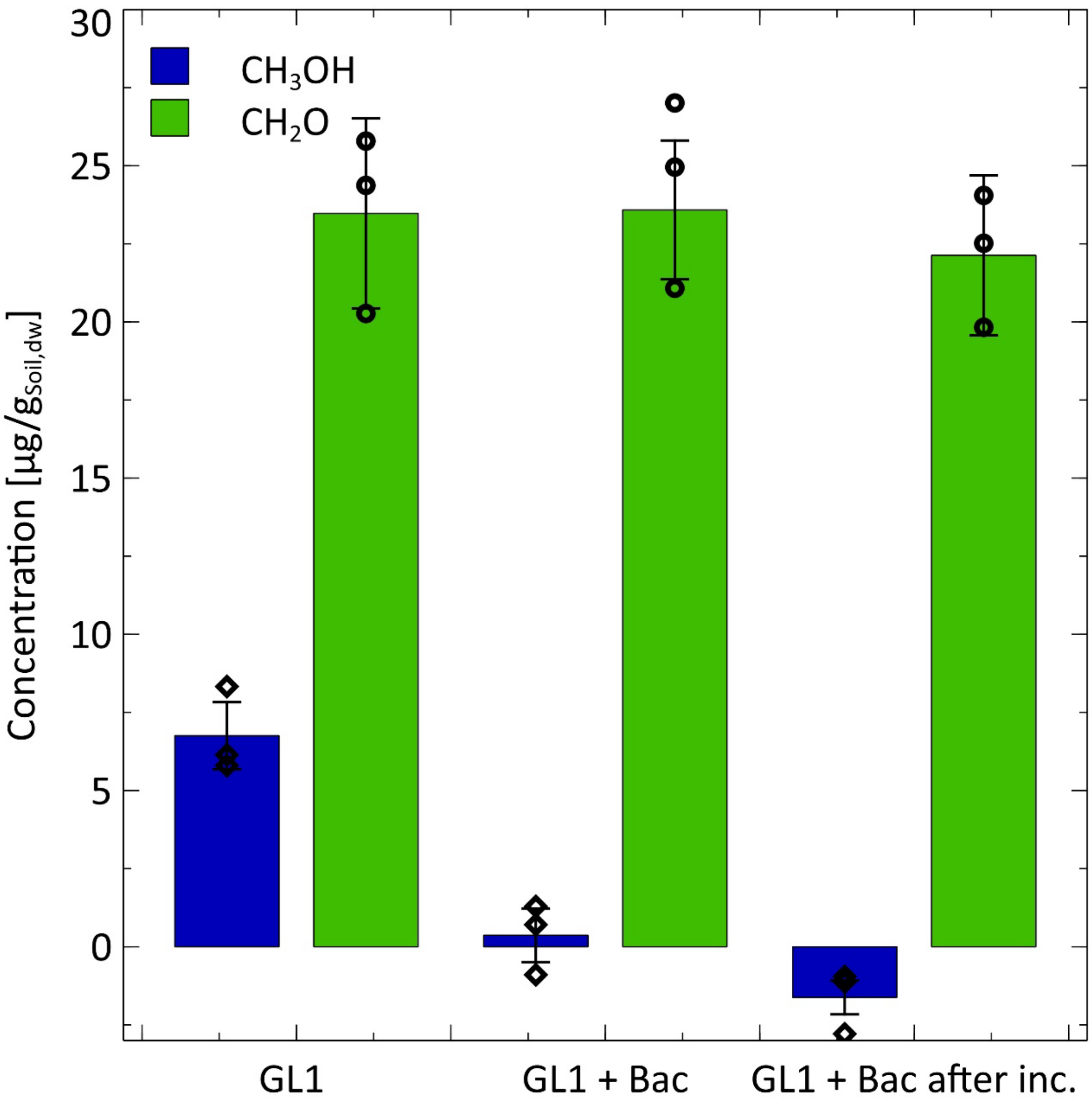

Figure S17. Degradation of abiotically produced CH_3OH in GL1 due to added methylotrophic bacteria

Addition of Methylorubrum extorquens bacteria prior to and after the soil experiment GL1, with CH_3OH degradation in both experiments, and no changes in CH_2O concentration. CH_3OH and CH_2O measurements after 5 days in the experiment, when the bacteria were added after the abiotic experiment. All provided data were evaluated at 22 °C, ambient pressure (1013 mbar), and under N_2/O_2 atmosphere after 48 h reaction time. Error bars represent the SD of total CH_3OH and CH_2O conversion, based on the mean of three independent experiments (n=3), each measured in triplicate. Diamonds (CH_3OH) and circles (CH_2O) indicate the mean value of each independent experiment.

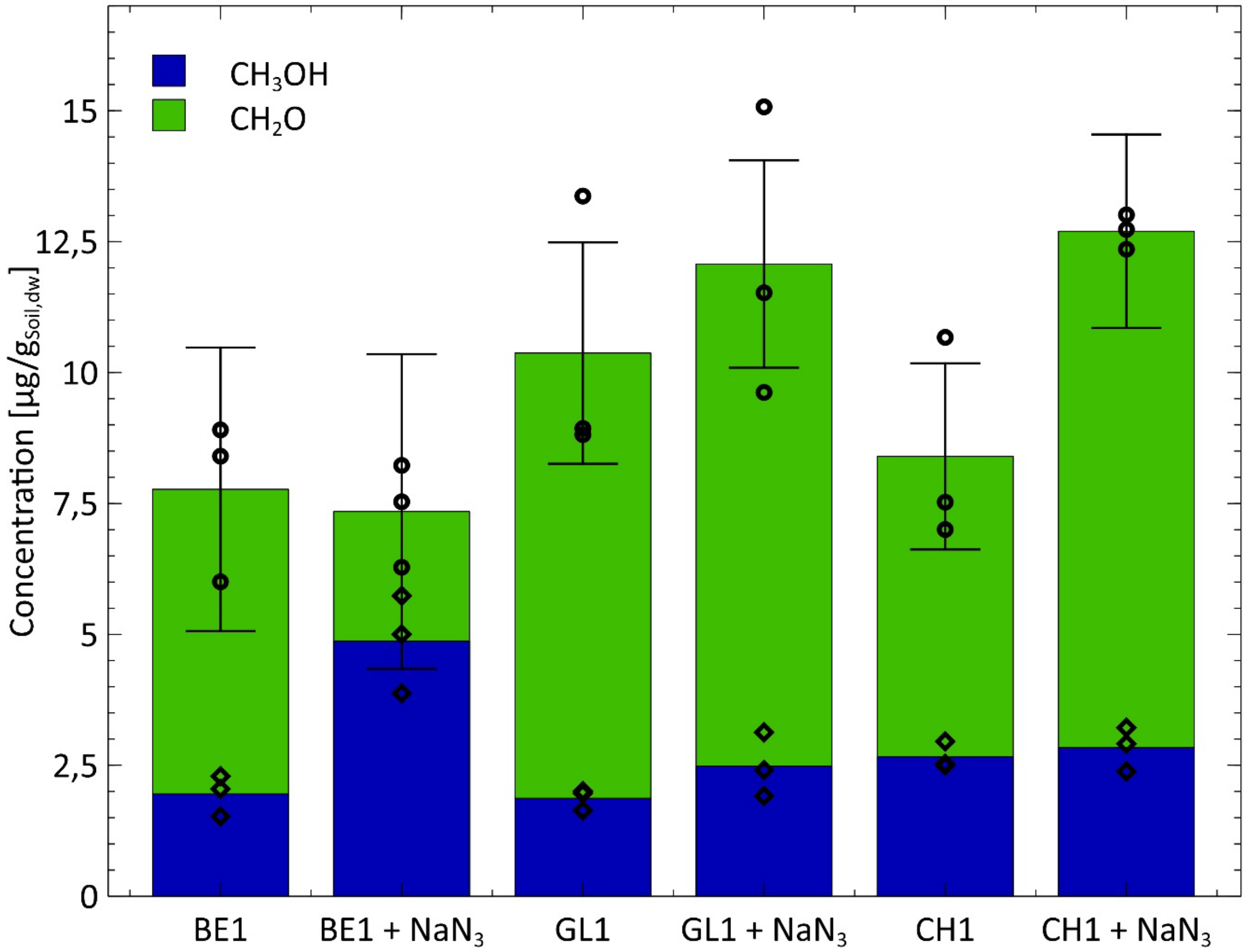

Figure S18. Formation of CH_3OH and CH_2O from soils BE1, GL1 and CH1 with and without addition of NaN_3

Formation of CH_3OH and CH_2O in sterilized soil samples (BE1, GL1, and CH1) with and without the addition of sodium azide (NaN_3), an inhibitor of enzymatic activity and microbial metabolism. No significant differences within the error bars were observed between treatments, although a slight increase in CH_3OH and CH_2O production was detected for soil CH1. Overall, the results support an abiotic origin of CH_3OH and CH_2O formation and indicate that residual enzymatic activity or intracellular metabolites do not contribute significantly to the observed production. All provided data were evaluated at 22 °C, ambient pressure (1013 mbar), and under N_2/O_2 atmosphere after 48 h reaction time. Error bars represent the SD of total CH_3OH and CH_2O conversion, based on the mean of three independent experiments (n=3), each measured in triplicate. Diamonds (CH_3OH) and circles (CH_2O) indicate the mean value of each independent experiment.

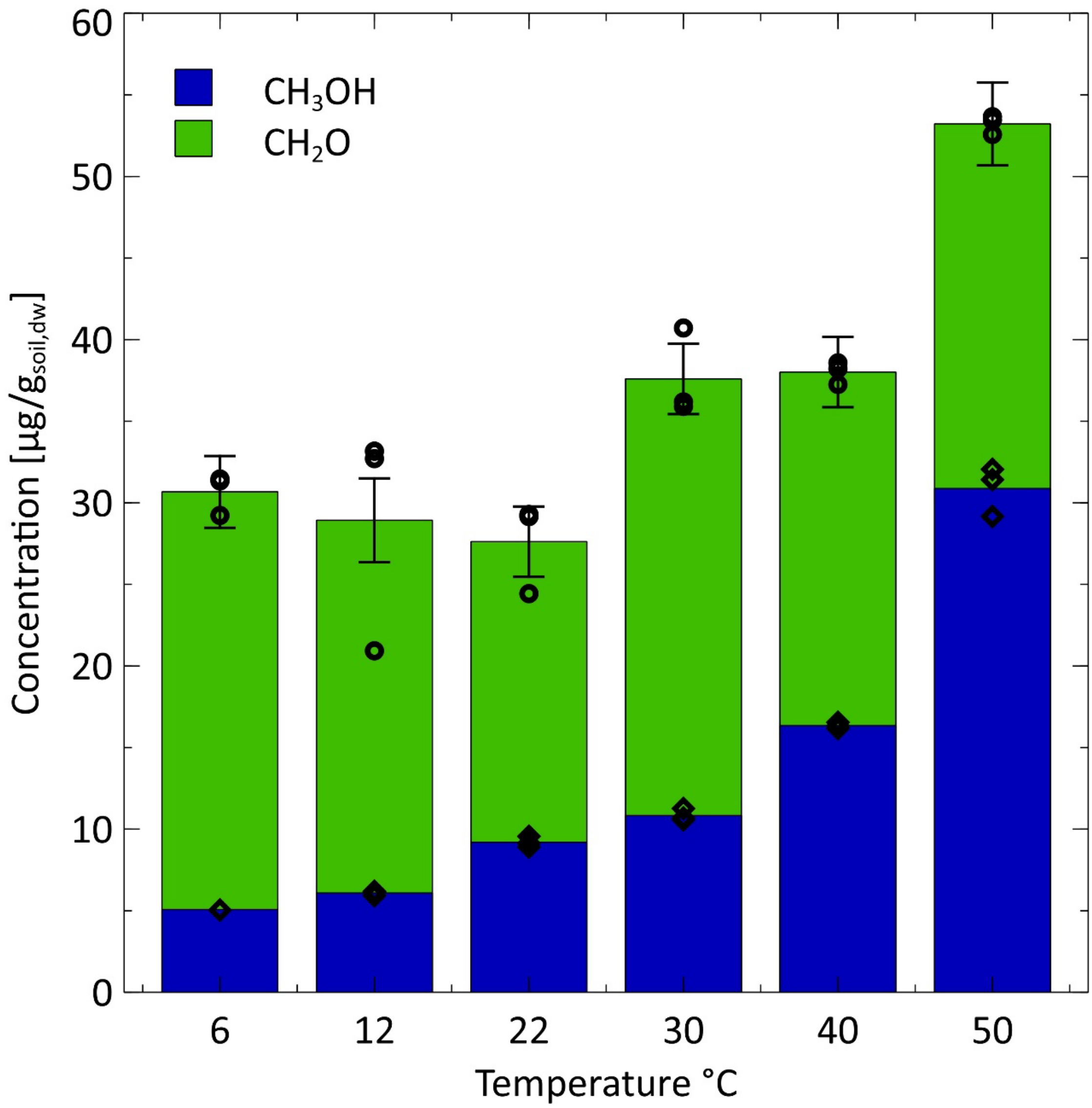

Figure S19. Measurement of CH_3OH and CH_2O in soil GL2 as a function of temperature

Formation of CH_3OH and CH_2O from soil sample GL2 incubated under sterile aqueous conditions with different temperatures (6 – 50 °C). Soils (5 g each) were suspended in ultrapure water (10 mL). All provided data were evaluated at ambient pressure (1013 mbar), and under N_2/O_2 atmosphere after 48 h reaction time. Error bars represent the SD of total CH_3OH and CH_2O conversion, based on the mean of three independent experiments (n=3), each measured in triplicate. Diamonds (CH_3OH) and circles (CH_2O) indicate the mean value of each independent experiment. Diamonds (CH_3OH) and circles (CH_2O) indicate the mean value of each independent experiment.

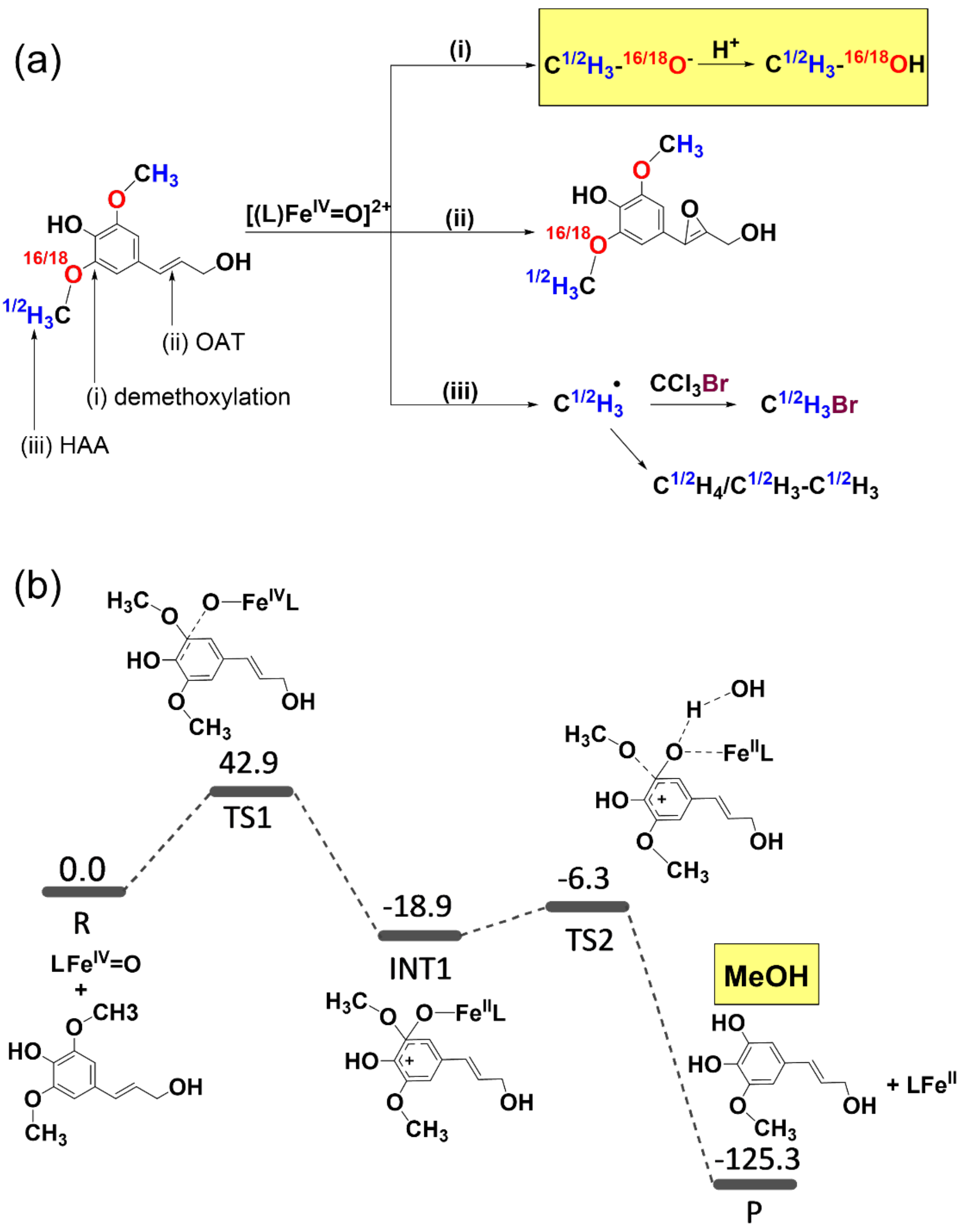

Figure S20. Reproduction of Figure 1, showing **(a)** the possible pathways for the reaction with monolignols and **(b)** the computed potential energy surface of $[(L)(OH_2)Fe^{IV}{=}O]^{2+}$ with sinapyl alcohol (UB3LYP-D3), illustrating key intermediates and transition states along the proposed demethoxylation pathway.

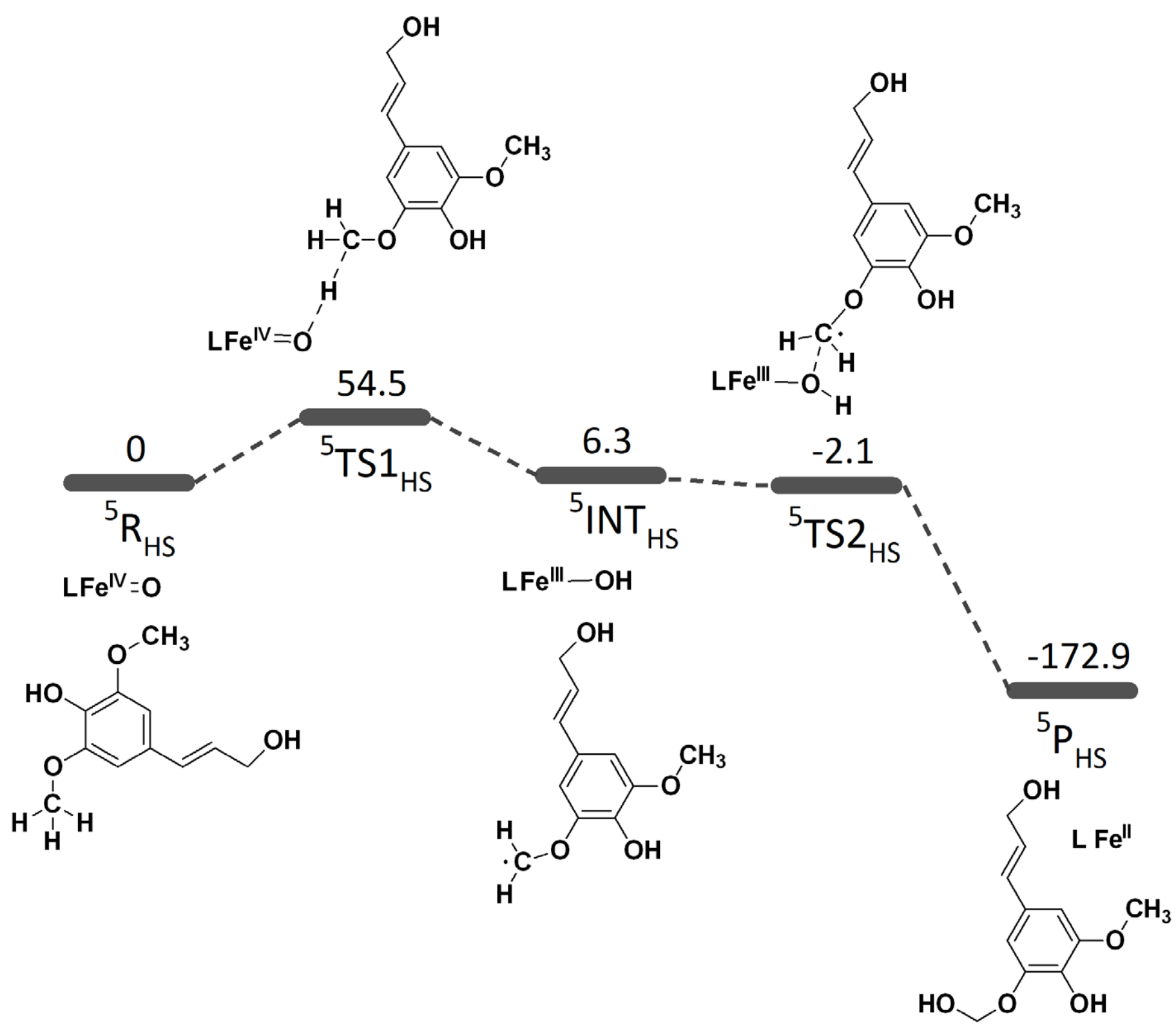

Figure S21. Computed reaction profile of the HAA mechanism of sinapyl alcohol.

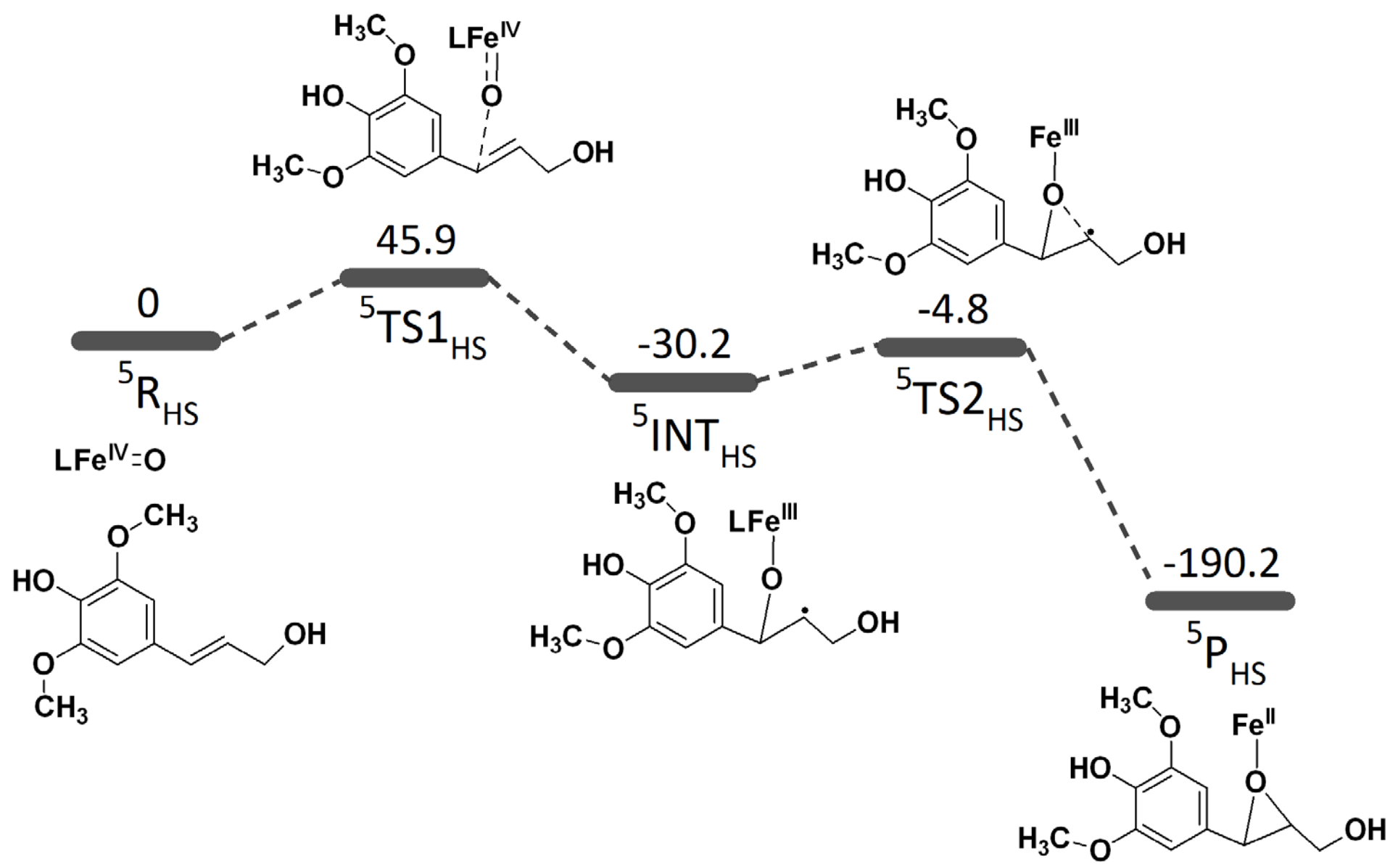

Figure S22. Computed reaction profile of the OAT mechanism of sinapyl alcohol.

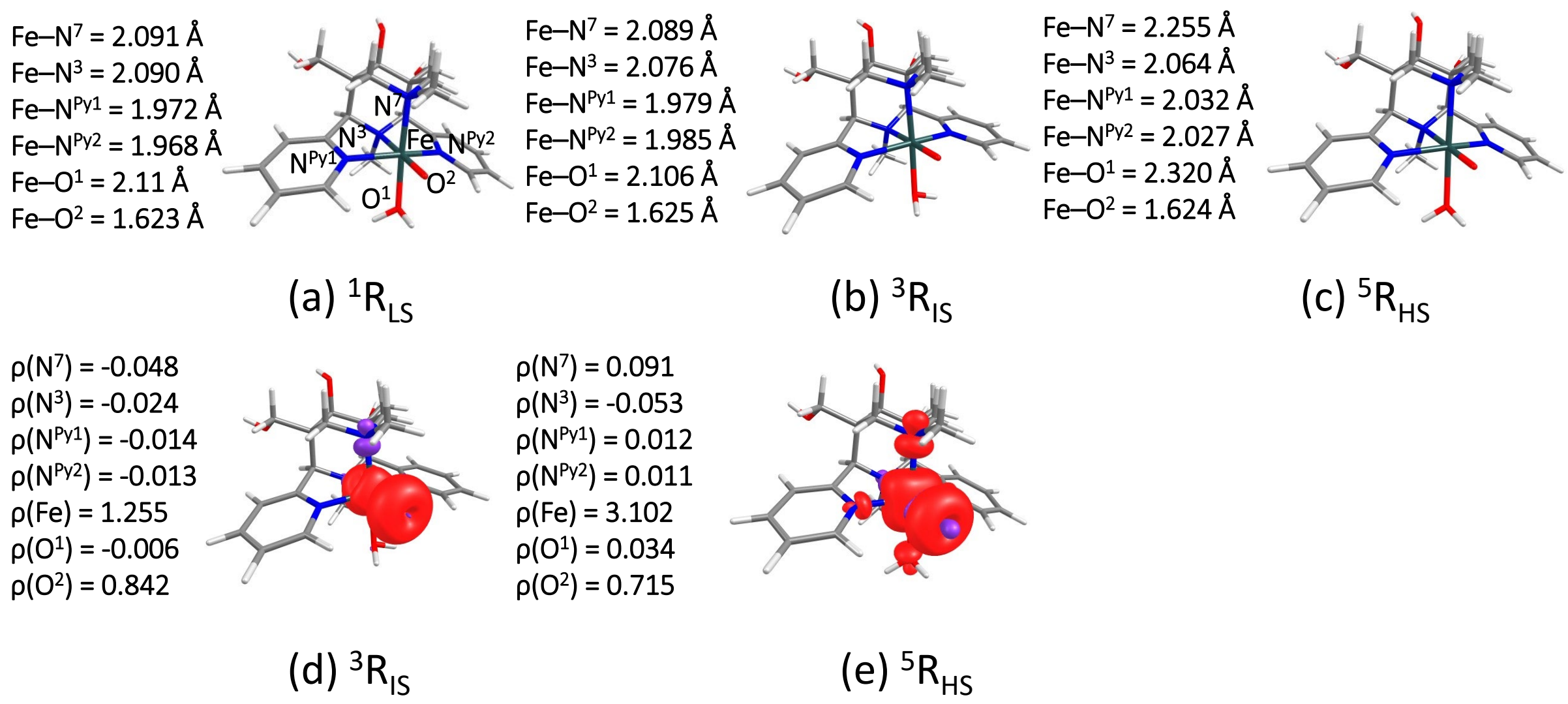

Figure S23. Optimized geometries (a-c) and spin density plots (d-e) for $[(L)(OH_2)Fe^{IV}{=}O]^{2+}$.

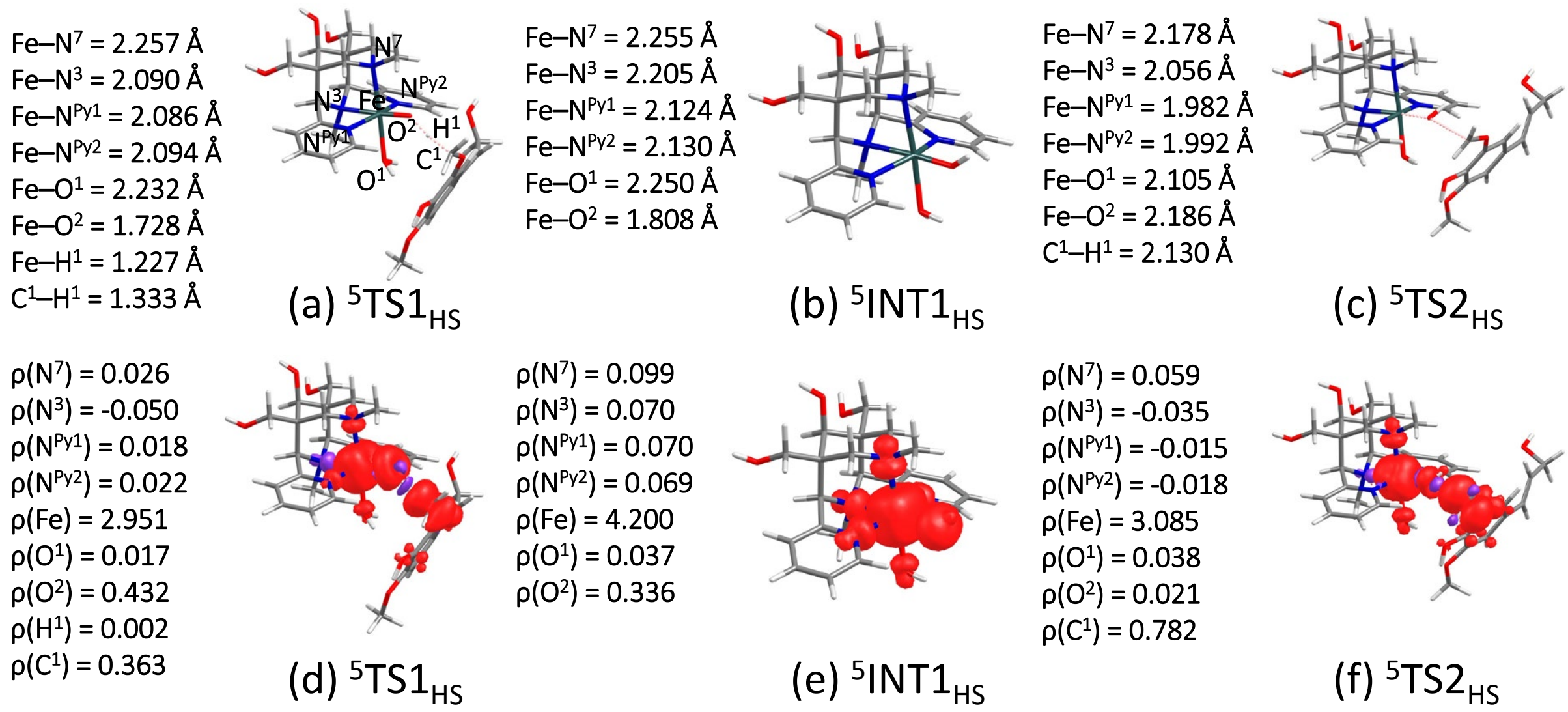

Figure S24. Optimized geometries (a-c) and spin density plots (d-f) of HAA mechanism for $^{5}TS1_{HS}$, $^{5}INT1_{HS}$ and $^{5}TS2_{HS}$.

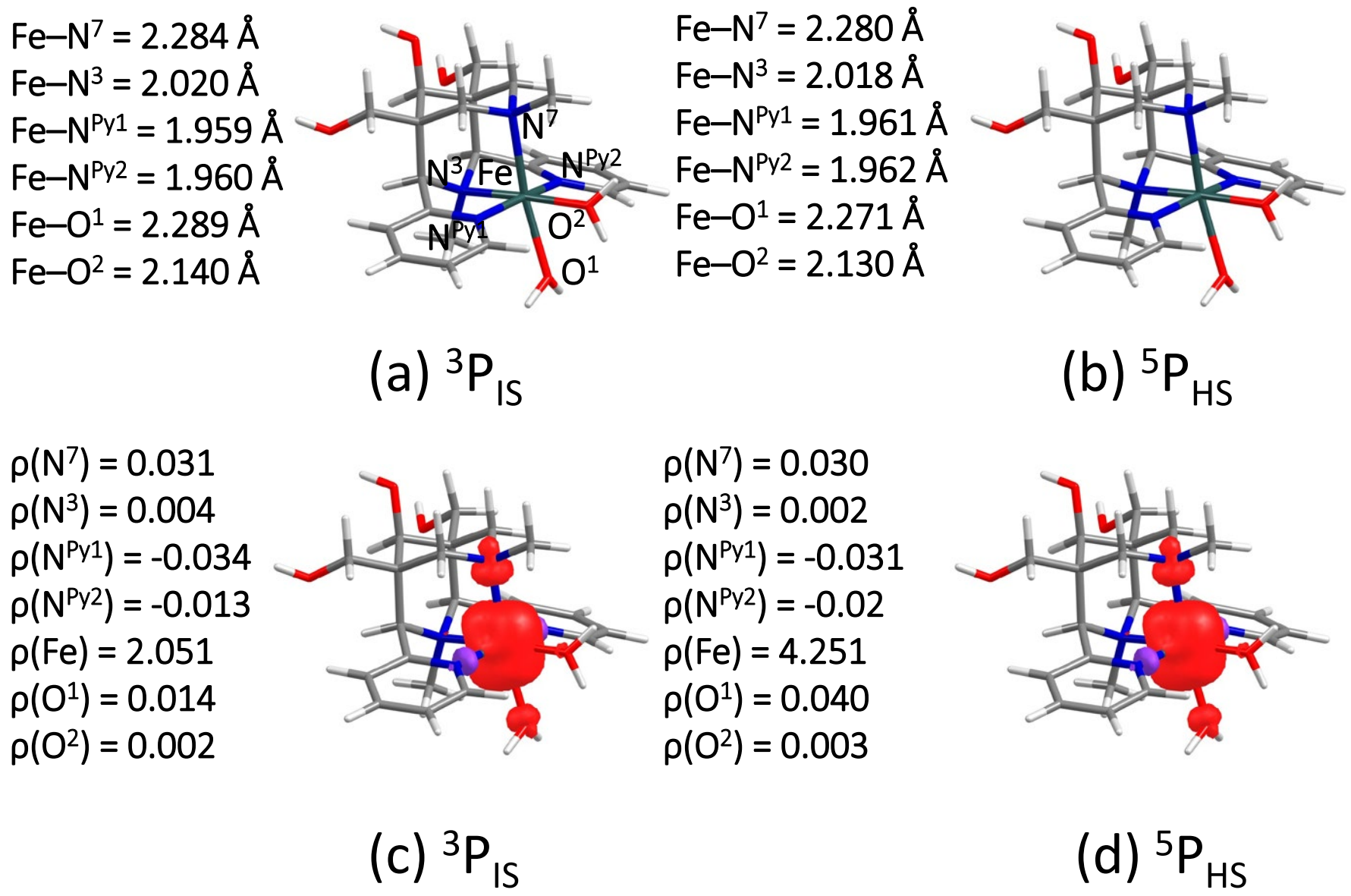

Figure S25. Optimized geometries (a-b) and spin density plots (c-d) of HAA mechanism for $^{3}P_{IS}$, and $^{5}P_{HS}$.

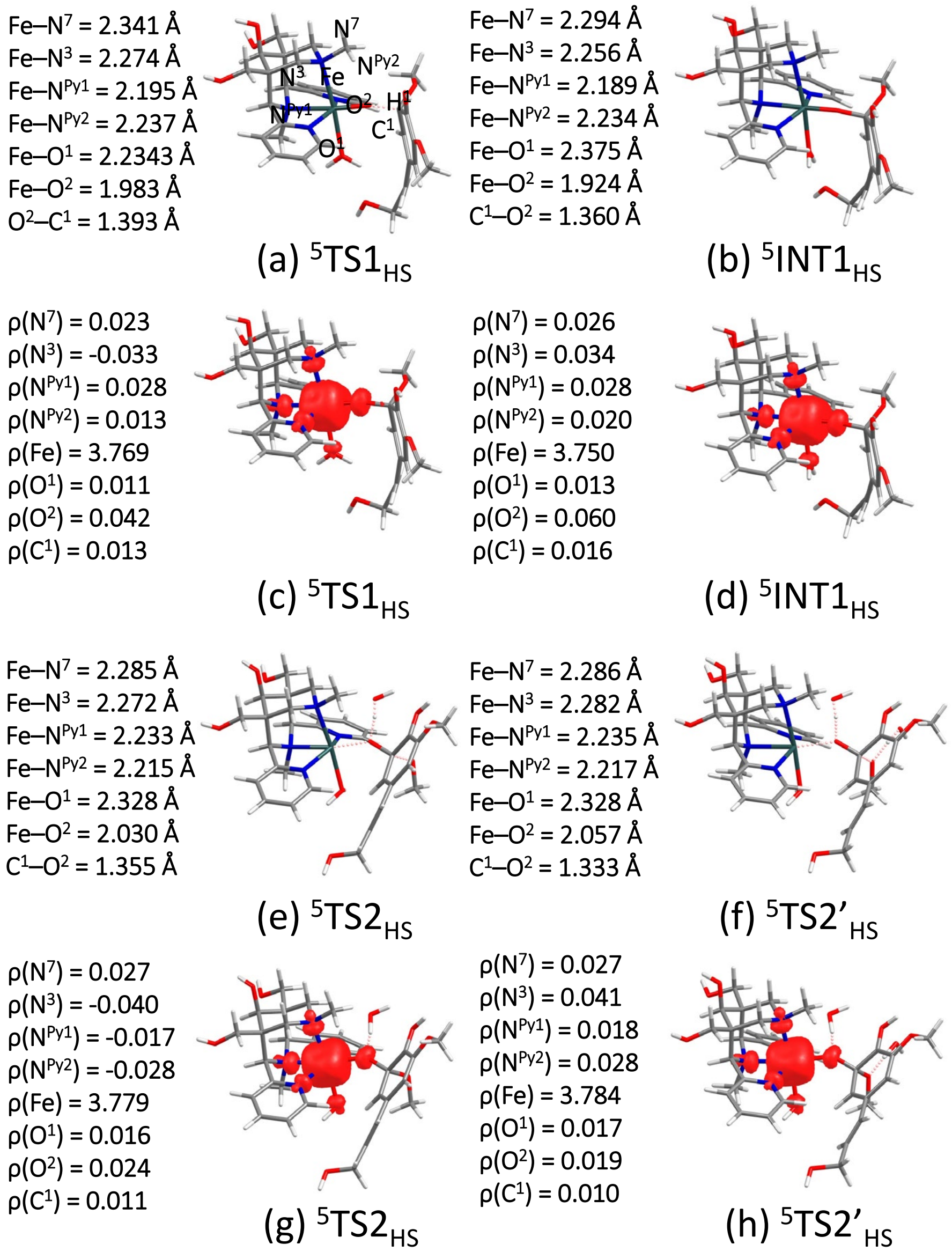

Figure S26. Optimized geometries and spin density plots of demethoxylation mechanism for ^{3}TS1$_{HS}$ (a, c), ^{3}INT1$_{HS}$ (b, d), ^{3}TS2$_{HS}$ (e, g) and ^{3}TS2’$_{HS}$ (f, h).

To simulate an outer-sphere electron transfer mechanism, the formation of a substrate-catalyst complex, also involving HOtf molecules was examined. Initially, the Fe^{IV}-oxido center interacts with two HOtf molecules, forming an $[(L)Fe^{IV}{=}O(OH_2)...(HOtf)_2]$ precursor at -99kJ/mol. This reacts with a synapyl alcohol substrate molecule to the reactant complex (RC), which is highly stable with an energy of -139.5kJ/mol. Spin density values confirm that no electron transfer occurs during the formation of the reactant complex. In the subsequent step, the substrate approaches one of the HOtf molecules, where the close proximity to HOtf enables a PCET in a slightly endothermic step (+10.4kJ/mol). These findings suggest that both HAA and OAT reactions can occur via a PCET mechanism, with the HOtf molecule facilitating electron transfer through proton donation, indicating an outer-sphere electron transfer pathway (Figures S27-S29).

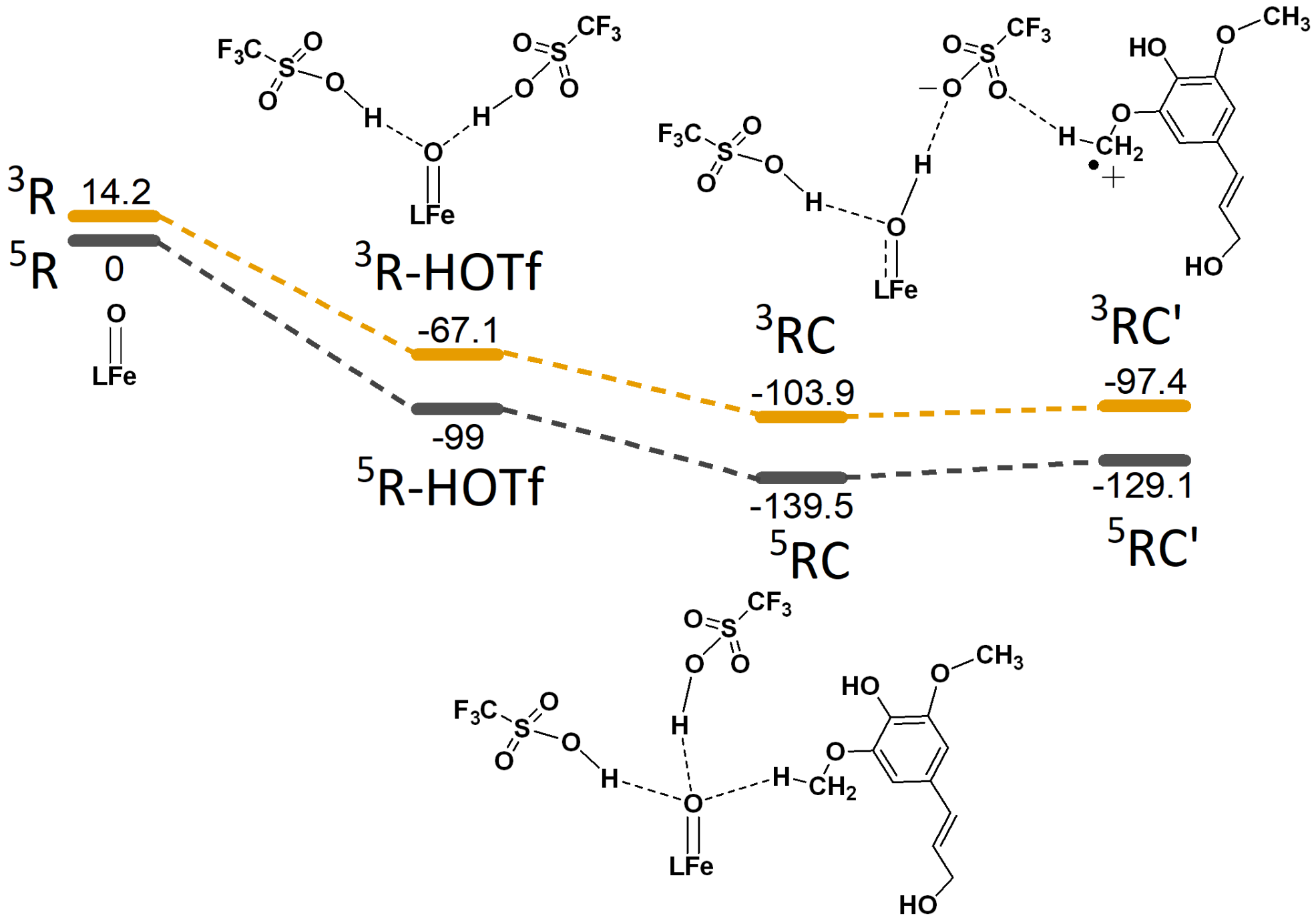

Figure S27. Computed potential energy surface of $[(L)(OH_2)Fe^{IV}{=}O]^{2+}$ forming a proton coupled electron transfer reactant complex.

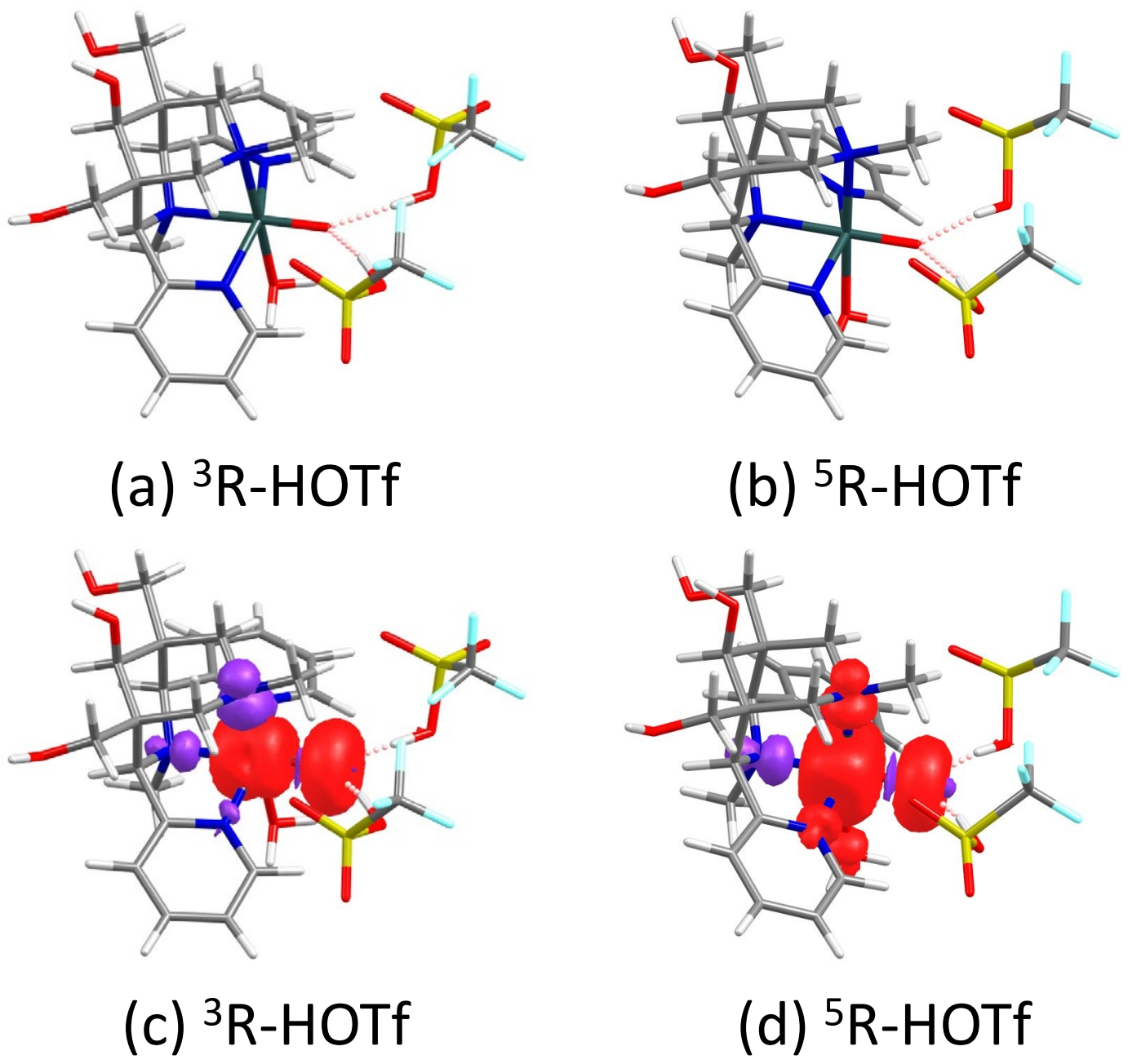

Figure S28. Optimized geometries (a-b) and spin density plots (c-d) of OSET mechanism for ^{3}R-HOTf and ^{5}R-HOTf.

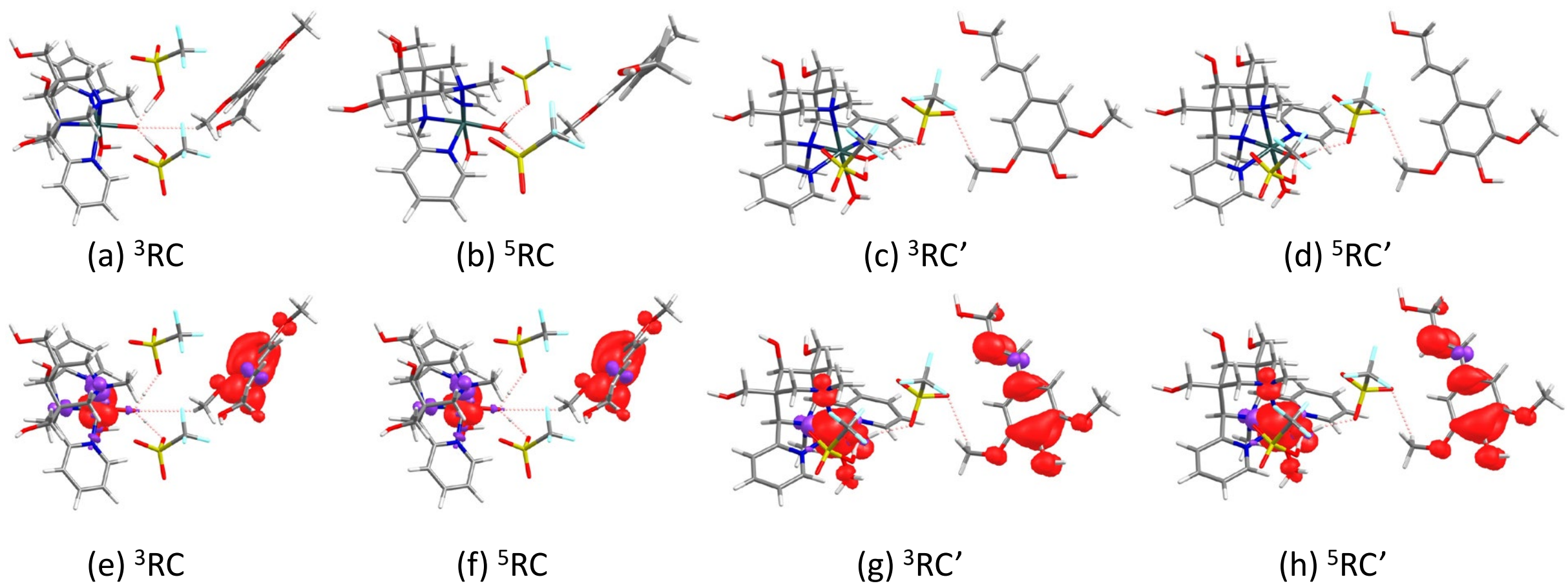

Figure S29. Optimized geometries (a-d) and spin density plots (e-h) of OSET mechanism for ^{3}RC, ^{5}RC, ^{3}RC’ and ^{5}RC’.

References

1. Hädeler, J. *et al.* Natural Abiotic Iron-Oxido-Mediated Formation of C1 and C2 Compounds from Environmentally Important Methyl-Substituted Substrates. *J. Am. Chem. Soc.* **145**, 24590-24602; 10.1021/jacs.3c06709 (2023).
2. Zeisel, S. Über ein Verfahren zum quantitativen Nachweise von Methoxyl. *Monatshefte für Chemie* **6,** 989–997; 10.1007/bf01554683 (1885).
3. Page, S. E., Sander, M., Arnold, W. A. & McNeill, K. Hydroxyl radical formation upon oxidation of reduced humic acids by oxygen in the dark. *Environ. Sci. Technol* **46,** 1590–1597; 10.1021/es203836f (2012).
4. Zhang, N. *et al.* Dependence of Biotic and Abiotic H2O2 and •OH Production on the Redox Conditions and Compositions of Sediment during Oxygenation. *Environ. Sci. Technol* **58,** 3849–3857; 10.1021/acs.est.3c10424 (2024).
5. Page, S. E. *et al.* Dark formation of hydroxyl radical in Arctic soil and surface waters. *Environ. Sci. Technol* **47,** 12860–12867; 10.1021/es4033265 (2013).
6. Vermilyea, A. W., Dixon, T. C. & Voelker, B. M. Use of H2(18)O2 to measure absolute rates of dark H2O2 production in freshwater systems. *Environ. Sci. Technol* **44,** 3066–3072; 10.1021/es100209h (2010).
7. Schwertmann, U. Solubility and dissolution of iron oxides. *Plant Soil* **130,** 1–25; 10.1007/BF00011851 (1991).
