# Supplementary material for: Iron-induced demethoxylation of lignin as an important source for methanol and its oxidation products in the environment
Source: Nat Commun. 2026 Aug 13;17:8334. doi: 10.1038/s41467-026-76679-x (PMC13473683; doi:10.1038/s41467-026-76679-x)
Supplement: Supplementary file 2 [file 41467_2026_76679_MOESM2_ESM.pdf]

nature portfolio

Peer Review File

Iron-induced demethoxylation of lignin as an important source for methanol and its oxidation products in the environment

Corresponding Author: Professor Frank Keppler

This file contains all reviewer reports in order by version, followed by all author rebuttals in order by version.

Version 0:

Reviewer comments:

Reviewer #1

(Remarks to the Author)
This study discovered a novel abiotic demethoxylation mechanism mediated by Iron-induced demethoxylation of lignin under ambient conditions without requiring elevated temperature and pressure. This finding represents a highly interesting contribution with potentially important implications for soil carbon turnover and global climate change.

However, I have a few major concerns:

1. Anaerobic or aerobic processes for soil carbon turnover would be fundamentally different. The rationale for incubating soils under wetting conditions and wet-dry cycles is not clearly justified for me.

Were any experiments conducted under dry or aerobic incubation conditions? This is particularly relevant given that Methylorubrum extorquens is an aerobic bacterium. You could also consider to add corresponding methanogens to utilize C1 anaerobically. Or as mentioned below in L306, just to inhibit bacteria activity to see changes of CH_3OH and CH_2O.

2. Regarding the representativeness of the soil samples: it would be valuable to include soils with varying background levels of lignin/methoxyl groups, Fe_2O_3, and H_2O_2.

Have you considered to employ one or two soil types and experimentally supplement them with different concentrations of these components after determining their baseline levels? Not essential to be in this study and supplementing lignin/methoxyl groups may not be necessary as you have manipulatively removing the methoxyl compounds.

3. The contribution of this mechanism to the overall soil carbon turnover or greenhouse gas emission should be evaluated by considering anaerobic or aerobic conditions separately.

Minor comments:

L79 would anaerobic or aerobic environment matter?
L81 is there any other alternative methyl-based precursors?
L82 is there any other sources?

L117 why 110%?

L246 comparable to the natural abundances of Fe2O3 and H2O2existence in soils?

L255 why in water? Anaerobically or aerobically?

L256 to present information for levels of lignin, methoxy content, Fe2O3 and H2O2 in these soils?

L269 why wet-dry cycles? How long for each condition?
L269.representative for what?

L306 where is the results using dried soil samples? In wet-dry cycles?
L306 you can also inhibit bacteria activity to have a test

L326-327 that may only be applied to wetland ecosystem with frequent wet-dry cycles
L328 how did you extrapolate?

L335 aerobically

L340 how much?

Reviewer #2

(Remarks to the Author)
What are the noteworthy results?

The paper present a new abiotic mechanism to degrade lignin in the soil. The process is of interest and new even though the same gorup has done some publications in J. Am. Chem. Soc.

Will the work be of significance to the field and related fields?
The significance is not clear. It has to be better stated in a specific paragraph of the discussion section which has to be separated from the results section.

How does it compare to the established literature?
The literature review in the part of the manuscript that follows the abstract (which should be named "introduction") is very poor. It has to be incrased also considering the general effect of iron on soil organic matter, see the work of:

Hall, S. J., & Silver, W. L. (2013). Iron oxidation stimulates organic matter decomposition in humid tropical forest soils. Global change biology, 19(9), 2804-2813.

Chen, C., Hall, S. J., Coward, E., & Thompson, A. (2020). Iron-mediated organic matter decomposition in humid soils can counteract protection. Nature communications, 11(1), 2255.

Jones, M. E., LaCroix, R. E., Zeigler, J., Ying, S. C., Nico, P. S., & Keiluweit, M. (2020). Enzymes, manganese, or iron? Drivers of oxidative organic matter decomposition in soils. Environmental Science & Technology, 54(21), 14114-14123.

Dong, H., Zeng, Q., Sheng, Y., Chen, C., Yu, G., & Kappler, A. (2023). Coupled iron cycling and organic matter transformation across redox interfaces. Nature Reviews Earth & Environment, 4(9), 659-673.

Does the work support the conclusions and claims, or is additional evidence needed?
Figure 1 please provide yields on a mass basis also on a separate figure.

Is the methodology sound?
Methodology is sound

Is there enough detail provided in the methods for the work to be reproduced?
Methods are sufficiently detailed

Reviewer #3

(Remarks to the Author)
This manuscript presents an abiotic iron-induced demethoxylation mechanism for the production of oxygenated C1 compounds (CH_3OH, CH_2O) from lignin-derived methoxy groups under ambient conditions. The manuscript elucidates the significance of this pathway through the design of a model system, mechanistic validation, and its extension to real soil environments and the global scale. This work is well-structured and aligns with current interests in lignin valorization and bioeconomy. However, I cannot recommend acceptance of the paper in its current form due to the limited novelty of the approach and unconvincing experimental data presented. The overall quality of this work still falls far short of the standards required for publication in Nature communication.

1. The mechanism presented in the manuscript demonstrates limited novelty. Iron-mediated demethylation is already a well-established paradigm in both chemical and biological demethylation processes. The proposed "OAT-induced demethoxylation" in this study lacks sufficient innovation, as it essentially aligns with existing and commonly recognized

reaction types.
2. Although the manuscript emphasizes " ambient temperature and pressure conditions (22°C, 1013mbar)", the key mechanistic experiments were conducted at pH 2.3, which is inconsistent with the typical soil pH range as Table S2 showed. Additionally, high concentrations of exogenous H_2O_2 and synthetic ligand-iron complexes in the model system differ from the condition present in natural soils. So, the validity of a mechanism verified under such artificial conditions regarding its dominance in natural scenarios remains questionable.
3. It is recommended to consider key environmental variables such as pH gradients, the content of different iron minerals, and variations in their reactivity to investigate the environmental boundary conditions of the mechanism.
4. As illustrated in Figure S11, the correlation between either Total Organic Carbon (TOC) or methoxy group content (OCH_3) and the methoxy content with CH_3OH+CH_2O production in sterile soils, the R^2 values are only 0.67 or 0.60, respectively. This level of correlation appears insufficiently compelling. It is recommended to consider incorporating additional evaluation parameters for a more comprehensive analysis such as residual plot or others. Additionally, it is recommended to consider the relationship between the concentrations of H_2O_2 and high-valent iron species during the process and the generation of CH_3OH/CH_2O.
5. In natural soils, lignin-carbohydrate complexes and partially degraded natural lignin fragments are more widely distributed than lignin itself. The lack of experiments involving these components may reduce the reliability of extrapolating the conclusions to soil environments.
6. Can control experiments be established for the soil tests? Since soil sterilization may not completely eliminate certain unknown biologically active enzymes or the release of microbial intracellular metabolites. Consideration might be given to adding enzyme inhibitors and using artificially constructed soils as controls.
7. Line 268: Please add the reasons for selecting these two soil samples CH and GL1 for the wet-dry cycles experiments.
8. Please add error bars to the lower segments of the stacked columns in all bar charts, such as Figure 1, Figure 3(a), Figure S1, Figure S3, Figure S10, Figure S12(b), Figure S14, Figure S19.
9. The descriptive sentence for Table S1 on page 2 of the Supplementary Materials is incomplete.
10. The text on page 14 of the Supplementary Materials incorrectly refers to "Table S2" when it should be "Table S1".
11. The y-axis in Figure S11(b) is not fully displayed.

Version 1:

Reviewer comments:

Reviewer #1

(Remarks to the Author)
This manuscript has been improved substantially, and all my comments have been well-addressed.

Reviewer #2

(Remarks to the Author)
Paper can be accepted given that:
- the results are of impact
- the work is of significance
- conclusions are supported by data, methodology is sound
- No flaws detected
- the level of detail is satisfactory

Reviewer #3

(Remarks to the Author)
Authors had made the substantial effort to revise the manuscript, particularly in clarifying the mechanistic novelty and in providing additional experimental controls. The authors have addressed all my remaining concerns in a thorough manner.

Open Access This Peer Review File is licensed under a Creative Commons Attribution 4.0 International License, which permits use, sharing, adaptation, distribution and reproduction in any medium or format, as long as you give appropriate credit to the original author(s) and the source, provide a link to the Creative Commons license, and indicate if changes were made.
In cases where reviewers are anonymous, credit should be given to 'Anonymous Referee' and the source.
The images or other third party material in this Peer Review File are included in the article's Creative Commons license, unless indicated otherwise in a credit line to the material. If material is not included in the article's Creative Commons license and your intended use is not permitted by statutory regulation or exceeds the permitted use, you will need to obtain permission directly from the copyright holder.
To view a copy of this license, visit https://creativecommons.org/licenses/by/4.0/

Point-by-point responses to issues raised by Reviewer #1, #2 and #3

We sincerely thank all three reviewers for their careful evaluation of our manuscript and for their constructive and insightful comments. We greatly appreciate the time and expertise invested in reviewing our work. Their suggestions have helped us to substantially improve the clarity, structure, and scientific framing of the manuscript.

In response to the reviewers' comments, the manuscript has been extensively revised and restructured. In particular:

- The Introduction has been substantially expanded and refined to more clearly articulate the research gap and the significance of our findings in the context of existing literature.
- The mechanistic distinction between demethoxylation and previously reported demethylation pathways is now presented more explicitly.
- The environmental relevance of the proposed mechanism has been strengthened and clarified in both the Results and Discussion sections, including additional experiments addressing boundary conditions.
- Figures and supplementary material have been revised accordingly.
- The manuscript has been adjusted to fully comply with the formatting guidelines of *Nature Communications*.

Below, we provide detailed, point-by-point responses to each comment. Reviewer comments are reproduced in *italics*, followed by our responses in blue text. Line numbers refer to the revised manuscript. Please note that the manuscript has undergone extensive revisions and restructuring, including adjustments to meet the format requirements of *Nature Communications*. Because large parts of the manuscript were rewritten and reorganized, we provide a clean revised version rather than a track-changes document to ensure readability.

Reviewer #1

General comments:

This study discovered a novel abiotic demethoxylation mechanism mediated by Iron-induced demethoxylation of lignin under ambient conditions without requiring elevated temperature and pressure. This finding represents a highly interesting contribution with potentially important implications for soil carbon turnover and global climate change.

Authors: We thank the reviewer for the careful evaluation of our manuscript and the constructive comments provided. We appreciate the acknowledgement of the high relevance of our new mechanism and the value of the global implications for carbon cycle and global climate change.

Major concerns:

1. Anaerobic or aerobic processes for soil carbon turnover would be fundamentally different. The rationale for incubating soils under wetting conditions and wet-dry cycles is not clearly justified for me.

Authors: In the context of our study, which focuses on abiotic chemical reactions, we distinguish between the terms oxic/anoxic and aerobic/anaerobic. Specifically, we refer to the presence or absence of molecular oxygen in the reaction environment as oxic or anoxic conditions, whereas the terms aerobic and anaerobic are typically used to describe microbial metabolic processes. To avoid ambiguity, we have adopted this terminology consistently throughout the revised manuscript and clarified the oxygen conditions used in our experiments.

All abiotic experiments reported in this study were conducted under oxic conditions. The soil samples were first dried at 105 °C to suppress microbial activity and eliminate enzymatic processes, thereby allowing us to isolate the purely abiotic reactions under investigation. Subsequently, the soils were suspended in water to initiate the aqueous-phase iron-mediated reactions that are central to the proposed mechanism. The presence of water is essential, as iron redox cycling and associated Fenton-type reactions generate reactive oxygen species (ROS), such as hydrogen peroxide and hydroxyl radicals, which act as key oxidants driving the transformation of lignin-derived methoxy groups.

The use of wet–dry cycles was chosen for two reasons. First, the aqueous phase is required for the iron-driven oxidative reactions described above. Second, wetting–drying cycles are common environmental conditions in many soils and are known to stimulate redox fluctuations and ROS formation, which can enhance abiotic oxidation processes in soil organic matter. The wetting–drying cycle experiments were therefore designed to approximate environmentally relevant boundary conditions, under which iron-mediated oxidative reactions may occur in natural soils.

To further address the reviewer’s concern, we have also clarified in the Discussion (a new section in our manuscript) that the proposed mechanism primarily operates under oxic or periodically oxygenated conditions, such as those occurring during wetting–drying cycles. In addition, we explicitly acknowledge a limitation of the current study and have included the following statement in the revised manuscript (Discussion, lines 385–386):

“However, within the experimental framework applied here, it was not possible to directly quantify iron redox transformations, high-valent iron–oxido species, or ROS.”

We believe these revisions clarify both the environmental context of the mechanism and its dependence on oxygen availability.

Were any experiments conducted under dry or aerobic incubation conditions? This is particularly relevant given that Methylorubrum extorquens is an aerobic bacterium. You could also consider to add corresponding methanogens to utilize C1 anaerobically. Or as mentioned below in L306, just to inhibit bacteria activity to see changes of CH_3OH and CH_2O.

Authors: All abiotic experiments in this study were conducted under oxic conditions, as our objective was to investigate iron-mediated abiotic chemical reactions, which require the formation of ROS or high-valent iron–oxido species. Dry conditions alone did not result in detectable formation of CH_3OH or CH_2O, as the presence of water is essential to initiate aqueous-phase iron redox cycling and Fenton-type reactions. Therefore, soils were rewetted to trigger the abiotic processes under investigation.

To isolate abiotic formation from biological consumption, soils were dried at 105 °C prior to incubation to suppress microbial activity. In addition, we performed complementary experiments with untreated (biologically active) soils and with inoculation of *Methylorubrum extorquens*, demonstrating that CH_3OH is rapidly consumed under biologically active (aerobic) conditions. These experiments confirm that CH_3OH acts as a transient intermediate in soils.

We agree that anaerobic microbial processes, such as methanogenesis, are highly relevant for the overall C_1 cycle. However, the aim of this study was not to investigate microbial C_1 turnover pathways, but rather to identify and constrain a previously unrecognized abiotic source of oxygenated C_1 compounds. The inclusion of methanogenic systems would therefore be beyond the scope of the present work.

To clarify these aspects, we have revised the manuscript to more explicitly distinguish between abiotic oxic processes and microbial aerobic/anaerobic pathways, and to better explain the rationale for the experimental design.

2. Regarding the representativeness of the soil samples: it would be valuable to include soils with varying background levels of lignin/methoxyl groups, Fe_2O_3, and H_2O_2.

Authors: In fact, this aspect has been addressed in our experimental design. We investigated a total of 25 soil samples covering a wide range of soil types with differing TOC, methoxy group content, iron content, and pH values. The methoxy group content of the investigated soils ranges from 0.004 to 0.423 w%, and the total iron content from 0.15 to 4.13 w% (Table S1), thereby encompassing a broad range of values representative of natural soils.

To further improve clarity, we have revised Table S1 in the Supplementary Information to provide a more detailed description of all investigated soil samples and their physicochemical properties.

We agree that the inclusion in Table S1 of data related to reactive species such as H_2O_2 and high-valent iron species would provide additional mechanistic insight. However, direct quantification of these transient species in complex soil matrices was not feasible within the scope of the present study. We have therefore explicitly acknowledged this limitation in the revised manuscript and emphasize that future studies combining soil characterization with in

situ measurements of ROS and iron redox dynamics will be valuable for further constraining the mechanism.

Have you considered to employ one or two soil types and experimentally supplement them with different concentrations of these components after determining their baseline levels? Not essential to be in this study and supplementing lignin/methoxyl groups may not be necessary as you have manipulatively removing the methoxyl compounds.

Authors: We thank the reviewer for this constructive suggestion. We agree that controlled supplementation experiments (e.g., with iron or reactive oxygen species) could provide additional mechanistic insight under well-defined conditions, and we might take up these ideas in future studies. In the present study, however, we deliberately chose an alternative approach by investigating a broad range of natural soil samples without external supplementation, in order to assess whether the proposed mechanism operates under intrinsic soil conditions. By covering a wide range of methoxy group contents, iron concentrations, and pH values across 25 soils, we aimed to capture the natural variability of soil systems rather than imposing artificial conditions.

Importantly, the role of methoxy groups as the primary precursor pool was directly tested through selective removal experiments (Zeisel demethoxylation), which resulted in a complete suppression of CH_3OH formation and a strong reduction in CH_2O production. This provides direct experimental evidence for the underlying mechanism without the need for external supplementation. We agree that targeted addition experiments could be valuable for further constraining the roles of iron availability and ROS formation. However, such experiments would introduce non-natural concentrations and potentially alter the native soil chemistry. We therefore consider this approach beyond the scope of the present study and highlight it as an interesting avenue for future work.

3. The contribution of this mechanism to the overall soil carbon turnover or greenhouse gas emission should be evaluated by considering anaerobic or aerobic conditions separately.

Authors: We thank the reviewer for this important comment. We agree that distinguishing between oxic/anoxic (abiotic) and aerobic/anaerobic (microbial) conditions is essential when evaluating soil carbon turnover and greenhouse gas dynamics. In the present study, our primary objective was to identify and constrain a previously unrecognized abiotic pathway for the formation of oxygenated C_1 compounds. All abiotic experiments were therefore conducted under oxic conditions, where iron redox cycling and the formation of ROS enable the proposed demethoxylation mechanism. Consequently, the quantitative estimates provided in the manuscript refer specifically to oxic or periodically oxygenated soil environments, such as surface horizons or soils experiencing wetting–drying cycles.

We agree that under strictly anoxic (anaerobic) conditions, the proposed abiotic mechanism is expected to be strongly limited due to the absence of oxygen and ROS formation, while microbial processes such as methanogenesis are likely to dominate C_1 turnover. Specifically,

we define the observed reactivities as based on high-valent iron-oxido species, and these depend on the oxidation of iron(II) or iron(III) species by an oxygen-based oxidant (i.e., O_2, H_2O_2, or ROS resulting, e.g., from Fenton chemistry [or any oxidant when a OH_2 is coordinated to the iron species]). To clarify this point, we have added the following sentence to the revised Discussion (lines 426–428): *"The contribution of this pathway is therefore expected to be largely confined to oxic or intermittently oxygenated soils, whereas strictly anoxic environments are dominated by microbial C_1 turnover processes."*

Furthermore, to address the reviewer's concern regarding the quantitative interpretation, we have revised the Discussion to more clearly frame our global estimates as a first-order upscaling approach. Specifically, we now state that these estimates are intended to illustrate the potential magnitude of abiotic CH_3OH and CH_2O formation under oxic conditions, rather than to provide a precise quantification of global fluxes.

A full quantitative partitioning between oxic and anoxic contributions to soil carbon turnover and greenhouse gas emissions would require integrated studies combining abiotic and microbial processes across redox gradients, which is beyond the scope of the present work. However, we highlight this as an important direction for future research.

Minor comments:

L79 would anaerobic or aerobic environment matter?

Authors: Yes, the oxygen conditions are critical. In the context of our study, which focuses on abiotic chemical processes, we distinguish between oxic/anoxic conditions (presence or absence of molecular oxygen) and aerobic/anaerobic conditions (microbial metabolism). The abiotic mechanism described here relies on iron redox cycling and the formation of ROS, and therefore primarily operates under oxic conditions or during periodic reoxygenation following anoxic phases. Under strictly anoxic conditions, this pathway is expected to be strongly limited, and microbial processes are likely to dominate C_1 turnover. We have clarified this distinction and its implications in the revised manuscript.

L81 is there any other alternative methyl-based precursors?

Authors: Yes, a variety of alternative methyl-based precursors may contribute to the formation of C_1 compounds in soils. These include sulfur-, nitrogen-, and phosphorus-bound methyl groups, which can undergo abiotic or biotic transformations and may ultimately yield CH_3OH or other C_1 products. This is now discussed in more detail in a few sentences in the Introduction. However, lignin represents by far the largest source of methoxylated organic carbon in terrestrial ecosystems, and its methoxy groups constitute a particularly abundant and widespread precursor pool in soils. In the present study, we specifically focus on these lignin-derived methoxy groups because they are both quantitatively dominant and, as shown by our experiments, directly linked to the abiotic formation of CH_3OH and CH_2O: The

demethoxylation described here is quantitatively the most important process and has not been known before.

The Introduction has been substantially revised and restructured, also in response to comments from Reviewers #2 and #3, to more clearly highlight the research gap and the novelty of the abiotic demethoxylation mechanism described in this work.

L82 is there any other sources?
Authors: In addition to lignin-derived methoxy groups, other sources of methoxylated organic matter exist in soils. For example, pectin (particularly methyl-esterified homogalacturonan) contains methoxy groups and is abundant in plant tissues, especially in leaves and primary cell walls. Such compounds may also contribute to the formation of CH_3OH and potentially CH_2O in soils, although likely via different mechanisms, including enzymatic demethylation processes. In the present study, we focus on lignin-derived methoxy groups because they represent the dominant and most persistent methoxylated carbon pool in soils, and our experiments directly demonstrate their role as precursors for abiotic C_1 compound formation.

L117 why 110%?
Authors: Values exceeding 100% arise from the way conversion is normalized in our analysis. Specifically, the conversion is expressed relative to one methoxy group per molecule, whereas some substrates, such as sinapyl alcohol, contain two methoxy groups. As a result, the formation of CH_3OH and CH_2O from both methoxy groups can lead to apparent conversion values exceeding 100%. We have clarified this point in the revised manuscript and in the figure caption (Fig. 1), where the normalization procedure is now explicitly described.

L246 comparable to the natural abundances of Fe2O3 and H2O2existence in soils?
Authors: The concentrations of iron used in our experiments are broadly comparable to the range of iron contents typically found in natural soils. In contrast, the concentrations of H_2O_2 applied in the model compound experiments are higher than those expected under natural soil conditions. This difference reflects the experimental design, where elevated H_2O_2 concentrations were used to ensure controlled and measurable formation of reactive iron species and to enable mechanistic investigations under well-defined conditions. In natural soils, H_2O_2 is generated in situ at lower concentrations, for example, during wetting events and redox fluctuations. Consistent with this, the production rates of CH_3OH and CH_2O observed in soil experiments are lower than those in the model systems, supporting the interpretation that the laboratory experiments represent upper-limit, mechanistically controlled conditions, whereas the soil experiments reflect more realistic environmental rates. We have clarified this point in the revised manuscript (lines 110–113).

L255 why in water? Anaerobically or aerobically?

Authors: The experiments were conducted in water under oxic conditions. The aqueous phase is essential to enable iron redox cycling and Fenton-type reactions, which generate ROS such as H_2O_2 and hydroxyl radicals that drive the abiotic transformation of methoxy groups. Dry soils alone did not show detectable formation of CH_3OH or CH_2O, highlighting the importance of water for initiating these reactions. All experiments were performed under oxic conditions, as the proposed mechanism relies on the presence of molecular oxygen and ROS formation. This has been clarified in the revised manuscript (lines 204–206).

L256 to present information for levels of lignin, methoxy content, Fe2O3 and H2O2 in these soils?

Authors: Information on TOC, methoxy group content, iron content, and pH of all investigated soil samples is provided in Table S1 of the Supplementary Information. The methoxy group content serves as a proxy for lignin-derived material in the soils. Direct quantification of H_2O_2 concentrations in the soil samples is outside the scope of this study, specifically also due to the transient nature of ROS in complex soil matrices. However, previous studies indicate that H_2O_2 is frequently generated in situ in soils, particularly during wetting–drying events and redox fluctuations. We have clarified this in the revised manuscript and cite the corresponding studies.

L269 why wet-dry cycles? How long for each condition?

Authors: Wetting–drying cycles were chosen because they represent common environmental conditions in many soils, particularly in temperate and seasonally variable climates. Such cycles are known to induce redox fluctuations and the formation of ROS, thereby promoting iron-mediated oxidative processes. This is now discussed in detail and based on published work in the revised manuscript.

All experiments were conducted under controlled laboratory conditions (22 °C, 1013 mbar, ambient N_2/O_2 atmosphere in 50mL crimp vials) in the dark. For the soil experiments, 5 g of sterilized and milled soil was suspended in 10 mL of ultrapure water and incubated under oxic conditions for 48 h. Prior to incubation, soils were sterilized by heating at 105 °C to suppress microbial activity. Each wetting–drying cycle consisted of a 48 h wet incubation, followed by drying for 48 h before rewetting. This design simulates repeated wetting events (e.g., rainfall) and allows assessment of the persistence of abiotic CH_3OH and CH_2O formation over multiple cycles.

We have clarified both the rationale and the experimental conditions in greater detail in the revised manuscript and Methods section (lines 274-276 and 486–496).

L269.representative for what?

Authors: The selected soils, CH1 (Gleysol) and GL1 (Chernozem/Para-brown earth), represent typical temperate soil types widely distributed in mid-latitude regions. These soils differ in physicochemical properties, including total organic carbon, methoxy group content, iron

content, and pH, and were selected from the full dataset (Table S1) to capture contrasting but environmentally relevant conditions. In addition, both soils exhibit medium to high methoxy group and iron contents, allowing us to assess the sustained abiotic formation of CH_3OH and CH_2O under repeated wetting–drying cycles. We have clarified this selection rationale in the revised manuscript (lines 274–276).

L306 where is the results using dried soil samples? In wet-dry cycles?

Authors: The comparison between fresh (biologically active) and dried (sterilized and homogenized) soils was conducted using selected samples (GL1, CH2, and GL2), which are representative of the broader soil dataset. These experiments were designed to demonstrate the difference between abiotic formation and microbial consumption of CH_3OH and CH_2O. The results are included in [Figure S16 / Fig. S17], and we have now clarified in the revised manuscript (lines 259–260), which specific soil samples were used for this comparison.
We further note that the wetting–drying cycle experiments were conducted separately and are based on the soils CH1 and GL1, as described in the corresponding section.

L306 you can also inhibit bacteria activity to have a test

Authors: We thank the reviewer for this suggestion. To evaluate the influence of microbial activity on CH_3OH and CH_2O formation, we performed several complementary control experiments. First, soils were sterilized by heating at 105 °C prior to incubation to suppress microbial activity. In addition, we conducted experiments with the microbial inhibitor NaN_3, which did not significantly affect CH_3OH or CH_2O production in sterilized soils (Fig. S18), providing further evidence for an abiotic origin of the observed C_1 compounds.
Furthermore, we investigated one fresh, non-sterilized soil sample (BE1) under otherwise identical conditions. CH_3OH production was still observed, although at substantially lower levels than in the corresponding sterilized and homogenized soil. This finding demonstrates that CH_3OH formation can occur under natural conditions, while simultaneously indicating that microbial consumption acts as an efficient sink for CH_3OH and likely also CH_2O, thereby reducing their net accumulation in biologically active soils. These observations are consistent with our additional experiments using Methylorubrum extorquens, which confirmed rapid microbial consumption of CH_3OH in soils.

L326-327 that may only be applied to wetland ecosystem with frequent wet-dry cycles

Authors: Our wetting–drying cycle experiments were designed to simulate natural rainfall, rewetting, and drying events rather than conditions specific to wetlands. Such fluctuations are common in a wide range of terrestrial ecosystems, including forests, grasslands, agricultural soils, and Mediterranean-type environments, where soils regularly alternate between wetter and drier conditions following precipitation events.

Importantly, the proposed mechanism relies on the presence of water, oxygen, iron redox cycling, ROS, all of which can occur during rewetting events in non-wetland soils. The wetting–drying cycle experiments were therefore intended to assess the persistence of abiotic CH_3OH or CH_2O formation under environmentally relevant conditions that extend beyond wetland ecosystems.

We agree that the frequency and intensity of wetting–drying cycles will vary among ecosystems and may influence the overall contribution of the process. We have therefore clarified in the revised manuscript (Results lines 274-276 and Discussion, lines 419–421) that the proposed mechanism is expected to be most relevant in soils experiencing periodic wetting and reoxygenation, rather than being restricted to wetlands.

L328 how did you extrapolate?

Authors: The extrapolation was performed by scaling the experimentally determined turnover rate of CH_3OH + CH_2O production to the estimated global abundance of methoxy groups in soils. Specifically, the observed turnover rate per methoxy group was multiplied by the estimated global methoxy pool to derive a first-order estimate of potential CH_3OH and CH_2O production.

We fully acknowledge that this represents a highly simplified upscaling approach and does not account for the complexity and spatial heterogeneity of natural soils, including differences in soil composition, iron mineralogy, oxygen availability, hydrological conditions, microbial activity, and the frequency of wetting–drying events. The purpose of this calculation was therefore not to provide a precise global flux estimate, but rather to place the experimentally observed reaction rates into the context of global carbon cycling and illustrate the potential environmental relevance of the proposed mechanism.

To avoid overinterpretation, we have revised the manuscript accordingly and now explicitly state that these estimates should be regarded as first-order, order-of-magnitude approximations intended to indicate the potential importance of the process rather than its actual global flux (Discussion, lines 414-428).

L335 aerobically

Authors: To avoid ambiguity, we now consistently distinguish between oxic/anoxic conditions when referring to abiotic chemical processes and aerobic/anaerobic conditions when referring to microbial metabolism. Since the experiments discussed here are abiotic, we have revised the manuscript to refer to oxic conditions rather than aerobic conditions throughout the text where appropriate.

L340 how much?

Authors: The temperature experiments were designed primarily to assess whether the proposed abiotic pathway is temperature dependent rather than to derive a quantitative temperature sensitivity. The data clearly show increased CH_3OH production across the investigated temperature range from 6 to 50 °C (Fig. S19), increasing from approximately 5 to 31 µg g^{-1} soil dry weight, corresponding to an increase by a factor of about six. However, the number of temperature levels investigated and the associated variability do not allow a robust determination of a temperature coefficient (Q_{10}) or activation energy. We therefore limited our interpretation to the observed strong positive relationship between temperature and C_1 compound formation and refrained from deriving a quantitative temperature dependence that would not be sufficiently constrained by the available data. We added the following sentence to the manuscript (lines 454-457):
"Temperature-dependent experiments (6–50 °C) further indicate a positive relationship between temperature and abiotic C_1 compound formation (Fig. S19), suggesting that climate warming may enhance the contribution of iron- and ROS-mediated oxidation of lignin-derived organic matter in soil."

Reviewer #2

General comments:

What are the noteworthy results?

The paper present a new abiotic mechanism to degrade lignin in the soil. The process is of interest and new even though the same group has done some publications in J. Am. Chem. Soc.

Authors: We thank the reviewer for this positive evaluation of our work and for recognizing the interest and relevance of the proposed mechanism. In the revised manuscript, we have further clarified and emphasized the novelty of our findings, particularly in relation to our previous studies. Specifically, we now highlight more clearly that the mechanism described here involves full demethoxylation of aromatic methoxy groups, resulting in the release of the entire $–OCH_3$ moiety, which is fundamentally distinct from the demethylation pathways previously reported for sulfur- and nitrogen-bound methyl groups. The Introduction and Discussion sections have been revised accordingly to better delineate this mechanistic difference and to place our findings within the broader context of iron-mediated transformations of organic matter in soils. Note specifically that the demethoxylation at methoxy-substituted phenols is not only a new discovery but is extremely important due to the fact that it is much more efficient than the demethylation processes that we have described before. This is now discussed in much more clarity than in the original manuscript.

Concerns:

Will the work be of significance to the field and related fields?

The significance is not clear. It has to be better stated in a specific paragraph of the discussion section which has to be separated from the results section.

Authors: In the revised manuscript, we have substantially clarified the broader significance of our findings (see above). In particular, we have separated the Discussion from the Results section and added a dedicated paragraph that explicitly highlights the implications of the newly identified abiotic demethoxylation mechanism.

In this section, we now discuss how iron-induced demethoxylation provides a previously unrecognized abiotic pathway for the release of oxygenated C_1 compounds from lignin-derived methoxy groups under environmentally relevant conditions. We further outline the potential implications of this mechanism for soil carbon turnover, microbial C_1 metabolism, and the cycling of reactive C_1 compounds between soils and the atmosphere. These additions place our findings in the broader context of iron-mediated organic matter transformation and global carbon cycling.

We believe that these revisions substantially strengthen the presentation of the significance of our results for both environmental chemistry and soil biogeochemistry. We also note that related revisions aimed at improving the clarity of the results and their interpretation are addressed in our responses to comments from Reviewers 1 and 3.

How does it compare to the established literature?

The literature review in the part of the manuscript that follows the abstract (which should be named "introduction") is very poor. It has to be incrased also considering the general effect of iron on soil organic matter, see the work of:

Hall, S. J., & Silver, W. L. (2013). Iron oxidation stimulates organic matter decomposition in humid tropical forest soils. Global change biology, 19(9), 2804-2813.

Chen, C., Hall, S. J., Coward, E., & Thompson, A. (2020). Iron-mediated organic matter decomposition in humid soils can counteract protection. Nature communications, 11(1), 2255.

Jones, M. E., LaCroix, R. E., Zeigler, J., Ying, S. C., Nico, P. S., & Keiluweit, M. (2020). Enzymes, manganese, or iron? Drivers of oxidative organic matter decomposition in soils. Environmental Science & Technology, 54(21), 14114-14123.

Dong, H., Zeng, Q., Sheng, Y., Chen, C., Yu, G., & Kappler, A. (2023). Coupled iron cycling and organic matter transformation across redox interfaces. Nature Reviews Earth & Environment, 4(9), 659-673.

Authors: We agree that the broader context of iron-mediated soil organic matter transformation needed to be articulated more clearly. The Introduction has been substantially revised and

expanded to better integrate the existing literature on iron-driven organic matter decomposition. In particular, we now explicitly discuss the role of iron redox cycling in stimulating SOM degradation (Hall & Silver, 2013; Chen et al., 2020), the contribution of iron alongside enzymatic and manganese-driven pathways to oxidative organic matter transformation (Jones et al., 2020), and the coupling of iron cycling with organic matter turnover across redox interfaces (Dong et al., 2023). These additions position our work within the established framework of iron-mediated soil carbon dynamics and clarify the mechanistic gap addressed in this study — namely, the lack of molecular-level understanding of how iron redox chemistry can specifically transform lignin-derived methoxy groups under environmentally relevant conditions.

We further restructured the Introduction to (i) more clearly define the research gap, (ii) distinguish demethoxylation from previously reported radical-based demethylation pathways, and (iii) emphasize the significance of our findings within the broader field of iron-controlled organic matter decomposition. We believe these revisions substantially strengthen the conceptual framing of the manuscript.

Does the work support the conclusions and claims, or is additional evidence needed?

Figure 1 please provide yields on a mass basis also on a separate figure.

Authors: In the revised manuscript, we have added an additional figure in the Supplementary Information (Fig. S1) presenting the product yields on a mass basis, complementing the molar-based representation shown in Figure 1. This additional figure provides a mass balance–based perspective of product formation and allows a more direct comparison of the relative yields across the investigated substrates.

We have also included a corresponding statement in the revised manuscript (lines 114–117): "Under the applied conditions, all methoxylated aromatic compounds investigated here produced substantial amounts of CH_3OH and CH_2O (Fig. 1c; Fig. S1), with total conversion rates (100% × [mol product/mol substrate]) ranging from 10% to 110% and mass-based yields of 25.4 to 83.7 mg g^{-1} substrate (Fig. S1)."

Is the methodology sound?

Methodology is sound

Authors: We thank the reviewer for this positive evaluation.

Is there enough detail provided in the methods for the work to be reproduced?

Methods are sufficiently detailed

Authors: We thank the reviewer for the positive assessment.

Reviewer #3

General comments:

This manuscript presents an abiotic iron-induced demethoxylation mechanism for the production of oxygenated C1 compounds (CH3OH, CH2O) from lignin-derived methoxy groups under ambient conditions. The manuscript elucidates the significance of this pathway through the design of a model system, mechanistic validation, and its extension to real soil environments and the global scale. This work is well-structured and aligns with current interests in lignin valorization and bioeconomy. However, I cannot recommend acceptance of the paper in its current form due to the limited novelty of the approach and unconvincing experimental data presented. The overall quality of this work still falls far short of the standards required for publication in Nature communication.

Authors: We thank the reviewer for the careful evaluation of our manuscript and for acknowledging the relevance of the topic and the overall structure of the study. However, we respectfully disagree with her/his opinion of limited novelty (see below). However, it clearly is our fault that we have not been able to convince her/him, and we therefore have concentrated in the Introduction as well as in the Discussion and Conclusion sections to clearly show that the demethoxylation is a novel pathway and why it is of importance. In terms of the experimental evidence on which our conclusions are based, we have done a number of additional experiments, and these are discussed in detail. We also note that related revisions aimed at improving the clarity of the results and their interpretation are addressed in our responses to comments from reviewers 1 and 2.

Concerns:

1. The mechanism presented in the manuscript demonstrates limited novelty. Iron-mediated demethylation is already a well-established paradigm in both chemical and biological demethylation processes. The proposed "OAT-induced demethoxylation" in this study lacks sufficient innovation, as it essentially aligns with existing and commonly recognized reaction types.

Authors: As mentioned above, our initial presentation might not have been well enough focused and easy enough to understand. While “OAT-induced demethoxylation” is the correct description, it might be too “clumsy” for colleagues who are not specialized in mechanistic work of high-valent nonheme iron species, it might lead to misunderstanding and – while correct – it is unnecessary. We now describe three relevant mechanisms, two are demethylation processes described before, and the novel mechanism, which is a demethoxylation. We have been working on the mechanisms of iron-mediated (or iron-catalyzed) formation of C_1 (and other small organic) species for some time, and the pathways so far were demethylation reactions, i.e. formation of methyl radicals from substrates with methyl groups bound to

heteroatoms (S, N, O etc.). There were two reaction mechanisms that we have proposed (and studied in detail by product analyses, labeling studies, EPR spectroscopy, kinetics and DFT calculations), one initiated by HAA and one by OAT (the former was shown to operate with acetic acid derivatives, the latter with thioethers and sulfoxides [methionine, DMSO] as well as with methyl-substituted phosphines and amines). The reaction shown here is a third pathway, where the OMe group is detached from monolignols (methoxy phenols in general), i.e., the oxygen atom in the resulting MeOH originates from the substrate. This is a novel and completely different mechanism to those reported before. We repeat: in soil chemistry, so far there are reports of three mechanisms to produce volatile C_1 and C_2 compounds, two are demethylation reactions, and our novel discovery described here is a demethoxylation process. The additional fact of importance is that the demethoxylation reaction reported here is more efficient and in terms of produced amounts of MeOH (and follow-up products) much more important. These features, which we believe to be the most far reaching discoveries, are now described more clearly, in more detail but well focused and in short paragraphs.

Our mechanistic interpretation is based on isotope labeling and product analyses and is supported by extensive computational work, mainly involving DFT calculations. To our knowledge, such cleavage of aromatic methoxy groups under ambient, environmentally relevant conditions has not previously been demonstrated.

We consider it of importance to also mention here that Nature Comm. is a general science journal, also read by chemists and not specifically aiming at an environmental science readership. Our groups are covering environmental sciences as well as chemistry, specifically, iron-oxido and mechanistic work. While the present work is focusing on environmental problems, we have also concentrated on unambiguously analysing the mechanistic pathways, and earlier work in this collaboration has been published in chemical journals (e.g., JACS). In this context, it is of interest that a 1992 paper by Duprat et al. (cited in the manuscript) has reported an iron-mediated substitution of the methoxo groups in methoxy-phenols, albeit under different, relatively harsh, and not environmentally relevant conditions. Also, the mechanism reported was not evaluated experimentally. We are now able to show what exactly the mechanistic pathway is and how the reaction can be performed under environmentally benign conditions. This contributes to the novelty and importance of our results and discussion.

Based on the suggestions by this and the other reviewers, the revised manuscript

- explicitly differentiates *demethoxylation* from previously described *demethylation* mechanisms (Introduction, lines 67–78),
- emphasizes that the reported pathway involves aromatic $C_{aromatic}$–OR bond cleavage rather than heteroatom–C bond cleavage, and
- highlights the role of this mechanism in explaining the large amounts of CH_3OH and CH_2O relative to CH_4 formed in soils.

We believe these revisions make the mechanistic novelty and the environmental importance of the present work clearer and better position it relative to previously reported iron-mediated methyl transformations.

2. Although the manuscript emphasizes " ambient temperature and pressure conditions (22°C, 1013mbar)", the key mechanistic experiments were conducted at pH 2.3, which is inconsistent with the typical soil pH range as Table S2 showed. Additionally, high concentrations of exogenous H_2O_2 and synthetic ligand-iron complexes in the model system differ from the condition present in natural soils. So, the validity of a mechanism verified under such artificial conditions regarding its dominance in natural scenarios remains questionable.

Authors: We thank the reviewer for these comments. They have prompted us to explain to the reader, why we have chosen the specific conditions, i.e., pH values, iron, and H_2O_2 concentrations in our model studies that are not identical to those in soils. The various concentrations (pH, H_2O_2, Fe) were optimized in model studies using our bispidine system as well as Fe_2O_3 in our earlier work on methionine (Althoff 2014, Benzing 2017), and they were also identical in our more recent work (Hädeler 2023; in that publication, we also transformed our observations to soils). Hence, it was important for the present study to use the same conditions in order to be able to compare the various data sets – also the optimized parameters were leading to maximum accuracy and reproducibility. Moreover, and importantly in terms of pH, we have shown in the seminal study on Fe^{IV}=O oxidation catalysis in water (Bautz 2006) that with our bispidine system, there is formation of the ferryl species between pH 2 and 7 (i.e., also at environmentally relevant pH) and that the optimum conditions are at lower pH values, possibly at around pH 3. In terms of the Fe concentration, we note that we have shown that the methanol production is catalytic, i.e., the methanol formation does not need high "catalyst" concentrations, but the concentration obviously has an influence on the rate. This is also true for the external oxidant (H_2O_2: the H_2O_2 concentration is higher in the model studies than in soil samples; this is why the production rates are lower in soil samples). The important point is that we have confirmed that the unambiguously identified demethoxylation mechanism also works in soils, and this was confirmed using stable isotopes directly in soil samples. This clearly demonstrates the soil's capacity to demethoxylate the methoxy groups of lignin-like structures.

3. It is recommended to consider key environmental variables such as pH gradients, the content of different iron minerals, and variations in their reactivity to investigate the environmental boundary conditions of the mechanism.

Authors: This comment raises an important point. For this reason, we changed the pH of one soil (S14) and observed only a minimal change in production rate. The 25 soils that we have investigated cover a pH range of 3.2 to 7.9, which is typical for soils (and includes the range studied in our 2006 paper and the pH of our model studies presented here). They also commonly

contain iron in various forms, as different iron minerals occur in soils of such a broad pH range. The main requirement for the demethoxylation reaction is soluble iron, and this is at higher concentrations in the soil samples than in our hematite experiments. Therefore, all soils produce CH_3OH and CH_2O, the amount of which depends mainly on the TOC or methoxy content, respectively. We have added these data and facts in the methods section.

4. As illustrated in Figure S11, the correlation between either Total Organic Carbon (TOC) or methoxy group content (OCH_3) and the methoxy content with CH_3OH+CH_2O production in sterile soils, the R^2 values are only 0.67 or 0.60, respectively. This level of correlation appears insufficiently compelling. It is recommended to consider incorporating additional evaluation parameters for a more comprehensive analysis such as residual plot or others. Additionally, it is recommended to consider the relationship between the concentrations of H_2O_2 and high-valent iron species during the process and the generation of CH_3OH/CH_2O.

Authors: We agree that the observed coefficients (R^2 = 0.60–0.67) represent moderate correlations. However, we note that these data are derived from natural soil samples spanning a wide range of physicochemical properties, including variations in organic matter composition, iron mineralogy, and redox dynamics. In such heterogeneous systems, correlations of this magnitude are commonly observed and can still be considered meaningful, particularly when consistent trends are evident across multiple independent samples.

Importantly, the correlation between TOC and methoxy group content is substantially stronger (R^2 = 0.88; Fig. S12), supporting the interpretation that methoxy groups ─ primarily derived from lignin ─ represent a major fraction of soil organic carbon relevant to C_1 compound formation. *Furthermore*, the role of methoxy groups as the primary precursor pool is independently confirmed by our methoxy removal experiments, where CH_3OH formation was completely suppressed, and CH_2O production was strongly reduced following Zeisel demethoxylation. These results provide direct mechanistic evidence that complements the statistical correlations.

Following the reviewer's suggestion, we have evaluated additional statistical approaches, including residual analysis. However, due to the limited number of samples and the intrinsic variability of natural soils, these analyses did not provide additional meaningful insights beyond the reported correlations. We have therefore retained the current presentation but clarified in the revised manuscript that the correlations should be interpreted in the context of complex, multi-factorial soil systems, where multiple variables influence C_1 compound formation.

Regarding the relationship between H_2O_2 concentrations, high-valent iron species, and CH_3OH/CH_2O formation, we agree that these parameters are highly relevant for constraining the mechanism. However, Fe^{IV}=O species are intrinsically reactive, and it is not possible to quench them under the conditions of our experiments (the corresponding bispidine complexes, e.g., have a half-life of only a few minutes at -80 °C). We have now explicitly acknowledged this limitation in the revised Discussion.

We believe that, taken together, the combination of consistent correlations, strong TOC–methoxy linkage, and direct experimental evidence provides robust support for the proposed role of methoxy groups in abiotic C_1 compound formation in soils.

5. In natural soils, lignin-carbohydrate complexes and partially degraded natural lignin fragments are more widely distributed than lignin itself. The lack of experiments involving these components may reduce the reliability of extrapolating the conclusions to soil environments.

Authors: We agree that in natural environments lignin rarely occurs as intact polymeric lignin, but rather as partially degraded fragments and lignin–carbohydrate complexes. However, the mechanism investigated in our study targets the methoxy functional groups (–OCH_3) bound to aromatic structures, which remain abundant in these lignin-derived materials regardless of the specific macromolecular architecture.

Our experimental design, therefore focused on the reactive functional group level, rather than the full structural complexity of soil organic matter. To address this systematically, we used a hierarchical experimental approach that includes:

1. Well-defined lignin monomers (monolignols and methoxyphenols) to establish the underlying reaction mechanism under controlled conditions.
2. Polymeric lignin, representing a more complex lignin-derived substrate.
3. Natural soil samples, which inherently contain mixtures of lignin fragments, lignin–carbohydrate complexes, and other methoxylated organic matter.

Importantly, the soil experiments show the same qualitative pattern of oxygenated C_1 compound formation (CH_3OH and CH_2O) observed in the model systems, supporting the environmental relevance of the proposed mechanism. Furthermore, when methoxy groups were chemically removed from the soils using the Zeisel method, the production of CH_3OH was eliminated, and CH_2O formation was strongly reduced. This demonstrates that methoxy groups in soil organic matter represent the primary precursors of the observed oxygenated C_1 compounds, independent of whether they originate from intact lignin, lignin fragments, or lignin–carbohydrate complexes.

Based on these combined results, we believe that the functional-group–based approach used here provides a robust basis for extrapolating the identified abiotic demethoxylation mechanism to natural soil systems. We have clarified this rationale in the revised Discussion section (lines 359–356).

6. Can control experiments be established for the soil tests? Since soil sterilization may not completely eliminate certain unknown biologically active enzymes or the release of microbial intracellular metabolites. Consideration might be given to adding enzyme inhibitors and using artificially constructed soils as controls.

Authors: We agree that residual enzymatic activity or intracellular metabolites potentially released during sterilization should be considered when interpreting the soil experiments. To address this concern, we performed additional control experiments with three representative soil samples (BE1, GL1, and CH1) in which sodium azide (NaN_3), a potent inhibitor of enzymatic activity and microbial metabolism, was added following sterilization.

The addition of NaN_3 did not result in significant changes in CH_3OH and CH_2O production compared to the corresponding sterilized controls, with the exception of a slight increase observed for soil CH1 (Fig. S18). Overall, the results remained within the experimental variability and provide additional evidence that the observed formation of CH_3OH and CH_2O is not attributable to residual enzymatic activity or intracellular metabolites persisting after sterilization. We have included these new experiments in the revised manuscript (lines 368 bis 372) and Supplementary Information (Fig. S18).

Regarding artificially constructed soils, we agree that such systems may provide additional mechanistic insights by allowing tighter control over individual soil components. However, the aim of the present study was to investigate whether the proposed mechanism operates under environmentally relevant conditions in natural soils. We therefore consider artificial soil systems an interesting avenue for future studies.

7. Line 268: Please add the reasons for selecting these two soil samples CH and GL1 for the wet-dry cycles experiments.

Authors: In total, we investigated 25 soil samples covering a wide range of soil types with differing TOC, methoxy group content, iron content, and pH values (Table S1). From this dataset, we selected two contrasting soils ─ CH (Gleysol; now referred to as CH1) and GL1 (Chernozem/Para-brown earth) ─ for the wet–dry cycle experiments.

These soils were chosen because they represent distinct physicochemical characteristics, including differences in organic carbon content, methoxy group abundance, iron content, and pH. This allowed us to assess whether the observed abiotic formation of CH_3OH and CH_2O persists under repeated wetting–drying conditions across contrasting soil environments, rather than being limited to a specific soil type. We have added a clarifying sentence to the revised manuscript (lines 272–276) to explain the rationale for this selection.

8. Please add error bars to the lower segments of the stacked columns in all bar charts, such as Figure 1, Figure 3(a), Figure S1, Figure S3, Figure S10, Figure S12(b), Figure S14, Figure S19.

Authors: We thank the reviewer for this suggestion. In our figures, CH_3OH and CH_2O are presented as stacked columns because both compounds originate from the same methoxy-group precursor and are therefore discussed and interpreted jointly throughout the manuscript. For this reason, we chose to display a single error bar representing the combined CH_3OH + CH_2O

production, which reflects the overall variability of the conversion process and avoids unnecessary visual complexity in the stacked bar charts.
To improve transparency, we have clarified in the figure captions how the error bars were calculated. In addition, the corresponding uncertainties for CH_3OH and CH_2O are now provided separately in the Appendix including the complete dataset. This allows readers to assess both the variability of the individual compounds and the overall variability of methoxy-group conversion.

9. The descriptive sentence for Table S1 on page 2 of the Supplementary Materials is incomplete.

Authors: The Supplementary Information has been carefully revised, and the descriptive sentence for Table S1 has been corrected and completed. In addition, we have thoroughly checked the entire Supplementary Information for consistency and clarity.

10. The text on page 14 of the Supplementary Materials incorrectly refers to "Table S2" when it should be "Table S1".

Authors: Corrected.

11. The y-axis in Figure S11(b) is not fully displayed.

Authors: Corrected.
